# Housing inequalities and health outcomes among migrant and refugee populations in high-income countries: a mixed-methods systematic review

**DOI:** 10.1186/s12889-025-22186-5

**Published:** 2025-03-22

**Authors:** Kritika Rana, Jennifer L. Kent, Andrew Page

**Affiliations:** 1 Translational Health Research Institute, Western Sydney University, Campbelltown, NSW 2560 Australia; 2 The University of Sydney School of Architecture, Design and Planning, The University of Sydney, Sydney, NSW 2008 Australia

**Keywords:** Housing, Health, Migrant, Refugee, High-income countries, Systematic review

## Abstract

**Background:**

Migrant and refugee populations are disproportionately affected by the housing crises reportedly impacting high-income countries around the globe. However, the health implications of housing inequalities within these communities and contexts remain relatively understudied. This review aimed to synthesise the evidence on housing and health inequalities prevalent among migrant and refugee populations in high-income countries, and to identify the pathways linking housing inequalities and health outcomes.

**Methods:**

This systematic review employed the Joanna Briggs Institute (JBI) methodology for mixed-methods systematic reviews using a convergent integrated approach to synthesis and integration. Electronic database searches were conducted using Medline (OVID), Web of Science (ISI), Embase (OVID), PsycInfo (OVID), Scopus, and CINAHL (EBSCO), supplemented by grey literature searches on Google Scholar, MedNar, and WHOLIS. Eligible studies included quantitative, qualitative, and mixed methods designs focused on understanding how housing inequalities are associated with physical and mental health outcomes.

**Results:**

A total of 65 studies published between 1995 and 2024 were included in this review, comprising 38 quantitative and 27 qualitative studies. Substandard housing conditions, such as overcrowding and poor ventilation, were consistently associated with adverse physical and mental health outcomes, including respiratory illnesses and experiences of anxiety and depression. The type of housing tenure also impacted both physical and mental health, specifically living in inadequate rental housing as opposed to self-owned homes, was linked with poorer physical health and increased risk of mental health issues. Similarly, housing insecurity stemming from unstable housing situations and insecure tenancy, as well as neighbourhood conditions such as safety concerns and living in deprived neighbourhoods, led to the exacerbation of both physical and mental health issues. Furthermore, housing affordability challenges and decreased housing satisfaction were linked with poor mental health outcomes such as experiences of depression and psychological distress.

**Conclusions:**

This review highlights the critical role of housing as a social determinant of health and wellbeing for migrant and refugee populations in high-income countries, along with highlighting the potential pathways through which housing inequalities impact physical and mental health outcomes. Ensuring access to adequate, affordable, and secure housing, while also improving neighbourhood conditions, is essential for improving the health and wellbeing of migrant and refugee populations.

**Supplementary Information:**

The online version contains supplementary material available at 10.1186/s12889-025-22186-5.

## Introduction

Adequate housing is a fundamental human right and an essential component of human wellbeing [1, 2], encompassing various elements such as security of tenure, availability of services, affordability, habitability, accessibility, location, and cultural adequacy [3]. However, several high-income countries, including Australia [4, 5], the United States [6, 7], and various European nations [8, 9], are facing a housing crisis characterised by declining housing affordability and increasing homelessness. The housing crisis has been exacerbated by factors such as rising house and land prices in the aftermath of the global financial crisis, along with a growing disparity between median house sales prices and median household incomes [7, 8, 10].

The negative ramifications of the housing crisis are disproportionately experienced by socioeconomically disadvantaged and ethnic minority groups, and migrants and refugees constitute a significant proportion of this demographic in high-income countries [6, 11, 12]. Migrants and refugees may encounter additional challenges in accessing affordable and secure housing due to racial discrimination and precarious legal status, along with the long waiting lists for social and public housing and a diminishing market of affordable private housing, which further exacerbate their housing stress [12, 13]. In Australia, for example, over half of all low-income households spend more than 30% of their gross income on housing costs [14], with recently arrived humanitarian migrants reported to be twice as likely to struggle with housing payments compared to others [15]. Consequently, migrant and refugee populations are often confined to socioeconomically disadvantaged areas in search of affordable rental housing, leading to a cycle of cumulative disadvantage [16].

The relationship between housing and health is complex and bidirectional, and adequate, affordable, and secure housing is essential for maintaining good health and wellbeing [17, 18]. Tangible physical housing defects, such as toxins, cold indoor temperatures, dampness, mould, overcrowding, and safety factors, have been linked to negative physical and mental health outcomes [19–21]. Additionally, less tangible aspects of housing, such as affordability, tenure stability, and housing satisfaction, have also been shown to influence health outcomes [22]. Despite the recognised importance of housing as a social determinant of health, the relationship between housing and health among migrant and refugee populations in high-income countries remains relatively understudied [20]. Housing has significant implications for the health and wellbeing of disadvantaged populations, including migrants and refugees, with housing inequalities leading to significant health disparities across housing-related health outcomes [6, 23].

Previous systematic reviews exploring the relationship between housing and health among refugee and asylum-seeking populations, while valuable, do not provide a complete picture of the broader migrant and refugee population’s experiences [24, 25]. For instance, one review focused exclusively on refugee and/or asylum-seeker populations, excluding studies that referred to “migrants” or “immigrants”, and only considered studies published up until 2017 [24]. Another review was limited to examining the psychosocial attributes of housing associated with health among refugee and asylum-seeking populations in high-income countries [25]. These research gaps highlight the need for a comprehensive and updated systematic review that captures the full spectrum of housing-related health inequalities across migrant and refugee populations in high-income countries, and examines the association between housing inequalities and health outcomes within this broader group. Therefore, this mixed-methods systematic review aimed to synthesise the evidence on the key housing and health inequalities prevalent among migrant and refugee populations in high-income countries, and to identify the pathways linking housing inequalities and health outcomes.

## Methods

### Protocol and registration

This systematic review employed the Joanna Briggs Institute (JBI) methodology for mixed-methods systematic reviews using a convergent integrated approach to synthesis and integration, and the Preferred Reporting Items for Systematic review and Meta-Analysis Protocols (PRISMA-P) statement [26, 27]. The protocol of this systematic review has been previously published [28], and is also registered with the PROSPERO International Prospective Register of Systematic Reviews (CRD42022362868).

### Eligibility criteria

Using the Population of interest, Intervention(s), Comparator(s), Outcome(s) and study designs (PICOS) framework [29], the eligibility criteria outlined in Table 1 were defined, and a single review question for both quantitative and qualitative studies was formulated: *Among migrant and refugee populations in high-income countries*,* how are housing inequalities associated with physical and mental health outcomes?* Quantitative (e.g., observational studies including cross-sectional, longitudinal, cohort, and intervention studies); qualitative (e.g., ethnography, grounded theory, phenomenology, and action research); and mixed methods designs were all considered for inclusion. All empirical studies published in English language in both peer-reviewed scholarly and grey literature were considered for inclusion.

**Table 1:** Eligibility criteria for quantitative and qualitative studies

Parameter		Criteria
***Quantitative Studies***		
**P**	Population and setting	Migrants and refugees (as defined by the International Organization for Migration [32]) of all age groups in high-income countries (as defined by the World Bank [30])
**I**	Exposure (independent variable)	Measures of housing quality* (including tangible and non-tangible aspects). Tangible factors include (but are not limited to) housing conditions or characteristics (e.g., quality of the physical structure; over-crowding, or number of people per room; access to accommodation or residential mobility; and internal characteristics such as heating and cooling). Non-tangible factors include (but are not limited to) housing affordability, tenure stability and housing satisfaction.
**C**	Comparison	None or population subgroups with differences in measures of housing quality
**O**	Outcome (dependent variable)	Physical and mental health outcomes (e.g., physical and mental health status; prevalence of specific health conditions such as anxiety, depression and post-traumatic stress disorder)
***Qualitative Studies***		
**P**	Population and setting	Migrants and refugees (as defined by the International Organization for Migration) of all age groups
**I**	Interest	Experiences and perceptions related to the association between housing inequality and health outcomes
**Co**	Context	High-income countries (as defined by the World Bank)

* Inequalities in measures of housing quality will be used to determine housing inequalities

The population of interest in this review includes migrants and refugees residing in high-income countries, as defined by the World Bank [30]. “Migrants and refugees” were defined as groups of people traveling in mixed movements, who may have multiple, overlapping reasons for moving, as outlined by the United Nations High Commissioner for Refugees’ (UNHCR) [31]. According to the International Organization for Migration’s (IOM), a “migrant” was defined as any individual who moves across an international border away from his or her habitual place of residence, irrespective of legal status, including authorised migrants for purposes such as work, family and study, as well as unauthorised entrants, including asylum seekers, and irregular or undocumented migrants [32]. A “refugee” is defined by IOM as an individual who has obtained refugee status or humanitarian protection or is fleeing persecution or organised violence [32].

### Search strategy and information sources

A comprehensive search strategy was developed in consultation with two health-sciences librarians, using a combination of specific medical subject headings (MeSH), free-text words and Boolean operators. The search strategy was pretested in Medline (OVID) and the syntax and subject headings were adapted for all other databases (Supplementary Table S1). The following electronic databases were searched without any restriction on publication date: Medline (OVID), Web of Science (ISI), Embase (OVID), PsycInfo (OVID), Scopus and Cumulative Index to Nursing and Allied Health Literature (CINAHL) (EBSCO). The electronic database search was supplemented by grey literature searches using Google Scholar, MedNar and WHOLIS. A manual search of the reference lists of the eligible studies and previously published systematic reviews were also undertaken, including backward and forward citation tracking of the included studies. The initial search was conducted in November 2022, followed by an updated search in December 2024.

### Study selection

Studies identified through the electronic databases were collated into EndNote 20 reference management software (Clarivate Analytics, Philadelphia, PA, USA). Subsequently, the references were exported to Covidence systematic review management software (Veritas Health Innovation, Melbourne, Australia) [33] and duplicate records removed. Titles and abstracts of identified studies were screened according to the inclusion criteria. Articles that met the inclusion criteria or required further assessment were retrieved in full text. Potential studies identified through the grey literature and manual search were also retrieved in full text. All full-text articles were assessed against the inclusion criteria, and the reasons for exclusion were recorded (Supplementary Table S2). Screening of the studies was conducted by one reviewer and cross-verified by a second reviewer. Any disagreements were resolved through consensus, or if necessary, discussions with a third reviewer. The study selection process adhered to the Preferred Reporting Items for Systematic Reviews and Meta-Analysis (PRISMA) checklist and has been presented in the form of a flow diagram [27].

### Assessment of methodological quality

Identified quantitative and qualitative studies were assessed by two independent reviewers for methodological validity using standardised critical appraisal instruments from JBI [26]. Any disagreements were resolved through consensus, or if necessary, in discussions with a third reviewer. The results of the critical appraisal included information on methodological quality of each study, and the potential influence of methodological quality on the interpretation of study results. Based on the results of the quality assessment, the following categories were established: low risk of bias (scoring ≥ 70%), moderate risk of bias (scoring 50–69%), and high risk of bias (scoring 0–49%) [34].

### Data extraction

Standardised data extraction templates were developed for quantitative and qualitative studies. The data extracted from quantitative studies included information on study details, population and setting, study aims, exposure and outcomes/measures in the study, statistical methods and results/effect estimates, and the author’s conclusions and reviewer’s comments. The data extracted from qualitative studies included information on study details, population and setting, study design and methods, study aims, main themes and subthemes/narrative description, and author’s conclusions and reviewer’s comments. Quantitative and qualitative data were extracted from studies included in the review by two independent reviewers. Any disagreements were resolved through consensus, or if necessary, discussions with a third reviewer.

### Data synthesis

A convergent integrated approach to synthesis and integration was employed for mixed-methods systematic reviews [26]. This approach involved transformation of quantitative data into “qualitised data”, through textual descriptions or narrative interpretations of the quantitative results, to respond directly to the review question. Subsequently, the “qualitised data” were assembled with the qualitative data extracted from qualitative studies. The combined data were then categorised and pooled based on similarity in meaning to generate a set of integrated findings.

## Results

### Search results

**Fig. 1:**
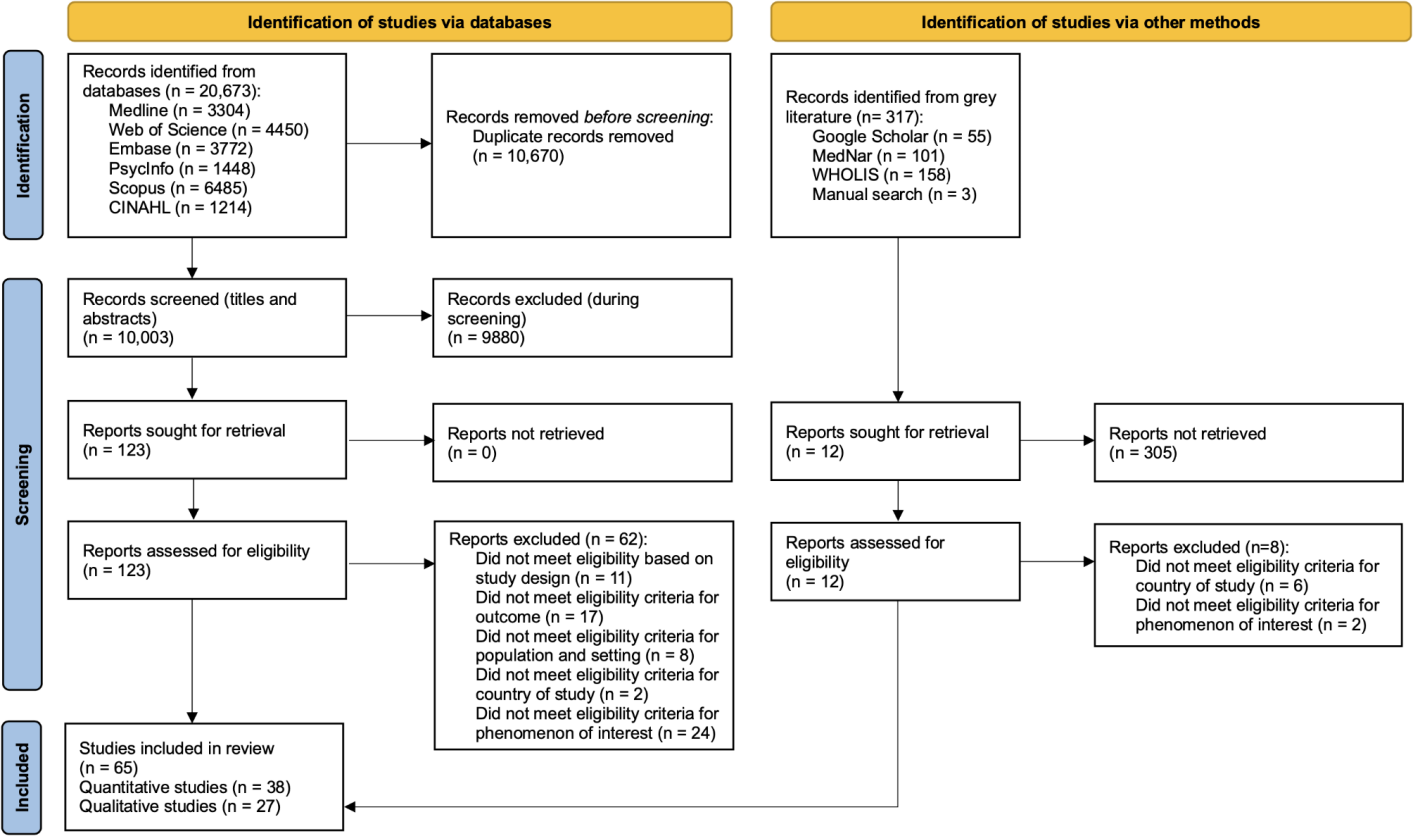
PRISMA flow diagram of the study selection process

A total of 20,673 records were identified from electronic databases, and after removal of duplicate records, the titles and abstracts of 10,003 records were screened. Full-text reports of 123 studies were assessed for eligibility, of which 62 reports were excluded. Additionally, 317 records were identified from the grey literature, of which 12 records were retrieved in full text and assessed for eligibility, and 8 reports were excluded. The reasons for excluding the 70 full-text studies are detailed in Supplementary Table S2. Overall, 61 studies were included from electronic databases and 4 studies from the grey literature, resulting in a total of 65 studies included in this systematic review. The PRISMA flow diagram illustrating the study selection process is presented in Fig. 1.

### Overview of included studies

**Fig. 2:**
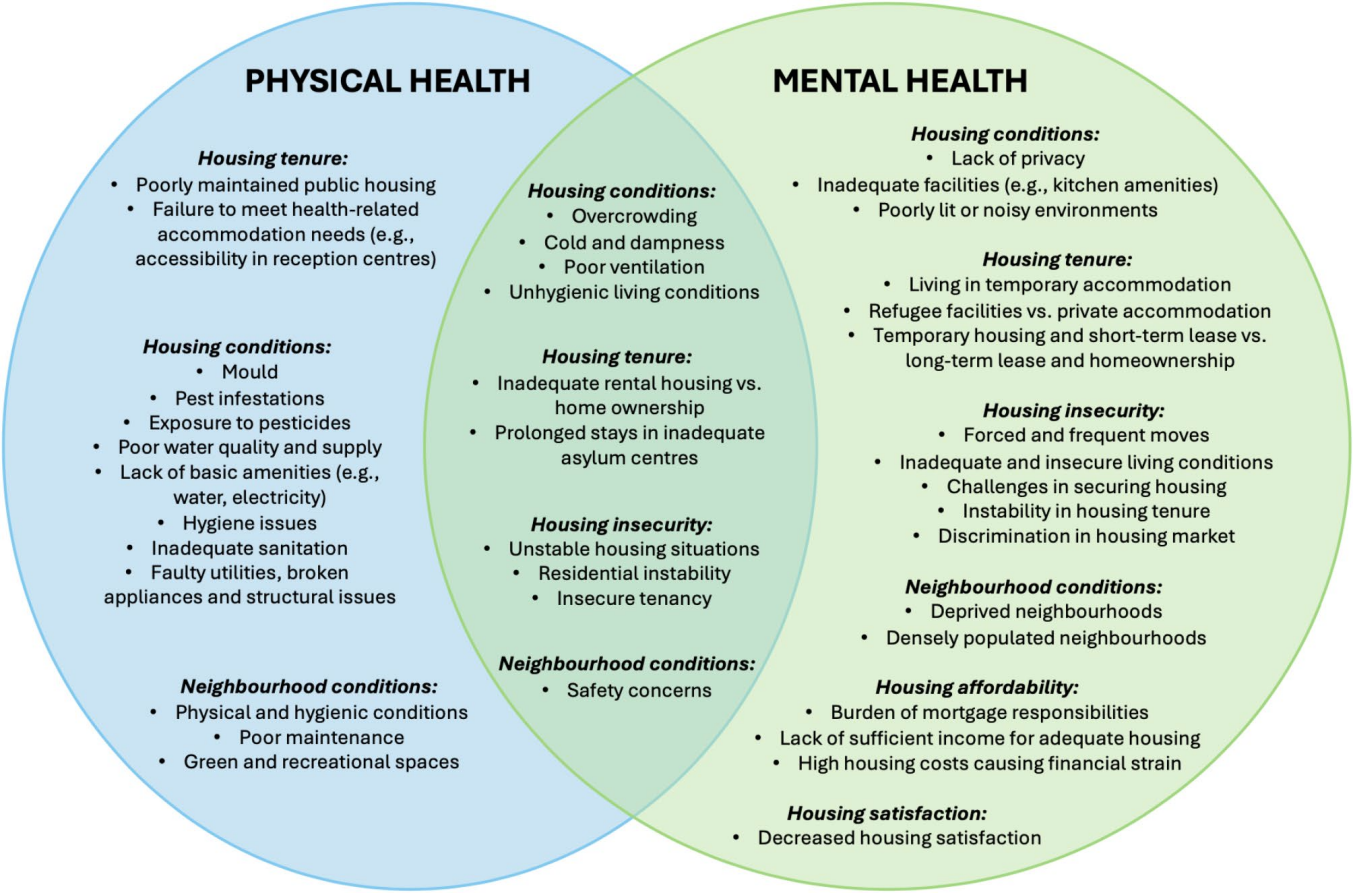
Intersection between housing factors and physical and mental health among migrant and refugee populations in high-income countries

Of the 65 studies included in this systematic review, 38 (58.5%) were quantitative studies [35–72] and 27 (41.5%) were qualitative studies [73–99]. Table 2 provides an overview of the studies included in the review by study design. The studies were published between 1995 and 2024, with 25 (38.5%) studies published between 2010 and 2019 and 32 (49.2%) studies between 2020 and 2024. The majority of the studies included migrants and refugees from the United States [44, 45, 49, 50, 58, 62–64, 71, 81, 83, 86], followed by Australia [46, 47, 53, 74, 76, 77, 80, 87, 94, 98, 99], and Germany [36, 37, 43, 52, 55, 56, 69, 70, 90, 92]. A total of 33 (50.8%) studies focused exclusively on refugee populations, while 32 (49.2%) studies either focused exclusively on migrants or included both migrants and refugees in their target population. As shown in Table 2, the assessed housing factors varied across studies, with 24 (36.9%) focusing on tangible factors (such as housing conditions or characteristics), 20 (30.8%) on non-tangible factors (such as housing affordability and housing satisfaction), and 21 (32.3%) studies on both. In terms of health outcomes, mental health was the primary focus in 35 (53.8%) studies, while physical health was examined in 15 (23.1%) studies, and 15 (23.1%) studies considered both. Figure 2 illustrates the intersection between housing factors and physical and mental health identified in this review. The characteristics and summary of the quantitative and qualitative studies reviewed are presented in Supplementary Table S3 and S4, respectively.

**Table 2:** Overview of studies included in the review

Characteristics	Quantitative studies (*n* = 38)	Qualitative studies (*n* = 27)	Total (*n* = 65)
**Year of publication**			
Before 2000	1	-	1 (1.5%)
2000–2009	-	7	7 (10.8%)
2010–2019	14	11	25 (38.5%)
After 2020	23	9	32 (49.2%)
***Country of study*** *			
Australia	3	8	11 (16.9%)
Canada	5	3	8 (12.3%)
France	3	1	4 (6.2%)
Germany	8	2	10 (15.4%)
Sweden	7	-	7 (10.8%)
UK	2	4	6 (9.2%)
US	9	3	12 (18.5%)
Other countries**	7	6	13 (20.0%)
***Target population***			
Migrants (may include refugees)	22	10	32 (49.2%)
Exclusively refugees	16	17	33 (50.8%)
***Assessed housing factors*** ***			
Tangible factors	16	8	24 (36.9%)
Non-tangible factors	13	7	20 (30.8%)
Tangible and non-tangible factors	9	12	21 (32.3%)
***Health outcomes focused***			
Physical health	11	4	15 (23.1%)
Mental health	22	13	35 (53.8%)
Physical and mental health	5	10	15 (23.1%)

UK: United Kingdom; US: United States

* Total number of studies may not add up as three studies were conducted across multiple countries

** Other countries include Chile (*n* = 3), Belgium (*n* = 2), Italy (*n* = 2), New Zealand (*n* = 2), Norway (*n* = 2), Qatar (*n* = 2), Saudi Arabia (*n* = 2), South Korea (*n* = 1) and Spain (*n* = 1)

*** Tangible factors include (but are not limited to) housing conditions or characteristics (e.g., quality of the physical structure; over-crowding, or number of people per room; access to accommodation or residential mobility; and internal characteristics such as heating and cooling). Non-tangible factors include (but are not limited to) housing affordability, tenure stability and housing satisfaction

### Association between housing inequalities and physical health outcomes

A total of 30 (46.2%) studies reported on the link between housing and physical health, with most studies revealing a strong association between housing inequalities and poor physical health outcomes among migrant and refugee populations residing in high-income countries.

#### Housing conditions and physical health

Housing conditions were identified as an essential factor impacting physical health outcomes [39, 49, 50, 54, 56, 59, 61, 75, 76, 81–83, 90, 95, 98, 99]. Several studies highlighted a consistent association between poor housing conditions, such as cold, dampness, mould, and pest infestations, and a range of health conditions, including asthma, headaches, and other respiratory conditions [49, 50, 59, 61, 83, 95, 98, 99]. Especially among migrant farmworkers in the United States, adverse health outcomes attributed to substandard housing conditions were reported [49, 81, 83], with indoor environmental risk factors such as the presence of mould and use of pesticides for pests, contributing to respiratory issues such as coughing up phlegm and asthma [49]. Increased exposure to environmental hazards due to the proximity of housing to pesticide-treated agricultural fields led to systemic symptoms such as headaches and nausea, as well as higher rates of occupational injuries and chronic health problems, including respiratory issues and skin conditions [81, 83]. Moreover, the migrant farmworkers faced a range of health issues attributed to substandard housing conditions, including safety risks from faulty utilities, gastrointestinal discomfort and diseases from poor water quality and inadequate sanitation, and a higher incidence of communicable diseases due to the exposure to harsh living conditions [81, 83]. In a study focused on children from immigrant families in the United States, suboptimal housing conditions with poor ventilation, mould, and pests were significantly associated with increased incidence of respiratory and atopic symptoms [50]. Similarly, studies conducted among children from immigrant families in Sweden showed associations between dampness and mould with asthma, headaches, and atopic sensitisation, while the presence of cockroaches was associated with colds and emergency care visits [59, 61]. Studies among migrants in New Zealand revealed that the traditional practice of living as an extended family led to overcrowded conditions with inadequate ventilation, which was associated with health conditions such as asthma, respiratory illnesses, and skin infections [82, 95]. Inadequate housing conditions, characterised by the lack of basic amenities such as water and electricity, poor shelter quality with insufficient insulation and ventilation, and inadequate hygiene and sanitation facilities, has been shown to significantly contribute to adverse physical health outcomes, including increased health problems and disease spread among residents in informal settlements and refugee camps [54, 75]. Conversely, improved housing conditions with access to sanitation facilities, a reliable water supply, and proximity to public transportation, have been associated with better health outcomes, including a reduced likelihood of both short-term and long-term healthcare needs [39]. Additionally, unsuitable physical environments, such as overcrowding, cold and damp conditions, inadequate housing maintenance, including broken appliances and structural issues, as well as the condition of gardens and outdoor spaces, were found to impact the overall health and wellbeing of refugees and asylum seekers in Australia [76, 99].

#### Housing tenure and physical health

Several studies highlighted the impact of housing tenure on physical health [66, 74, 85, 90, 95]. A study conducted in Norway found that prolonged stays in inadequate asylum centres, marked by poor living conditions and limited access to activities, significantly deteriorated the health and wellbeing of asylum-seeking families [85]. Another study among asylum seekers and refugees living in German reception centres found that overcrowded and poorly maintained conditions negatively affected residents’ physical health, increasing their vulnerability to illness due to hygiene issues, as well as health risks from failure to meet individuals’ health-related accommodation needs, such as accessibility and dietary requirements [90]. Moreover, Sundquist et al. demonstrated that migrants and refugees in Sweden had higher odds of self-rated poor health compared to matched native Swedes, attributed to differences in living conditions such as crowded living in rented flats as opposed to private home ownership [66]. Similarly, refugees in Australia living in poorly maintained public housing were found to face health issues related to overcrowding and infestations [74]. In a study focused on migrants in New Zealand, challenges in the private rental market, such as inadequate heating, dampness, and pest infestations, were found to be associated with respiratory problems such as asthma and chest infections [95]. In contrast, living in a self-owned home was linked to improvements due to the reduction of health risks associated with poor rental conditions [95].

#### Housing insecurity and physical health

Housing insecurity emerged as an essential factor influencing physical health outcomes [78, 89, 99]. Migrant women facing housing insecurity in Canada were found to struggle with health issues such as migraines, high blood pressure, and reproductive problems, which directly contributed to their housing instability [78]. Moreover, the stress from unstable housing situations further led to chronic pain, vision loss, and hormonal imbalances, hindering their ability to work and improve living conditions [78]. Similarly, a study exploring housing inequalities among refugees in the UK found that insecure tenancy and financial constraints associated with housing impacted their ability to maintain a healthy lifestyle, including access to nutritious food and healthcare services [89]. Another study among refugees and asylum seekers in Australia highlighted the issue of housing insecurity, which was linked to physical health issues, including breathing difficulties and sleep problems, while stable housing was associated with improved wellbeing [99].

#### Neighbourhood conditions and physical health

Several studies highlighted the significant impact of neighbourhood conditions on physical health [48, 76, 79, 99]. Haque and Rosas demonstrated that various elements of neighbourhood are interconnected and are linked with negative health outcomes among migrants in Canada, particularly safety concerns, physical and hygienic conditions, poor maintenance, and lack of green and recreational spaces [79]. Similarly, migrants and refugees in an Australian study who had experienced unsafe neighbourhoods reported negative impacts on their family’s health, while those living in neighbourhoods with accessible greenery, such as parks and open spaces, expressed greater satisfaction and a sense of healthy living [76]. Moreover, residential environment satisfaction has been found to be associated with self-rated health, with immigrant workers in South Korea expressing mid or unsatisfied levels more likely to report poorer health compared to those who were satisfied [48].

### Association between housing inequalities and mental health outcomes

The relationship between housing and mental health was explored in 50 (76.9%) studies, with most studies demonstrating a strong association between housing inequalities and poor mental health outcomes among migrant and refugee populations residing in high-income countries.

#### Housing conditions and mental health

Housing conditions were identified as a critical factor influencing mental health outcomes [39, 51, 55, 58, 71, 73, 76, 88, 89, 91, 95, 97–99]. Crowded living conditions were particularly associated with poor mental health outcomes among migrants and refugees, including experiences of anxiety and depression [51, 58]. In contrast, stable and uncrowded housing was linked to improved mental health outcomes among asylum seekers in the United States, predicting lower levels of depression, anxiety, and post-traumatic stress disorder (PTSD) symptoms [71]. In addition to over-crowding, other poor housing conditions, including lack of privacy, inadequate facilities, and unhygienic conditions were also found to negatively impact mental health in several studies, leading to experiences of stress, anxiety, depression and other mental health issues [55, 73, 88, 89, 91, 97, 98]. Moreover, a study focused on migrants and refugees in South Australia found that heightened stress from cramped living conditions and traumatic triggers from poorly lit or noisy environments, compounded by limited sense of autonomy over their living situations, adversely affected psychological wellbeing [76]. Another Australian study also demonstrated that unsuitable physical environments, such as overcrowding, cold and damp conditions, and noisy locations, impacted the mental health of refugees and asylum seekers by exacerbating stress and ontological insecurity, while improvements in housing conditions fostered better health outcomes [99]. Similarly, a study conducted in New Zealand among Kiribati migrants found that stress and anxiety due to living in overcrowded conditions with poor ventilation, combined with their struggles to provide better living conditions for their families, led to feelings of helplessness and negatively impacted their mental wellbeing [95].

#### Housing tenure and mental health

The findings across several studies indicate a consistent association between housing tenure and mental health outcomes [36, 38, 43, 46, 47, 56, 57, 65, 69, 70, 72, 85, 90, 95]. Studies among migrant populations in Canada and Spain found that individuals residing in rental housing or shared accommodations were at an increased risk of mental health issues and depressive symptoms compared to homeowners [38, 57, 65]. Similarly, studies among recently arrived humanitarian migrants in Australia also showed a link between unstable housing tenure and poor mental health outcomes, with temporary housing and short-term leases associated with a higher likelihood of PTSD, while stable housing tenure, such as long-term leases and homeownership, was linked to lower psychological distress [46, 47]. Among migrants in New Zealand, the transition from inadequate rental housing to owning a home was found to positively impact mental health by reducing stress and anxiety [95]. Wirehag et al. reported that living in temporary accommodation was linked to elevated scores on scales for depression, anxiety, and PTSD among undocumented migrants in Sweden [72]. Studies conducted among refugees in Germany have also identified the type of housing (i.e., rented house, rented apartment, and refugee camp) as a significant predictor of psychosocial health [36], with individuals living in refugee housing facilities experiencing higher levels of psychological distress compared to those in private accommodation [69, 70], and collective accommodation being associated with poorer mental health scores than private accommodation [43]. Additionally, Lauritzen and Sivertsen highlighted the detrimental effects of prolonged stays in inadequate asylum centres in Norway, leading to mental health issues such as depression and anxiety [85]. The type of refugee accommodation was also found to be associated with mental health outcomes in a study conducted in Germany, where accommodations with moderate occupancy, minimal deterioration, and a central urban location were linked to lower levels of depression and generalised anxiety disorder [56]. Another German study among asylum seekers and refugees reported that life in reception centres was characterised by shared and crowded living spaces, restricted autonomy, lack of privacy, uncertainty, and frequent relocations, all of which were stressors negatively impacting mental health and psychological wellbeing [90].

#### Housing insecurity and mental health

Housing insecurity has been consistently linked to adverse mental health outcomes across a number of studies [45, 52, 63, 64, 67, 77, 78, 80, 81, 84, 88, 92, 94, 96, 98, 99]. Unstable housing has been shown to be significantly associated with impaired global mental health functioning and greater severity of anxiety among refugees and asylum seekers who have survived torture in the United States [63, 64]. Studies focused on migrants and refugees in Europe have also reported an association between insecure housing and increased incidence of mental health conditions, including psychosis and psychological distress [52, 67]. Similarly, studies focused on Somali migrants and refugees demonstrated that forced moves worsened PTSD symptoms, and residential instability and frequent moves were associated with mental health issues such as anxiety and distress [45, 96]. Several Australian studies have highlighted challenges in securing housing as a significant source of stress among refugee populations, with experiences of discrimination in the housing market and instability in housing tenure in the private rental sector perceived as causing anxiety and depression, and negatively impacting mental health during resettlement [77, 80, 94, 98]. Similar findings were also reported among migrants and refugees in other high-income countries, where housing insecurity stemming from inadequate and insecure living conditions was consistently associated with increased stress, anxiety, and exacerbation of mental health conditions, compounding the challenges of integration and marginalisation [78, 81, 88, 92]. A Canadian study among refugee youth experiencing homelessness identified housing insecurity as a major challenge, with experiences of fear in the emergency shelter and difficulty adjusting to shared accommodations; however, housing interventions such as rental assistance were perceived as beneficial for improving mental wellbeing and facilitating the transition to independent living [84].

#### Neighbourhood conditions and mental health

Neighbourhood conditions were found to be associated with mental health outcomes in several studies [55, 60, 67, 74, 98, 99]. Studies conducted among migrants and refugees in European countries reported that residing in deprived neighbourhoods was associated with higher rates of depression and anxiety, as well as an increased incidence of first episode psychosis compared to their counterparts [55, 60, 67]. Similar findings were reported by Australian studies, which demonstrated that refugee populations living in densely populated suburbs led to social isolation and experiences of depression, while safety concerns in neighbourhoods contributed to increased fear and anxiety, prompting relocation to safer areas to improve wellbeing and ontological security [74, 98, 99].

#### Housing affordability and mental health

A significant association between housing affordability and mental health outcomes was evident among migrant and refugee populations [53, 74, 86, 87, 93, 98, 99]. Housing affordability has been shown to negatively impact the living conditions of older migrant women in the UK, leading to distress due to their lack of control over housing [93]. Similarly, a study conducted among refugees in the United States identified the lack of sufficient income for adequate housing as a major source of distress related to social isolation [86]. Multiple studies conducted among migrants and refugees in Australia have highlighted the impact of housing affordability on mental health [53, 74, 87, 98]. Housing affordability challenges were associated with experiences of depression, while high housing costs were perceived to contribute to financial strain, adversely impacting the mental wellbeing of refugee populations [74, 98]. Among resettled migrants, owning a home was perceived as a significant achievement, yet the burden of mortgage responsibilities led to considerable psychological distress [87]. Moreover, while the impact of housing affordability on mental health was found to be comparable between humanitarian migrants and the wider Australian population, unsuitable housing had a more pronounced adverse effect on the mental health of humanitarian migrants [53].

#### Housing satisfaction and mental health

Housing satisfaction emerged as a significant factor influencing mental health outcomes [35, 41]. Refugees in Canada who were not satisfied with their housing were found to experience a higher prevalence of depression at both baseline and two-year follow-up, compared to those who were satisfied with their housing [35]. Similarly, decreased satisfaction with accommodation was associated with poorer emotional wellbeing among new refugees in the UK [41].

### Assessment of methodological quality

The summary of the quality assessment of the included studies using the JBI critical appraisal tools is presented in Supplementary Table S5. Among the quantitative studies, the 10 cohort studies had 64–91% of the quality items clearly met [35, 37, 41, 44–47, 53, 60, 71], with five studies having one to three items unclear [35, 41, 45, 60, 71]. The main methodological items frequently not met or unclear in the cohort studies included ensuring that participants were free of the outcome at the start of the study (or at the moment of exposure), describing and exploring reasons for loss to follow up, and employing strategies to address incomplete follow up. For the 28 cross-sectional studies, 24 studies had 100% of the quality items clearly met [38–40, 42, 43, 48, 51, 52, 54–59, 61–70], and four studies had at least 75% of the quality items clearly met [36, 49, 50, 72], with three studies having one to two items unclear [36, 50, 72]. The main methodological item frequently not met or unclear in the cross-sectional studies was the description of strategies to deal with confounding factors. Among the 27 qualitative studies, six studies had 100% of the quality items clearly met [78, 81, 83, 88, 89, 99], and 21 studies had at least 70% of the quality items clearly met [73–77, 79, 80, 82, 84–87, 90–98], with nine studies having one to three items unclear [73, 77, 79, 80, 85, 86, 90, 93, 95]. The main methodological items not consistently met or unclear in the qualitative studies included providing a statement locating the researcher culturally or theoretically, addressing the influence of the researcher on the research and vice-versa, and ensuring the research was ethical or providing evidence of ethical approval. Overall, 63 (96.9%) studies in this review had a low risk of bias (scoring ≥ 70%) [36–44, 46–99], only two cohort studies had a moderate risk of bias (scoring 50–69%) [35, 45], and none had a high risk of bias (scoring 0–49%).

## Discussion

### Summary of key findings

This mixed-methods systematic review synthesised the evidence on the association between housing inequalities and health outcomes among migrant and refugee populations in high-income countries. The findings demonstrated a consistent association between aspects of housing and both physical and mental health outcomes, including housing conditions, housing tenure, housing insecurity, and neighbourhood conditions. Other aspects of housing, such as housing affordability and housing satisfaction, were associated with mental health outcomes. Housing inequalities, characterised by disparities in access to adequate, affordable and secure housing, were shown to contribute to a range of physical and mental health issues. Overall, these findings align with existing literature on the critical role of housing as a key social determinant of health [20, 100], and underscore the need to address housing inequalities to improve the health and wellbeing of migrant and refugee populations.

### Pathways linking housing inequalities and health outcomes

The evidence from this systematic review sheds light on the diverse potential pathways through which housing inequalities impact physical and mental health outcomes for migrant and refugee populations. One key pathway identified was from poor housing conditions to negative physical and mental health outcomes, for instance, overcrowding and inadequate ventilation leading to respiratory illnesses as well as experiences of anxiety and depression [51, 58, 82, 95]. This aligns with the findings of prior systematic reviews among refugee and asylum-seeking populations, where poor living conditions in refugee camps as well as resettlement countries negatively impacted physical and mental health [24, 25]. This finding also concurs with a systematic review of housing and health inequalities among the general population in OECD counties, which also identified internal housing conditions as an essential pathway linking housing and health. This review also examined interventions aimed at addressing this pathway and found strong evidence of positive health impacts of warmth and energy efficiency interventions, which could potentially address the issues of cold, dampness and mould also evident in many studies reviewed in this paper [101].

Another key pathway identified was the impact of housing tenure on both physical and mental health, with certain types of housing tenure, such as residing in inadequate rental housing and prolonged stays in asylum centres, being associated with poor health outcomes. For instance, migrants and refugees living in poorly maintained rental housing had higher odds of reporting poor self-rated health and an increased risk of mental health issues compared to those living in self-owned homes [65, 66]. This finding is consistent with a review of the general population of tenants’ responses to housing quality problems in Australia, England, New Zealand and the United States, where poor quality housing was more prevalent in the rental sector than in owner-occupied housing, placing tenants at greater risk of injury and poor respiratory, cardiovascular and mental health [102]. Migrants and refugees are more likely to find themselves living in inadequate rental housing compared to the general population, often due to additional barriers they face apart from low socioeconomic status, such as discrimination, legal restrictions, limited access to financial resources, and exclusion from homeownership opportunities, which further compound their vulnerability to poor health outcomes [12, 13, 103]. Moreover, the impact of housing tenure on health outcomes is particularly pronounced for refugee and migrant populations due to the diverse and often precarious types of housing tenure experienced by those recently arrived, such as asylum centres, refugee facilities, and temporary accommodations. For example, studies included in this review have shown that living in inadequate asylum centres marked by poor living conditions significantly deteriorated the health and wellbeing of refugee populations [85, 90]. Similarly, other types of housing tenure such as refugee housing facilities, as well as short-term and temporary housing, led to poor mental health outcomes [46, 47, 69, 70]. Additionally, the intersection of migration status, socioeconomic conditions, and gender roles can amplify the health risks associated with insecure housing tenure, particularly for migrant women as highlighted by studies in this review [47, 78], who often navigate insecure housing tenures while balancing caregiving responsibilities, further increasing their vulnerability to poor mental and physical health outcomes. This was especially evident in cases where migrant women in short-term lease housing had a higher likelihood of PTSD compared to men in similar housing arrangements, and migrant women facing housing insecurity reporting that their unstable housing situations significantly exacerbated their mental health issues, further underlining the disproportionate impact of insecure housing tenure on their wellbeing [47, 78]. Overall, the ubiquity of these findings on pathways linking housing tenure and physical and mental health highlights the urgent need for reforms by policymakers and housing authorities to address systemic barriers, such as discrimination in housing markets and legal restrictions on housing access for migrant and refugee populations, particularly as the burden of substandard housing disproportionately falls on already disadvantaged populations [104].

The pathway from housing insecurity to negative physical and mental health outcomes for migrant and refugee populations also emerged in this review. Unstable housing situations and insecure tenancy were shown to lead to increased stress and anxiety, as well as exacerbation of both mental and physical health issues [78, 88, 92]. This pathway aligns with the psychosocial stress model, which suggests that chronic stressors, such as housing insecurity, can have profound effects on health and psychological wellbeing [105–107]. Again, this experience is intensified for migrants and refugee populations, who face additional challenges in securing safe and stable housing due to marginalisation and forced displacement. In some cases, housing insecurity leads to homelessness at some point in the resettlement process [108]. The adverse impacts of housing insecurity are often heightened for women within migrant and refugee groups due to the interplay of structural discrimination, caregiving responsibilities, and limited access to resources, demonstrating the compounded nature of housing insecurity when viewed through an intersectional lens [80, 91]. Therefore, addressing housing insecurity through policies that provide more stable housing options and protections for tenants is essential to mitigate the health disparities. Secure housing tenure can also provide stability, greater autonomy, and a sense of control and safety, potentially buffering against stress and associated negative health effects [109].

The pathway from poor neighbourhood conditions to adverse health outcomes was evident in this review, with safety concerns and living in deprived neighbourhoods negatively impacting both physical and mental health [60, 76, 79, 98]. Feelings of safety are particularly critical among individuals from refugee and asylum-seeking backgrounds given potential experiences of trauma, as is feelings of ongoing uncertainty in resettlement countries. It is therefore important to ensure that initial housing placements for the recently arrived are in safe neighbourhoods [98]. Contrary to this recommendation, evidence suggests that recently arrived migrant and refugee populations are often directed to deprived neighbourhoods in order to access cheaper rental housing and existing social and community support networks [16]. However, given that cumulative exposure to such disadvantages can lead to poorer health outcomes even later in life, there is a need for policies aimed at improving neighbourhood conditions, such as investments in infrastructure, community safety programs, and access to green spaces, which could be implemented by local governments in collaboration with international organisations and housing authorities to mitigate long-term health risks from the point of resettlement [16, 110].

Essential pathways linking housing inequalities and mental health that emerged in this review include housing affordability challenges and decreased housing satisfaction leading to poor mental health outcomes, such as experiences of depression and psychological distress [35, 87, 98]. These pathways have also been observed in prior reviews among refugee and asylum-seeking populations, and suggest that the economic burden of housing costs increases stress and pressure and compels them to reside in cheaper and smaller housing, which further contributes to dissatisfaction with living conditions, and significantly impacts mental wellbeing [24, 25]. Moreover, the lack of control over housing situations due to affordability challenges was identified as a source of distress, which corroborates the findings of a prior review that indicated that a lack of control can lead to disempowerment and subsequently impact mental health [25]. Access to adequate and affordable housing is critical as it represents an essential first step in the resettlement process, and initiatives that improve housing affordability and increase levels of housing and neighbourhood satisfaction may serve as a significant investment in the mental health and wellbeing of migrant and refugee populations [111].

### Strengths and limitations

A key strength of this systematic review is the comprehensive and systematic approach to synthesising evidence from both quantitative and qualitative studies, providing a holistic understanding of the association between housing inequalities and health outcomes among migrant and refugee populations. Moreover, the use of multiple electronic databases and grey literature sources ensures a broad and diverse evidence base [112]. Adherence to the JBI methodology for mixed-methods systematic reviews and PRISMA guidelines ensures methodological rigor and transparency, while the registration of the protocol at PROSPERO and subsequent publication of the protocol minimises reporting bias [26, 27].

However, there are certain limitations and methodological considerations to take into account when interpreting the findings of this systematic review. As the inclusion criteria were limited to studies published in English language, relevant research published in other languages may have been overlooked, potentially introducing language bias [113]. There is also the potential of publication bias as studies with significant or positive results are more likely to be published, which may bias observed results [112]. Although grey literature searches were conducted in the selected databases to minimise publication bias, relevant unpublished studies may still have been missed. The assessment of methodological quality showed several shortcomings of the included studies, which could introduce bias and affect the validity of findings in identified papers. Several cohort studies did not clearly ensure that participants were free of the outcome at the start of the study, adequately describe reasons for loss to follow up, or employ strategies to address incomplete follow up. Among the cross-sectional studies, while the majority met all quality criteria, some failed to adequately describe strategies to deal with potential confounding factors, which could influence the observed associations. In the qualitative studies, recurring issues included the lack of statements locating the researcher culturally or theoretically, insufficient addressing of the researcher’s influence on the research, and the absence of evidence of ethical approval. Overall, the variability in study design, research aims, methodological approaches, and study quality of the included studies introduces heterogeneity, limiting the comparability, interpretation and generalisability of the findings [25]. A meta-analysis could not be conducted due to the heterogeneity of migrant and refugee populations and the varying measures of housing quality and health outcomes assessed across studies.

### Implications for practice, policy, and research

The complex and multifaceted pathways through which housing inequalities impact the health outcomes, as identified in this review, highlight the need for targeted housing and public health interventions to improve the health outcomes of migrant and refugee populations [28, 114]. Structural barriers, such as inequitable housing policies, exclusion from public housing programs, and systemic discrimination, further exacerbate housing inequalities and contribute to health disparities within these communities [108, 115]. Addressing these challenges requires the development of comprehensive and supportive housing policies that prioritise the provision of adequate, affordable, and secure housing. This is particularly urgent in the context of the current housing crisis in several high-income countries, such Australia and the United States, where rising rents and insufficient affordable housing exacerbate health disparities among socioeconomically disadvantaged and ethnic minority groups [99, 116]. Adopting a “Health in All Policies” approach to housing policy could be instrumental in addressing these challenges, encouraging leaders in the housing sector to systematically incorporate considerations of health equity into their decision-making processes [117]. For instance, the Housing First model, which prioritises stable housing as a foundation for addressing other social determinants of health, highlights the importance of collaboration between stakeholders, including government agencies, housing authorities, public health organisations, and support service providers, in ensuring that health equity is embedded within housing policies [117, 118]. There is also a need for broader initiatives and policies aimed at improving neighbourhood conditions, such as increasing access to green spaces, addressing neighbourhood-level deprivation, along with ensuring equitable access to essential services and amenities [110]. Building on the findings of this review, there is a need for future research to further elucidate the causal pathways through which housing inequalities impact health outcomes. Longitudinal studies are essential for establishing temporal relationships, and understanding how housing inequalities affect physical and mental health over time [119]. Additionally, future studies are needed to evaluate the effectiveness of interventions aimed at reducing these housing inequalities, such as rental assistance, housing vouchers, and integrated service models, which could help inform more effective and equitable housing policies and practices, ultimately enhancing the health and wellbeing of migrant and refugee populations.

## Conclusions

The evidence from this mixed-methods systematic review highlights several potential pathways through which housing inequalities impact physical and mental health outcomes for migrant and refugee populations in high-income countries. A key pathway identified was poor housing conditions, including overcrowding and inadequate ventilation, contributing to a range of physical and mental health conditions, such as respiratory illnesses and experiences of anxiety and depression. Another significant pathway was the type of housing tenure impacting both physical and mental health, such as living in inadequate rental housing as opposed to self-owned homes being linked with poor physical health and mental health issues. Housing insecurity stemming from unstable housing situations and insecure tenancy also emerged as an essential pathway leading to the exacerbation of both physical and mental health issues. The pathway from poor neighbourhood conditions to adverse health outcomes was also evident in this review, with factors such as safety concerns and living in deprived neighbourhoods negatively impacting both physical and mental health. Furthermore, essential pathways linking housing affordability challenges and decreased housing satisfaction with poor mental health outcomes such as experiences of depression and psychological distress were also identified. Overall, this review highlights the critical role of housing as a social determinant of health, with significant implications for both physical and mental health for refugee and migrant populations. Addressing housing inequalities through comprehensive policies and interventions that ensure access to adequate, affordable, and secure housing, while also improving neighbourhood conditions, is essential for improving the health and wellbeing of migrant and refugee populations.

## Electronic supplementary material

Below is the link to the electronic supplementary material.

Supplementary Material 1

Supplementary Material 2

Supplementary Material 3

Supplementary Material 4

Supplementary Material 5

## Data Availability

All data generated or analysed during this study are included in this published article and its supplementary information files.
