# Supplementary material for: Housing inequalities and health outcomes among migrant and refugee populations in high-income countries: a mixed-methods systematic review
Source: BMC Public Health. 2025 Mar 22;25:1098. doi: 10.1186/s12889-025-22186-5 (PMC11929249; doi:10.1186/s12889-025-22186-5)
Supplement: Supplementary file 1 — Supplementary Material 1 [file 12889_2025_22186_MOESM1_ESM.docx]

**Supplementary Table S1.** Search strategy for electronic databases conducted in December 2024

1. **Medline (OVID)**

| **#** | **Query** | **Results** |
| --- | --- | --- |
| 1 | (migrant* or immigrant* or emigrant* or asylum seeker* or refugee* or displaced person*).ti,ab,kw. | 71387 |
| 2 | "Transients and Migrants"/ or "Emigrants and Immigrants"/ or Refugees/ | 43006 |
| 3 | 1 or 2 | 81182 |
| 4 | (hous* or accommodation* or living condition* or residence* or refugee camp* or housing inequalit*).ti,ab,kw. | 378166 |
| 5 | Public Housing/ or Housing/ or Refugee Camps/ or Housing Quality/ or Housing Instability/ | 22859 |
| 6 | 4 or 5 | 386801 |
| 7 | (health* or wellbeing or "quality of life").ti,ab,kw. | 4280854 |
| 8 | Health/ or Public Health/ or Health Inequities/ or Health Status/ or Health Equity/ or "Quality of Life"/ | 493979 |
| 9 | 7 or 8 | 4370041 |
| 10 | 3 and 6 and 9 | 5555 |
| 11 | ((high* or upper) adj5 income? adj5 (countr* or econom* or group? or nation?)).mp. | 22295 |
| 12 | (America* or Andorra* or Antigua* or Aruba* or Australia* or Austria* or Barbuda* or Bermuda* or Britain or British or Baham* or Bahrain* or Barbad* or Belgium or Belgian* or Brunei* or Canada or Canadian* or Cayman Island* or Channel Island* or Chile* or Croatia* or Curacao* or Cyprus or Cyprian* or Cypriot? or Czech* or Darussalam or Denmark or Danish or England or English or Estonia* or Faroe Island* or Finland or Finnish or Finn? or France or French or German* or Gibralta* or Greece or Greek* or Greenland* or Guam* or Hong Kong* or Hungary or Hungarian* or Iceland* or Ireland or Irish or "Isle of Man" or Israel* or Italy or Italian* or Japan* or South Korea* or Kuwait* or Latvia* or Liechtenstein* or Lithuania* or Luxembourg* or Macao* or Malta or Maltese or Monaco or Nauru* or Netherlands or Dutch or New Caledonia* or New Zealand* or Northern Mariana Island* or Norway or Norwegian* or Oman* or Panama* or Poland or Polish or Portug* or Puerto Ric* or Romania* or Qatar* or Saint Kitts or San Marino or Saint Martin or Sint Maarten or Saudi Arabia* or Seychelles or Singapore* or Slovak* or Slovenia* or Spain or Spanish or Sweden or Swedish or Switzerland or Swiss or Taiwan* or Trinidad* or Tobago* or (Turks and Caicos Island*) or United Arab Emirates or United Kingdom or UK or United States or USA or Uruguay* or Virgin Island* or (western adj (countr* or econom* or nation*))).mp. | 6258820 |
| 13 | (America* or Andorra* or Antigua* or Aruba* or Australia* or Austria* or Barbuda* or Bermuda* or Britain or British or Baham* or Bahrain* or Barbad* or Belgium or Belgian* or Brunei* or Canada or Canadian* or Cayman Island* or Channel Island* or Chile* or Croatia* or Curacao* or Cyprus or Cyprian* or Cypriot? or Czech* or Darussalam or Denmark or Danish or England or English or Estonia* or Faroe Island* or Finland or Finnish or Finn? or France or French or German* or Gibralta* or Greece or Greek* or Greenland* or Guam* or Hong Kong* or Hungary or Hungarian* or Iceland* or Ireland or Irish or "Isle of Man" or Israel* or Italy or Italian* or Japan* or South Korea* or Kuwait* or Latvia* or Liechtenstein* or Lithuania* or Luxembourg* or Macao* or Malta or Maltese or Monaco or Nauru* or Netherlands or Dutch or New Caledonia* or New Zealand* or Northern Mariana Island* or Norway or Norwegian* or Oman* or Panama* or Poland or Polish or Portug* or Puerto Ric* or Romania* or Qatar* or Saint Kitts or San Marino or Saint Martin or Sint Maarten or Saudi Arabia* or Seychelles or Singapore* or Slovak* or Slovenia* or Spain or Spanish or Sweden or Swedish or Switzerland or Swiss or Taiwan* or Trinidad* or Tobago* or (Turks and Caicos Island*) or United Arab Emirates or United Kingdom or UK or United States or USA or Uruguay* or Virgin Island* or (western adj (countr* or econom* or nation*))).sh. | 2909111 |
| 14 | 11 or 12 or 13 | 6272488 |
| 15 | 10 and 14 | 3304 |

1. **Web of Science (ISI)**

| **#** | **Query** | **Results** |
| --- | --- | --- |
| 1 | migrant* OR immigrant* OR emigrant* OR "asylum seeker*" OR refugee* OR "displaced person*" (Topic) | 240299 |
| 2 | hous* OR accommodation* OR "living condition*" OR residence* OR "refugee camp*" OR "housing inequalit*" OR "housing qualit*" (Topic) | 945451 |
| 3 | health* OR wellbeing OR "quality of life" (Topic) | 5592385 |
| 4 | #1 AND #2 AND #3 | 8083 |
| 5 | ((high* OR upper ) NEAR/5 income* NEAR/5 (countr* OR econom* OR group* OR nation* )) (Topic) | 30868 |
| 6 | (western NEAR/1 ( countr* OR econom* OR nation*)) (Topic) | 37867 |
| 7 | america* OR andorra* OR antigua* OR aruba* OR australia* OR austria* OR barbuda* OR bermuda* OR britain OR british OR baham* OR bahrain* OR barbad* OR belgium OR belgian* OR brunei* OR canada OR canadian* OR "Cayman Island*" OR "Channel Island*" OR chile* OR croatia* OR curacao* OR cyprus OR cyprian* OR cypriot? OR czech* OR darussalam OR denmark OR danish OR england OR english OR estonia* OR "faroe island*" OR finland OR finnish OR finn? OR france OR french OR german* OR gibralta* OR greece OR greek* OR greenland* OR guam* OR "Hong Kong* " OR hungary OR hungarian* OR iceland* OR ireland OR irish OR "Isle of Man" OR israel* OR italy OR italian* OR japan* OR "south korea*" OR kuwait* OR latvia* OR liechtenstein* OR lithuania* OR luxembourg* OR macao* OR malta OR maltese OR monaco OR nauru* OR netherlands OR dutch OR "New Caledonia*" OR "new zealand*" OR "Northern Mariana Island*" OR norway OR norwegian* OR oman* OR panama* OR poland OR polish OR portug* OR "puerto ric*" OR romania* OR qatar* OR "Saint Kitts" OR "San Marino" OR "Saint Martin" OR "Sint Maarten" OR "Saudi Arabia*" OR seychelles OR singapore* OR slovak* OR slovenia* OR spain OR spanish OR sweden OR swedish OR switzerland OR swiss OR taiwan* OR trinidad* OR tobago* OR "Turks and Caicos Island*" OR "United Arab Emirates" OR "United Kingdom" OR uk OR "United States" OR usa OR uruguay* OR "Virgin Island*" (Topic) | 10077995 |
| 8 | #5 OR #6 OR #7 | 10116493 |
| 9 | #4 AND #8 | 4450 |

1. **Embase (OVID)**

| **#** | **Query** | **Results** |
| --- | --- | --- |
| 1 | migrant/ or emigrant/ or forced migrant/ or immigrant/ or migrant worker/ | 38180 |
| 2 | refugee/ or asylum seeker/ | 19756 |
| 3 | (migrant* or immigrant* or emigrant* or asylum seeker* or refugee* or displaced person*).ti,ab,kw. | 81061 |
| 4 | 1 or 2 or 3 | 90550 |
| 5 | housing/ | 36485 |
| 6 | refugee camp/ | 1290 |
| 7 | housing quality/ | 826 |
| 8 | housing instability/ | 967 |
| 9 | (hous* or accommodation* or living condition* or residence* or refugee camp* or housing inequalit*).ti,ab,kw. | 506544 |
| 10 | 5 or 6 or 7 or 8 or 9 | 516670 |
| 11 | health/ | 254046 |
| 12 | public health/ | 265791 |
| 13 | health disparity/ | 45416 |
| 14 | health status/ | 163277 |
| 15 | health equity/ | 14441 |
| 16 | "quality of life"/ | 703710 |
| 17 | (health* or wellbeing or "quality of life").ti,ab,kw. | 5776179 |
| 18 | 11 or 12 or 13 or 14 or 15 or 16 or 17 | 6086028 |
| 19 | ((high* or upper) adj5 income? adj5 (countr* or econom* or group? or nation?)).mp. | 34969 |
| 20 | (America* or Andorra* or Antigua* or Aruba* or Australia* or Austria* or Barbuda* or Bermuda* or Britain or British or Baham* or Bahrain* or Barbad* or Belgium or Belgian* or Brunei* or Canada or Canadian* or Cayman Island* or Channel Island* or Chile* or Croatia* or Curacao* or Cyprus or Cyprian* or Cypriot? or Czech* or Darussalam or Denmark or Danish or England or English or Estonia* or Faroe Island* or Finland or Finnish or Finn? or France or French or German* or Gibralta* or Greece or Greek* or Greenland* or Guam* or Hong Kong* or Hungary or Hungarian* or Iceland* or Ireland or Irish or "Isle of Man" or Israel* or Italy or Italian* or Japan* or South Korea* or Kuwait* or Latvia* or Liechtenstein* or Lithuania* or Luxembourg* or Macao* or Malta or Maltese or Monaco or Nauru* or Netherlands or Dutch or New Caledonia* or New Zealand* or Northern Mariana Island* or Norway or Norwegian* or Oman* or Panama* or Poland or Polish or Portug* or Puerto Ric* or Romania* or Qatar* or Saint Kitts or San Marino or Saint Martin or Sint Maarten or Saudi Arabia* or Seychelles or Singapore* or Slovak* or Slovenia* or Spain or Spanish or Sweden or Swedish or Switzerland or Swiss or Taiwan* or Trinidad* or Tobago* or (Turks and Caicos Island*) or United Arab Emirates or United Kingdom or UK or United States or USA or Uruguay* or Virgin Island* or (western adj (countr* or econom* or nation*))).mp. | 9074817 |
| 21 | (America* or Andorra* or Antigua* or Aruba* or Australia* or Austria* or Barbuda* or Bermuda* or Britain or British or Baham* or Bahrain* or Barbad* or Belgium or Belgian* or Brunei* or Canada or Canadian* or Cayman Island* or Channel Island* or Chile* or Croatia* or Curacao* or Cyprus or Cyprian* or Cypriot? or Czech* or Darussalam or Denmark or Danish or England or English or Estonia* or Faroe Island* or Finland or Finnish or Finn? or France or French or German* or Gibralta* or Greece or Greek* or Greenland* or Guam* or Hong Kong* or Hungary or Hungarian* or Iceland* or Ireland or Irish or "Isle of Man" or Israel* or Italy or Italian* or Japan* or South Korea* or Kuwait* or Latvia* or Liechtenstein* or Lithuania* or Luxembourg* or Macao* or Malta or Maltese or Monaco or Nauru* or Netherlands or Dutch or New Caledonia* or New Zealand* or Northern Mariana Island* or Norway or Norwegian* or Oman* or Panama* or Poland or Polish or Portug* or Puerto Ric* or Romania* or Qatar* or Saint Kitts or San Marino or Saint Martin or Sint Maarten or Saudi Arabia* or Seychelles or Singapore* or Slovak* or Slovenia* or Spain or Spanish or Sweden or Swedish or Switzerland or Swiss or Taiwan* or Trinidad* or Tobago* or (Turks and Caicos Island*) or United Arab Emirates or United Kingdom or UK or United States or USA or Uruguay* or Virgin Island* or (western adj (countr* or econom* or nation*))).sh. | 4257891 |
| 22 | 19 or 20 or 21 | 9095899 |
| 23 | 4 and 10 and 18 and 22 | 3772 |

1. **PsycInfo (Ovid)**

| **#** | **Query** | **Results** |
| --- | --- | --- |
| 1 | migrant workers/ or foreign workers/ or immigration/ | 29841 |
| 2 | refugees/ or asylum seeking/ | 10321 |
| 3 | (migrant* or immigrant* or emigrant* or asylum seeker* or refugee* or displaced person*).mp. | 58242 |
| 4 | 1 or 2 or 3 | 61737 |
| 5 | housing/ or shelters/ | 8935 |
| 6 | (hous* or accommodation* or living condition* or residence* or refugee camp* or housing inequalit*).ti,ab. | 118493 |
| 7 | 5 or 6 | 120428 |
| 8 | health/ | 55364 |
| 9 | public health/ | 30432 |
| 10 | health disparities/ | 12928 |
| 11 | health status/ | 2384 |
| 12 | "quality of life"/ | 51510 |
| 13 | (health* or wellbeing or "quality of life").ti,ab. | 1002115 |
| 14 | 8 or 9 or 10 or 11 or 12 or 13 | 1012705 |
| 15 | ((high* or upper) adj5 income? adj5 (countr* or econom* or group? or nation?)).mp. | 4615 |
| 16 | (America* or Andorra* or Antigua* or Aruba* or Australia* or Austria* or Barbuda* or Bermuda* or Britain or British or Baham* or Bahrain* or Barbad* or Belgium or Belgian* or Brunei* or Canada or Canadian* or Cayman Island* or Channel Island* or Chile* or Croatia* or Curacao* or Cyprus or Cyprian* or Cypriot? or Czech* or Darussalam or Denmark or Danish or England or English or Estonia* or Faroe Island* or Finland or Finnish or Finn? or France or French or German* or Gibralta* or Greece or Greek* or Greenland* or Guam* or Hong Kong* or Hungary or Hungarian* or Iceland* or Ireland or Irish or "Isle of Man" or Israel* or Italy or Italian* or Japan* or South Korea* or Kuwait* or Latvia* or Liechtenstein* or Lithuania* or Luxembourg* or Macao* or Malta or Maltese or Monaco or Nauru* or Netherlands or Dutch or New Caledonia* or New Zealand* or Northern Mariana Island* or Norway or Norwegian* or Oman* or Panama* or Poland or Polish or Portug* or Puerto Ric* or Romania* or Qatar* or Saint Kitts or San Marino or Saint Martin or Sint Maarten or Saudi Arabia* or Seychelles or Singapore* or Slovak* or Slovenia* or Spain or Spanish or Sweden or Swedish or Switzerland or Swiss or Taiwan* or Trinidad* or Tobago* or (Turks and Caicos Island*) or United Arab Emirates or United Kingdom or UK or United States or USA or Uruguay* or Virgin Island* or (western adj (countr* or econom* or nation*))).mp. | 1239542 |
| 17 | (America* or Andorra* or Antigua* or Aruba* or Australia* or Austria* or Barbuda* or Bermuda* or Britain or British or Baham* or Bahrain* or Barbad* or Belgium or Belgian* or Brunei* or Canada or Canadian* or Cayman Island* or Channel Island* or Chile* or Croatia* or Curacao* or Cyprus or Cyprian* or Cypriot? or Czech* or Darussalam or Denmark or Danish or England or English or Estonia* or Faroe Island* or Finland or Finnish or Finn? or France or French or German* or Gibralta* or Greece or Greek* or Greenland* or Guam* or Hong Kong* or Hungary or Hungarian* or Iceland* or Ireland or Irish or "Isle of Man" or Israel* or Italy or Italian* or Japan* or South Korea* or Kuwait* or Latvia* or Liechtenstein* or Lithuania* or Luxembourg* or Macao* or Malta or Maltese or Monaco or Nauru* or Netherlands or Dutch or New Caledonia* or New Zealand* or Northern Mariana Island* or Norway or Norwegian* or Oman* or Panama* or Poland or Polish or Portug* or Puerto Ric* or Romania* or Qatar* or Saint Kitts or San Marino or Saint Martin or Sint Maarten or Saudi Arabia* or Seychelles or Singapore* or Slovak* or Slovenia* or Spain or Spanish or Sweden or Swedish or Switzerland or Swiss or Taiwan* or Trinidad* or Tobago* or (Turks and Caicos Island*) or United Arab Emirates or United Kingdom or UK or United States or USA or Uruguay* or Virgin Island* or (western adj (countr* or econom* or nation*))).sh. | 13149 |
| 18 | 15 or 16 or 17 | 1242394 |
| 19 | 4 and 7 and 14 and 18 | 1448 |

1. **Scopus**

6485 documents

( ( TITLE-ABS-KEY ( migrant* OR immigrant* OR emigrant* OR "asylum seeker*" OR refugee* OR "displaced person*" ) ) AND ( TITLE-ABS-KEY ( hous* OR accommodation* OR "living condition*" OR residence* OR "refugee camp*" OR "housing inequalit*" OR "housing qualit*" ) ) AND ( TITLE-ABS-KEY ( health* OR wellbeing OR "quality of life" ) ) ) AND ( ( TITLE-ABS-KEY ( ( ( ( high* OR upper ) W/5 income* W/5 ( countr* OR econom* OR group* OR nation* ) ) ) ) ) OR ( TITLE-ABS-KEY ( ( western W/1 ( countr* OR econom* OR nation* ) ) ) ) OR ( TITLE-ABS-KEY ( america* OR andorra* OR antigua* OR aruba* OR australia* OR austria* OR barbuda* OR bermuda* OR britain OR british OR baham* OR bahrain* OR barbad* OR belgium OR belgian* OR brunei* OR canada OR canadian* OR "Cayman Island*" OR "Channel Island*" OR chile* OR croatia* OR curacao* OR cyprus OR cyprian* OR cypriot? OR czech* OR darussalam OR denmark OR danish OR england OR english OR estonia* OR "faroe island*" OR finland OR finnish OR finn? OR france OR french OR german* OR gibralta* OR greece OR greek* OR greenland* OR guam* OR "Hong Kong* " OR hungary OR hungarian* OR iceland* OR ireland OR irish OR "Isle of Man" OR israel* OR italy OR italian* OR japan* OR "south korea*" OR kuwait* OR latvia* OR liechtenstein* OR lithuania* OR luxembourg* OR macao* OR malta OR maltese OR monaco OR nauru* OR netherlands OR dutch OR "New Caledonia*" OR "new zealand*" OR "Northern Mariana Island*" OR norway OR norwegian* OR oman* OR panama* OR poland OR polish OR portug* OR "puerto ric*" OR romania* OR qatar* OR "Saint Kitts" OR "San Marino" OR "Saint Martin" OR "Sint Maarten" OR "Saudi Arabia*" OR seychelles OR singapore* OR slovak* OR slovenia* OR spain OR spanish OR sweden OR swedish OR switzerland OR swiss OR taiwan* OR trinidad* OR tobago* OR "Turks and Caicos Island*" OR "United Arab Emirates" OR "United Kingdom" OR uk OR "United States" OR usa OR uruguay* OR "Virgin Island*" ) ) )

1. **CINAHL (EBSCO)**

| **#** | **Query** | **Results** |
| --- | --- | --- |
| S1 | (MH "Migrants") | 6529 |
| S2 | (MH "Immigrants+") | 19313 |
| S3 | (MH "Refugees+") | 10033 |
| S4 | migrant* or immigrant* or emigrant* or "asylum seeker*" or refugee* or "displaced person*" | 43556 |
| S5 | S1 OR S2 OR S3 OR S4 | 43606 |
| S6 | (MH "Housing+") OR (MH "Public Housing") | 16620 |
| S7 | (MH "Refugee Camps") | 270 |
| S8 | (MH "Housing Instability") | 379 |
| S9 | hous* or accommodation* or "living condition*" or residence* or "refugee camp*" or "housing inequalit*" | 128119 |
| S10 | S6 OR S7 OR S8 OR S9 | 128750 |
| S11 | (MH "Health+") | 491505 |
| S12 | (MH "Public Health+") | 1504735 |
| S13 | (MH "Health Inequities") | 3309 |
| S14 | (MH "Health Status+") OR (MH "Health Status Disparities") | 150933 |
| S15 | (MH "Quality of Life+") OR (MH "Well-Being (Iowa NOC)") | 163587 |
| S16 | (MH "Health and Life Quality (Iowa NOC)+") | 5 |
| S17 | TI ( health* or wellbeing or "quality of life" ) OR AB ( health* or wellbeing or "quality of life" ) | 1615199 |
| S18 | S11 OR S12 OR S13 OR S14 OR S15 OR S16 OR S17 | 2820234 |
| S19 | ((high* or upper) N5 income* N5 (countr* or econom* or group? or nation?)) | 8483 |
| S20 | (America* or Andorra* or Antigua* or Aruba* or Australia* or Austria* or Barbuda* or Bermuda* or Britain or British or Baham* or Bahrain* or Barbad* or Belgium or Belgian* or Brunei* or Canada or Canadian* or "Cayman Island*" or "Channel Island*" or Chile* or Croatia* or Curacao* or Cyprus or Cyprian* or Cypriot? or Czech* or Darussalam or Denmark or Danish or England or English or Estonia* or "Faroe Island*" or Finland or Finnish or Finn? or France or French or German* or Gibralta* or Greece or Greek* or Greenland* or Guam* or "Hong Kong*" or Hungary or Hungarian* or Iceland* or Ireland or Irish or "Isle of Man" or Israel* or Italy or Italian* or Japan* or South Korea* or Kuwait* or Latvia* or Liechtenstein* or Lithuania* or Luxembourg* or Macao* or Malta or Maltese or Monaco or Nauru* or Netherlands or Dutch or "New Caledonia*" or "New Zealand*" or "Northern Mariana Island*" or Norway or Norwegian* or Oman* or Panama* or Poland or Polish or Portug* or Puerto Ric* or Romania* or Qatar* or "Saint Kitts" or "San Marino" or "Saint Martin" or "Sint Maarten" or "Saudi Arabia*" or Seychelles or Singapore* or Slovak* or Slovenia* or Spain or Spanish or Sweden or Swedish or Switzerland or Swiss or Taiwan* or Trinidad* or Tobago* or (Turks and "Caicos Island*") or "United Arab Emirates" or "United Kingdom" or UK or "United States" or USA or Uruguay* or "Virgin Island*") or (western N2 (countr* or econom* or nation*)) | 867595 |
| S21 | MW (America* or Andorra* or Antigua* or Aruba* or Australia* or Austria* or Barbuda* or Bermuda* or Britain or British or Baham* or Bahrain* or Barbad* or Belgium or Belgian* or Brunei* or Canada or Canadian* or "Cayman Island*" or "Channel Island*" or Chile* or Croatia* or Curacao* or Cyprus or Cyprian* or Cypriot? or Czech* or Darussalam or Denmark or Danish or England or English or Estonia* or "Faroe Island*" or Finland or Finnish or Finn? or France or French or German* or Gibralta* or Greece or Greek* or Greenland* or Guam* or "Hong Kong*" or Hungary or Hungarian* or Iceland* or Ireland or Irish or "Isle of Man" or Israel* or Italy or Italian* or Japan* or South Korea* or Kuwait* or Latvia* or Liechtenstein* or Lithuania* or Luxembourg* or Macao* or Malta or Maltese or Monaco or Nauru* or Netherlands or Dutch or "New Caledonia*" or "New Zealand*" or "Northern Mariana Island*" or Norway or Norwegian* or Oman* or Panama* or Poland or Polish or Portug* or Puerto Ric* or Romania* or Qatar* or "Saint Kitts" or "San Marino" or "Saint Martin" or "Sint Maarten" or "Saudi Arabia*" or Seychelles or Singapore* or Slovak* or Slovenia* or Spain or Spanish or Sweden or Swedish or Switzerland or Swiss or Taiwan* or Trinidad* or Tobago* or (Turks and "Caicos Island*") or "United Arab Emirates" or "United Kingdom" or UK or "United States" or USA or Uruguay* or "Virgin Island*" or (western N2 (countr* or econom* or nation*)) | 614608 |
| S22 | S19 OR S20 OR S21 | 874279 |
| S23 | S5 AND S10 AND S18 AND S22 | 1214 |
