# Supplementary material for: Housing inequalities and health outcomes among migrant and refugee populations in high-income countries: a mixed-methods systematic review
Source: BMC Public Health. 2025 Mar 22;25:1098. doi: 10.1186/s12889-025-22186-5 (PMC11929249; doi:10.1186/s12889-025-22186-5)
Supplement: Supplementary file 2 — Supplementary Material 2 [file 12889_2025_22186_MOESM2_ESM.docx]

**Supplementary Table S2.** List of excluded studies with reasons

| **Reason for exclusion** | **List of studies excluded** |
| --- | --- |
| Did not meet eligibility based on study design | [1-11] |
| Did not meet eligibility criteria for outcome | [12-28] |
| Did not meet eligibility criteria for population and setting | [29-36] |
| Did not meet eligibility criteria for country of study | [37-44] |
| Did not meet eligibility criteria for phenomenon of interest | [45-70] |

**References:**

1. Fozdar F, Hartley L. Refugee Resettlement in Australia: What We Know and Need to Know. Refugee Survey Quarterly. 2013;32(3):23-51.

2. Bohnet H, Rüegger S. Refugees and Covid-19: Beyond Health Risks to Insecurity. Schweiz Z Polit. 2021;27(2):353-68.

3. Lynch MA. Providing health care for refugee children and unaccompanied minors. Med Confl Surviv. 2001;17(2):125-30.

4. Mahase E. Asylum seekers' health suffers from "unsafe" UK accommodation, report claims. BMJ. 2022;377:o1088.

5. Marsh B, Milofsky C, Kissam E, Arcury TA. Understanding the Role of Social Factors in Farmworker Housing and Health. New Solut. 2015;25(3):313-33.

6. Ramirez SM, Villarejo D. Poverty, housing, and the rural slum: policies and the production of inequities, past and present. Am J Public Health. 2012;102(9):1664-75.

7. Schneider B. Providing for the health needs of migrant children. Nurse Pract. 1986;11(2):54-8, 60, 5.

8. Toosi A, Richter S, Woytowich B. Theoretical Perspectives on Issues and Interventions Related to Migrant Resettlement in Canada. J Transcult Nurs. 2017;28(3):230-5.

9. Clarke SK, Kumar GS, Sutton J, Atem J, Banerji A, Brindamour M, et al. Potential Impact of COVID-19 on Recently Resettled Refugee Populations in the United States and Canada: Perspectives of Refugee Healthcare Providers. J Immigr Minor Health. 2021;23(1):184-9.

10. Elisabeth M, Maneesh P-S, Katarina SF, Slobodan Z, Michael S. Antimicrobial Resistance & Migrants in Sweden: Poor Living Conditions Enforced by Migration Control Policies as a Risk Factor for Optimal Public Health Management. Frontiers in Public Health. 2021;9.

11. Foliaki S. Migration and mental health: The Tongan experience. International Journal of Mental Health. 1997;26(3):36-54.

12. Cheer T, Kearns R, Murphy L. Housing Policy, Poverty, and Culture: ‘Discounting’ Decisions among Pacific Peoples in Auckland, New Zealand. Environment and Planning C: Government and Policy. 2002;20(4):497-516.

13. Moreira AL, Barbosa M, Maia M, Veiga E, Martins F, Santos M. Lives on hold: the experiences of asylum seekers in Moria refugee camp. Community Psychology in Global Perspective. 2020;6(1):92-107.

14. Quandt SA, Wiggins MF, Chen H, Bischoff WE, Arcury TA. Heat index in migrant farmworker housing: implications for rest and recovery from work-related heat stress. Am J Public Health. 2013;103(8):e24-6.

15. Singh V. Dynamics of affordability and immigration in the Canadian housing market. International Journal of Housing Markets and Analysis. 2022;15(3):709-32.

16. Alvarez AL, Müller-Eie D. Neighbourhood Conditions and Quality of Life Among Local and Immigrant Population in Norway. International Journal of Community Well-Being. 2022;5(4):753-76.

17. Bask M. Increasing Inequality in Social Exclusion Occurrence: The Case of Sweden During 1979–2003. Social Indicators Research. 2010;97(3):299-323.

18. Escobedo LE, Champion WM, Li N, Montoya LD. Indoor air quality in Latino homes in Boulder, Colorado. Atmospheric Environment. 2014;92:69-75.

19. Fozdar F, Hartley L. Housing and the creation of home for refugees in Western Australia. Housing, Theory and Society. 2014;31(2):148-73.

20. Simone D, Newbold KB. Housing Trajectories Across the Urban Hierarchy: Analysis of the Longitudinal Survey of Immigrants to Canada, 2001–2005. Housing Studies. 2014;29(8):1096-116.

21. Tomaszewski W, Perales F. Who settles for less? Subjective dispositions, objective circumstances, and housing satisfaction. Social indicators research. 2014;118:181-203.

22. Alwan RM, Schumacher DJ, Cicek-Okay S, Jernigan S, Beydoun A, Salem T, et al. Beliefs, perceptions, and behaviors impacting healthcare utilization of Syrian refugee children. PLoS One. 2020;15(8):e0237081.

23. Feinberg I, O’Connor MH, Owen-Smith A, Dube SR. Public health crisis in the refugee community: little change in social determinants of health preserve health disparities. Health Education Research. 2021;36(2):170-7.

24. Flatau P, Colic-Peisker V, Bauskis A, Maginn P, Buergelt P. Refugees, housing, and neighbourhoods in Australia. Melbourne, Australia: Australian Housing and Urban Research Institute; 2014.

25. Ewers MC, Diop A, Le KT, Bader L. Migrant Worker Well-Being and Its Determinants: The Case of Qatar. Social Indicators Research. 2020;152(1):137-63.

26. Hauge ÅL, Støa E, Denizou K. Framing outsidedness–aspects of housing quality in decentralized reception centres for asylum seekers in Norway. Housing, Theory and Society. 2017;34(1):1-20.

27. Kim SY, Matsui EC, Wen W, Tse HW, Chambliss SE. Demographic and Psychosocial Characteristics, Air Pollution Exposure, and Housing Mobility of Mexican Immigrant Families. J Racial Ethn Health Disparities. 2023;10(6):2970-85.

28. Weiler AM, Caxaj CS. Housing, health equity, and global capitalist power: Migrant farmworkers in Canada. Soc Sci Med. 2024;354:117067.

29. Hirani K, Payne DN, Mutch R, Cherian S. Medical needs of adolescent refugees resettling in Western Australia. Arch Dis Child. 2019;104(9):880-3.

30. Kirkman M, Keys D, Bodzak D, Turner A. "Are we moving again this week?" Children's experiences of homelessness in Victoria, Australia. Soc Sci Med. 2010;70(7):994-1001.

31. Monasta L, Andersson N, Ledogar RJ, Cockcroft A. Minority health and small numbers epidemiology: a case study of living conditions and the health of children in 5 foreign Romá camps in Italy. Am J Public Health. 2008;98(11):2035-41.

32. Green MA, Subramanian SV, Vickers D, Dorling D. Internal migration, area effects and health: Does where you move to impact upon your health? Soc Sci Med. 2015;136-137:27-34.

33. Kahlmeier S, Schindler C, Grize L, Braun-Fahrländer C. Perceived environmental housing quality and wellbeing of movers. J Epidemiol Community Health. 2001;55(10):708-15.

34. Grineski SE, Hernández AA. Landlords, fear, and children's respiratory health: an untold story of environmental injustice in the central city. Local Environment. 2010;15(3):199-216.

35. Barmark M. Social determinants of the sick building syndrome: exploring the interrelated effects of social position and psychosocial situation. Int J Environ Health Res. 2015;25(5):490-507.

36. Mohan G, Barlow P. Area-level deprivation, neighbourhood factors and associations with mental health. PLoS One. 2023;18(1):e0281146.

37. Zabaneh JE, Watt GC, O'Donnell CA. Living and health conditions of Palestinian refugees in an unofficial camp in the Lebanon: a cross-sectional survey. J Epidemiol Community Health. 2008;62(2):91-7.

38. Luksamijarulkul P, Suknongbung S, Vatanasomboon P, Sujirarut D. Health Status, Environmental Living Conditions and Microbial Indoor Air Quality among Migrant Worker Households In Thailand. Southeast Asian J Trop Med Public Health. 2017;48(2):396-406.

39. Al-Khatib IA, Arafat RN, Musmar M. Housing environment and women's health in a Palestinian refugee camp. Int J Environ Health Res. 2005;15(3):181-91.

40. Al-Khatib IA, Tabakhna H. Housing conditions and health in Jalazone Refugee Camp in Palestine. East Mediterr Health J. 2006;12(1-2):144-52.

41. Al-Khatib I, Ju'ba A, Kamal N, Hamed N, Hmeidan N, Massad S. Impact of housing conditions on the health of the people at al-Ama'ri refugee camp in the West Bank of Palestine. Int J Environ Health Res. 2003;13(4):315-26.

42. Ellison GTH, De Wet T. Johannesburg's 'poor housing, good health' paradox: the role of health status assessment, statistical modelling, residential context and migrant status. Public Health. 2020;186:257-64.

43. Li J, Liu Z. Housing stress and mental health of migrant populations in urban China. Cities. 2018;81:172-9.

44. Xiao Y, Miao S, Sarkar C, Geng H, Lu Y. Exploring the Impacts of Housing Condition on Migrants' Mental Health in Nanxiang, Shanghai: A Structural Equation Modelling Approach. Int J Environ Res Public Health. 2018;15(2).

45. Llopis Alvarez A, Müller-Eie D. Housing circumstances and quality of life among local and immigrant population in Norwegian neighbourhoods. Journal of Housing and the Built Environment. 2022;37(1):157-78.

46. Carter TS, Polevychok C, Osborne J. The role of housing and neighbourhood in the re-settlement process: a case study of refugee households in Winnipeg. Canadian Geographies / Géographies canadiennes. 2009;53(3):305-22.

47. Arcury TA, Weir MM, Summers P, Chen H, Bailey M, Wiggins MF, et al. Safety, security, hygiene and privacy in migrant farmworker housing. New Solut. 2012;22(2):153-73.

48. Early J, Davis SW, Quandt SA, Rao P, Snively BM, Arcury TA. Housing characteristics of farmworker families in North Carolina. J Immigr Minor Health. 2006;8(2):173-84.

49. Deveci Y. Trying to understand: Promoting the psychosocial well-being of separated refugee children. Journal of Social Work Practice. 2012;26(3):367-83.

50. Fennelly K. Listening to the experts: provider recommendations on the health needs of immigrants and refugees. J Cult Divers. 2006;13(4):190-201.

51. Al-Adhami M, Berglund E, Wångdahl J, Salari R. A cross-sectional study of health and well-being among newly settled refugee migrants in Sweden-The role of health literacy, social support and self-efficacy. PLoS One. 2022;17(12):e0279397.

52. Leiler A, Bjärtå A, Ekdahl J, Wasteson E. Mental health and quality of life among asylum seekers and refugees living in refugee housing facilities in Sweden. Soc Psychiatry Psychiatr Epidemiol. 2019;54(5):543-51.

53. Organista KC, Jung W, Neilands TB. Working and Living Conditions and Psychological Distress in Latino Migrant Day Laborers. Health Educ Behav. 2019;46(4):637-47.

54. Ortega-Alcázar I, Dyck I. Migrant narratives of health and well-being: Challenging ‘othering’ processes through photo-elicitation interviews. Critical Social Policy. 2011;32(1):106-25.

55. Rosen J, Ciudad-Real V, Angst S, Painter G. Rental Affordability, Coping Strategies, and Impacts in Diverse Immigrant Communities. Housing Policy Debate. 2023;33(6):1313-32.

56. Biddle L, Hintermeier M, Mohsenpour A, Sand M, Bozorgmehr K. Monitoring the health and healthcare provision for refugees in collective accommodation centres: Results of the population-based survey RESPOND. J Health Monit. 2021;6(1):7-29.

57. Brabant Z, Raynault MF. Health of migrants with precarious status: results of an exploratory study in Montreal--Part B. Soc Work Public Health. 2012;27(5):469-81.

58. Gordon A, O-Brien C, Balen J, Duncombe SL, Girma A, Mitchell C. A cross-sectional survey of sociodemographic characteristics, primary care health needs and living conditions of asylum-seekers living in a Greek reception centre. Journal of Public Health. 2020:1-11.

59. Hermosa M, Tineo M, Aranda Y, Posada G. Perception of change in living conditions and diet among rural Latino immigrants. Agronomía Colombiana. 2015;33(1):107-12.

60. Jacques-Aviñó C, Peralta A, Carrere J, Marí-Dell'Olmo M, Benach J, López M-J. Qualitative evaluation of an intervention to reduce energy poverty: Effects perceived by participants according to typologies of social vulnerability. Energy Policy. 2022;167:113006.

61. Javanparast S, Naqvi SKA, Mwanri L. Health service access and utilisation amongst culturally and linguistically diverse populations in regional South Australia: a qualitative study. Rural Remote Health. 2020;20(4):5694.

62. Teunissen E, Sherally J, van den Muijsenbergh M, Dowrick C, van Weel-Baumgarten E, van Weel C. Mental health problems of undocumented migrants (UMs) in The Netherlands: a qualitative exploration of help-seeking behaviour and experiences with primary care. BMJ Open. 2014;4(11):e005738.

63. Giansanti E, Lindberg A, Joormann M. The status of homelessness: Access to housing for asylum-seeking migrants as an instrument of migration control in Italy and Sweden. Critical Social Policy. 2022;42(4):586-606.

64. Chiu S, Redelmeier DA, Tolomiczenko G, Kiss A, Hwang SW. The health of homeless immigrants. J Epidemiol Community Health. 2009;63(11):943-8.

65. El-Tawil AM, Nightingale P, Cox MA. Does living in crowded houses offer protection against the development of inflammatory bowel disease? Eur Rev Med Pharmacol Sci. 2013;17(5):632-5.

66. Idemudiai ES. Perceived living conditions and reported feelings of wellbeing among Africans in Germany. Gender and Behaviour. 2009;7(2):2541-56.

67. Mazhak I, Maltseva K, Sudyn D. Assessing psychometric of the perceived stress scale and identifying stress-associated factors in a sample of Ukrainian female refugees in the Czech Republic. J Migr Health. 2024;10:100271.

68. Murphy F, Vieten UM. Asylum seekers and refugees in Northern Ireland: the impact of post-migration stressors on mental health. Ir J Psychol Med. 2022;39(2):163-72.

69. Scales SE, Park JW, Nixon R, Guha-Sapir D, Horney JA. Disease burden among refugees in camps on mainland Greece, 2016-2017: a retrospective cross-sectional study. BMC Public Health. 2023;23(1):1715.

70. Biddle L, Bozorgmehr K. Effect of area-level socioeconomic deprivation on mental and physical health: A longitudinal natural experiment among refugees in Germany. SSM Popul Health. 2024;25:101596.
