# Supplementary material for: Housing inequalities and health outcomes among migrant and refugee populations in high-income countries: a mixed-methods systematic review
Source: BMC Public Health. 2025 Mar 22;25:1098. doi: 10.1186/s12889-025-22186-5 (PMC11929249; doi:10.1186/s12889-025-22186-5)
Supplement: Supplementary file 3 — Supplementary Material 3 [file 12889_2025_22186_MOESM3_ESM.docx]

**Supplementary Table S3.** Characteristics and summary of quantitative studies reviewed

| **Study details** | **Population and setting** | **Study aims** | **Exposure and outcomes/ measures in the study** | **Statistical methods and results/effect estimates** | **Author’s conclusions and**  **reviewer’s comments** |
| --- | --- | --- | --- | --- | --- |
| **Authors:**  Ahmad et al. (1)  **Year of publication:**  2021  **Study design:**  Cohort study  **Country:**  Canada | **Sampling:**  This study included 1924 Syrian refugees in Canada.    **Participants details:**  Participants included Syrian refugees recruited through various community-based strategies in Canada.  **Setting:**  This study was conducted across urban centres in Ontario, Quebec, and British Columbia, Canada. | The primary aim of this study was to evaluate the prevalence of depression-level symptoms at baseline and one-year post-resettlement among Syrian refugees in Canada, and to identify predictors of these symptoms. | **Exposure:**   - Satisfaction with housing condition (Yes/No)   **Outcome:**  Mental health outcomes measured by:   - Prevalence Depression-level symptoms (from PHQ-9 scale) | **Statistical analysis:**  Multinomial logistic regression was used.  **Results/effects estimates:**  **Mental Health:**   - Participants who were not satisfied with housing had higher prevalence of depression at baseline (*p*<0.001) - Participants who were not satisfied with housing had higher prevalence of depression at year 2 follow up (*p*<0.001) - Participants who were satisfied with housing had lower risk of moderate depression at year 2 (RR: 0.48; 95%CIT: 0.31,0.74; *p*<0.001) | **Author’s conclusions:**  *“Increase in depression-level symptoms deserves attention through focusing on identified predictors particularly baseline depression scores, social support, perceived control and language ability.”*  **Reviewer’s comments:**  The findings suggest that Syrian refugees in Canada who were not satisfied with their housing experienced a higher prevalence of depression both at baseline and at the year 2 follow-up, while those satisfied with their housing showed a lower risk of moderate depression in the second year.  **Assessment of methodological quality:**  This study has clearly met 7/11 (64%) criteria in the critical appraisal tool. |
| **Authors:**  Al Masri et al. (2)  **Year of publication:**  2021  **Study design:**  Cross-sectional pilot study  **Country:**  Germany | **Sampling:**  This study included 114 Syrian refugees.    **Participants details:**  This study focused on Syrian refugees residing in and around Hannover, Germany, who lived in Germany for less than four years. Over 85% lived on less than 1000€ income per month.  **Setting:**  This study was conducted at the Institute of Food Science and Human Nutrition, Leibniz University Hannover, Germany, between December 2018 and March 2020. | The primary aim of the study was to assess the Quality of Life (QOL) among Syrian refugees in Germany and to investigate whether general socioeconomic factors (e.g., age, sex, housing, asylum duration) were predictive factors for their QOL. | **Exposure:**   - Type of housing: rented house, rented apartment, and refugee camp.   **Outcome:**   - Health related QoL which included Physical and Psychosocial health score (using the WHOQOL-BREF tool) | **Statistical analysis:**  Spearman Rho correlation coefficient, Kruskal–Wallis, Mann–Whitney U test, and multivariate linear regression were used.  **Results/effects estimates:**  **Psychosocial Health:**   - Housing was one of the key predictors of psychosocial scores (T= -2.40; 95%CI= -2.48, -0.69; *p*=0.018) - Housing was one of the key predictors of overall quality of life (T= -2.75; 95%CI = -2.13, -0.35; *p*=0.008) | **Author’s conclusions:**  *“The Syrian refugees participating in this study showed a low QOL score in the assessment of all domains compared to the normal population, especially regarding social relations and psychological; it was associated with socioeconomic factors, such as housing, asylum duration and marital status. This calls for urgent societal and political efforts to strengthen the social living conditions of Syrian refugees in Germany.”*  **Reviewer’s comments:**  The findings suggest that the type of housing significantly predicted overall quality of life and psychosocial health among Syrian refugees in Germany.  **Assessment of methodological quality:**  This study has clearly met 7/8 (88%) criteria in the critical appraisal tool. |
| **Authors:**  Ambrosetti et al. (3)  **Year of publication:**  2021  **Study design:**  Cohort study  **Country:**  Germany | **Sampling:**  This study included data from the IAB-BAMF-SOEP Survey of Refugees in Germany, including 3,957 individuals with 11,464 observations.    **Participants details:**  This study focused on refugees from Syria, Iraq, Afghanistan, and Eritrea, who arrived in Germany around 2015. It included refugees of various ages and socioeconomic backgrounds.  **Setting:**  The study utilised a longitudinal survey conducted across Germany, focusing on refugees' experiences and outcomes following their arrival in the country. | The primary aim of this study was to examine the impact of pre- and post-arrival mechanisms on refugees' self-rated health and life satisfaction in Germany. | **Exposure:**   - Type of residence (shared/private residence)   **Outcome:**   - Self-rated general health - Overall life satisfaction | **Statistical analysis:**  Linear regression and panel models were used.  **Results/effects estimates:**  **General Health:**   - Living in private flat/house was associated higher self-rated life satisfaction (*p*<0.001) - Living in private flat/house was not associated with self-rated health | **Author’s conclusions:**  *“The time-dynamic analyses reveal substantial improvements in life satisfaction upon the approval of refugee status and the transition from shared housing to private accommodations. However, we find no improvements in self-rated health due to legal status but rather deterioration effects due to long-term residence in shared housing.”*  **Reviewer’s comments:**  The study findings showed that among refugees in Germany, residing in a private flat or house was associated with higher self-rated life satisfaction, but this did not have a significant association with self-rated health.  **Assessment of methodological quality:**  This study has clearly met 8/11 (73%) criteria in the critical appraisal tool. |
| **Authors:**  Bayes-Marin et al. (4)  **Year of publication:**  2022  **Study design:**  Cross- sectional study  **Country:**  Spain | **Sampling:**  This study included 129 migrants residing in Spain.  **Participants details:**  Participants included migrants aged ≥18 years living in Spain during the COVID-19 lockdown period (March–June 2020).  **Setting:**  This study was conducted online using anonymous online survey distributed through the Qualtrics web-based platform and social media using convenience and snowball sampling methods. | The primary aim of this study was to estimate the prevalence of depressive and anxiety symptoms among migrants in Spain and to evaluate the impact of various risk and protective factors on these psychological outcomes. | **Exposure:**   - Type of accommodation (owned/rented/shared)   **Outcome:**   - 8-item Patient Health Questionnaire (PHQ-8) - 7-item General Anxiety Disorder Scale (GAD-7) | **Statistical analysis:**  Multiple Poisson regression models were used to assess the associations between risk and protective factors and depressive and anxiety symptoms, adjusted for age and gender.  **Results/effects estimates:**  **Mental Health:**   - Living in a rented house and other housing (including living in someone’s house, such as family or a friend) were significant risk factors for depressive symptoms (IRR: 1.49, *p*=0.034; IRR: 1.78, *p*=0.006, respectively) compared to living in owned house. | **Author’s conclusions:**  *"These findings addressing risk and protective factors (e.g., social support, self-esteem) help to design culturally effective programs, particularly in migrants with pre-existing mental health conditions, adjusting the organisation of mental healthcare services in difficult times in Spain."*  **Reviewer’s comments:**  The study findings showed that living in rented or shared accommodations were significant risk factors for depressive symptoms compared to owning a house.  **Assessment of methodological quality:**  This study has clearly met 8/8 (100%) criteria in the critical appraisal tool. |
| **Authors:**  Blukacz et al. (5)  **Year of publication:**  2024  **Study design:**  Cross- sectional study  **Country:**  Chile | **Sampling:**  This study included data from 3,555 migrants and 212,346 locals who participated in the nationally representative National Socioeconomic Characterization Survey (CASEN), employing probabilistic, stratified, and multistage sampling methods.  **Participants details:**  Participants included household heads or members aged ≥18 years. The study analysed international migrants, defined as individuals born outside Chile, and compared with local residents, defined as individuals born in Chile. Variables such as health status and housing adequacy were evaluated for both groups.  **Setting:**  The CASEN survey collects socioeconomic and demographic data in Chile through structured interviews. The survey is representative at national, regional, and urban-rural levels and is an evaluation tool for social policies targeting priority groups. Data collection involved face-to-face and telephone-based methods, with the 2020 survey adapting to pandemic conditions using a mixed-mode approach. | The primary aim of this study was to analyse adequate housing as a social determinant of health among international migrants and local populations in Chile. | **Exposure:**  Housing indicators:   - Availability of services, facilities, and infrastructure (sanitation, electricity, water supply) - Habitability (number of people in room) - Location and accessibility (proximity to healthcare facility, transport, education centre) - Affordability   **Outcome:**   - Short-term healthcare needs (illness or accidents in the last 3 months) - Long-term healthcare needs (being under treatment for any pathology in the past year) | **Statistical analysis:**  Logistic regression models were used with short-term health need and long-term health needs as the dependent variable and each of the housing variables (fitted in separate models) as the independent variable. Analyses for 5 cross-sectional surveys (i.e. 2013, 2015, 2017, 2020 and 2022) were reported.  **Results/effects estimates:**  **General Health:**  Results were heterogenous on clustered analysis:   - Living in housing with sanitation facilities were less likely to have long term healthcare needs (2013 OR: 0.23, *p*<0.01) compared to none - Living in housing with public water supply (compared to other supply) were more likely to have short term healthcare needs (2022 OR: 1.22, *p*<0.01) and more likely to have long term healthcare needs (2013 OR: 2.84, *p*<0.01 and 2022 OR: 1.71, *p*<0.01) - Living in housing within 8 blocks or 1 km of public transportation were less likely to have short term healthcare needs (2015 OR: 0.515, *p*<0.05) | **Author’s conclusions:**  *"We found significant inequities in adequate housing between migrant populations and locals in*  *Chile, and some inequities among both populations based on structural socioeconomic deprivation. Experiencing*  *hazardous situations emerged as a social determinant of health among international migrants in 2022, potentially*  *suggesting growing challenges related to social exclusion in urban areas. However, limitations such as exclusion*  *criteria of the survey and sample sizes for data on the migrant population potentially suggest that housing challenges*  *and their impact on health are underestimated."*  **Reviewer’s comments:**  The study findings indicated that living in housing with sanitation facilities was associated with a lower likelihood of long-term healthcare needs, while living in housing with a public water supply and longer distance from public transportation were linked to a higher likelihood of both short-term and long-term healthcare needs.  **Assessment of methodological quality:**  This study has clearly met 8/8 (100%) criteria in the critical appraisal tool. |
| **Authors:**  Cabieses et al. (6)  **Year of publication:**  2012  **Study design:**  Cross-sectional study  **Country:**  Chile | **Sampling:**  This study used data from the CASEN (Caracterización Socio-Economica Nacional) survey conducted in Chile in 2006. The survey used multistage probabilistic sampling with a final sample consisting of 268,873 people from 73,720 households, representing 95.4% of the total Chilean territory.    **Participants details:**  Participants included individuals who self-reported as immigrants (1% of the total sample) and those who preferred not to report their migration status (0.7% of the total sample), across various demographic groups in Chile.  **Setting:**  The study utilised national survey data from Chile, encompassing urban and rural areas across the country. | The primary aim of this study was to explore the living conditions and health of self-reported immigrants and respondents who preferred not to report their migration status in Chile based on the 2006 CASEN survey. | **Exposure:**  Material living standards:   - Housing quality (acceptable/sub-standard/unfit) - Sanitary systems (adequate/deficient) - Overcrowding - Household assets index (HAI)   **Outcome:**  Health outcomes in past year included:   - Any disability - Illness/accident - Hospitalisation/surgery - Cancer/chronic condition - The number of medical/emergency visits | **Statistical analysis:**  Weighted regression models were used.  **Results/effects estimates:**  **General Health:**   - The association of the material living standard with health outcomes in the past year was not maintained in the final model, which was adjusted for age, sex, and urban/rural factors | **Author’s conclusions:**  *“This is the first study to look at the living conditions and health of those that preferred not to respond their migration status in Chile. Respondents that do not report their migration status are vulnerable to poor health and may represent undocumented immigrants. Surveys that fail to identify these people are likely to misrepresent the experiences of immigrants and further quantitative and qualitative research is urgently required.”*  **Reviewer’s comments:**  The study findings, based on the 2006 CASEN survey in Chile, did not establish an association between material living standards and health outcomes in the past year for self-reported immigrants and respondents who preferred not to report their migration status.  **Assessment of methodological quality:**  This study has clearly met 8/8 (100%) criteria in the critical appraisal tool. |
| **Authors:**  Campbell et al. (7)  **Year of publication:**  2018  **Study design:**  Cohort study  **Country:**  UK | **Sampling:**  This study included 5678 participants.    **Participants details:**  Participants were new refugees in the UK, drawn from the Longitudinal Survey of New Refugees, covering new UK refugees between 2005-2007. A postal questionnaire was sent at 4 time points across 2 years.  **Setting:**  This study was set within the UK, monitoring refugee integration and social factors impacting emotional well-being. | The primary aim of this study was to investigate the effect of social determinants such as employment, language ability, and accommodation on the mental health of refugees in the UK. | **Exposure:**   - Satisfaction with current accommodation (Very satisfied/ Fairly satisfied/ Neither satisfied nor dissatisfied/ Slightly dissatisfied/ Very dissatisfied)   **Outcome:**  36-Item Short Form (SF-36) scores:   - Mental health (measured by the emotional wellbeing) | **Statistical analysis:**  Ordered logistic regression models were used.  **Results/effects estimates:**  **Mental Health:**   - Decreased satisfaction with current accommodation was associated with poor emotional wellbeing. Higher odds of poor emotional well-being were seen across Fairly satisfied (OR: 1.68), Neither satisfied and dissatisfied (OR: 2.21), Slightly dissatisfied (OR: 2.54) and Very dissatisfied (OR: 6.15) compared to Very satisfied with accommodation group (*p*<0.0001). | **Author’s conclusions:**  *“Postdisplacement social factors, including language ability, employment status and accommodation satisfaction, were important determinants of refugee emotional well- being. Changes in these social determinants have the potential to improve refugee mental health, making them legitimate, modifiable targets for important public health interventions. Accounting for this, further research into how to improve refugee well-being is crucial given the increase in refugee numbers around the developed world.”*  **Reviewer’s comments:**  The findings from this study indicated that decreased satisfaction with current accommodation was associated with poor emotional well-being among new refugees in the UK.  **Assessment of methodological quality:**  This study has clearly met 8/11 (73%) criteria in the critical appraisal tool. |
| **Authors:**  Cloos et al. (8)  **Year of publication:**  2020  **Study design:**  Cross-sectional study  **Country:**  Canada | **Sampling:**  The study included 806 uninsured migrants in Montreal, Quebec, recruited through venue-based recruitment, snowball strategy, and media announcements.    **Participants details:**  Adult migrants without health insurance, including 53.9% with temporary legal status and 46% without authorised status. Regions of birth included Asia, the Caribbean, Europe, Latin America, the Middle East, Sub-Saharan Africa, and the United States.  **Setting:**  Survey conducted in Montreal, Quebec, targeting uninsured migrants in the city. | The primary aim of this study was to examine the social determinants of self-perceived health among migrants with precarious status in Montreal, who are uninsured. | **Exposure:**   - Insalubrious housing (measured as at least one of the following situation: presence of cockroaches, bedbugs, mice, rats, smell or spots of mould, water infiltration, flooding or water damage, or other situation)   **Outcome:**   - Self-perceived health status | **Statistical analysis:**  Logistic regression was used.  **Results/effects estimates:**  **General Health:**   - Insalubrious housing was not statistically associated with negative self-perceived health status (Yes: OR: 1.16; 95%CI: 0.75, 1.80; *p=*0.504) | **Author’s conclusions:**  *“In our study, almost half of immigrants without health insurance perceived their health as negative, much higher than reports of negative self-perceived health in previous Canadian studies (8.5% among recent immigrants, 19.8% among long-term immigrants, and 10.6% among Canadian-born). Our study also suggests a high rate of unmet health care needs among migrants with precarious status, a situation that is correlated with poor self-perceived health. There is a need to put social policies in place to secure access to resources, health care and social services for all migrants, with or without authorised status.”*  **Reviewer’s comments:**  The study findings indicated that among uninsured migrants in Montreal, Quebec, insalubrious housing was not statistically associated with negative self-perceived health status.  **Assessment of methodological quality:**  This study has clearly met 8/8 (100%) criteria in the critical appraisal tool. |
| **Authors:**  Dudek et al. (9)  **Year of publication:**  2022  **Study design:**  Cross-sectional study  **Country:**  Germany | **Sampling:**  This study included data from 1,535 respondents from 1,159 households.    **Participants details:**  Participants in the study were individuals above 18 years of age with refugee and asylum-seeking backgrounds who arrived in Germany between 2013 and 2016. The majority of participants originated from Syria, Afghanistan, and Iraq.  **Setting:**  This study was based on nation-wide survey of refugees, which is a longitudinal study conducted by Institute for Employment Research, Germany. | The primary aim of this study was to investigate the relationship between different types of refugee accommodations in Germany and their impact on mental and physical health outcomes. | **Exposure:**   - Type of accommodation - Political dimensions (laws and policies in housing context), societal/environmental dimensions (e.g., integration into social and physical environment and the resident living in it) - Institutional dimensions (systems and arrangements within the living space that have the potential to reduce residents’ empowerment and self-determination) - Individual factors (subjective evaluation to housing situation)   **Outcome:**  12-Item Short Form (SF-12) scores**:**   - Mental health (measured by the Mental Health Component Score - MCS) - Physical health (measured by the Physical Health Component Score - PCS) | **Statistical analysis:**  Cluster analysis and linear mixed model regression analysis, to examine the associations between accommodation clusters and mental and physical health outcomes.  **Results/effects estimates:**  **Mental Health:**   - Cluster 2 (with collective accommodation) had the lowest MCS score: 47.1 (SD= 11.9) compared to other cluster (with private accommodation) (t= -2.6; *p*=0.009) - Female gender was significantly associated with poorer mental health (b= -1.98; *p*<0.001) - Originating from Eritrea (b= 3.61; *p*=0.004) and being employed or under training (*p*<0.05 across all cluster) showed positive association with mental health   **Physical Health:**   - Physical health was significantly lower in Cluster 4 (one of the clusters with private accommodation), which was characterised by poor access to public transport and a higher level of restrictions compared to another cluster - There were no significant associations between type of accommodation and physical health | **Author’s conclusions:**  *“We demonstrate that unfavourable conditions cluster in collective accommodation with negative outcomes for mental health but not for physical health. We also found health disparities across types of private accommodation. We conclude that housing plays a role in the production of health inequalities in ASR [Asylum Seekers and Refugees] but needs to be assessed in a differentiated, multidimensional way.”*  **Reviewer’s comments:**  The findings suggest that housing inequalities, as reflected in the different accommodation clusters, are associated with physical and mental health outcomes among migrant and refugee populations. Specifically, living conditions in collective accommodation were associated with poorer mental health, while accommodation with poor access to public transport and a higher level of restrictions was associated with lower physical health.  **Assessment of methodological quality:**  This study has clearly met 8/8 (100%) criteria in the critical appraisal tool. |
| **Authors:**  Eisen et al. (10)  **Year of publication:**  2021  **Study design:**  Cohort study  **Country:**  US | **Sampling:**  This study included 78 torture-surviving asylum seekers.    **Participants details:**  Participants were predominantly from Ethiopia, Eritrea, and Cameroon, and were seeking asylum in the United States and had experienced torture.  **Setting:**  This study was conducted at an agency in the United States providing psychological and case-management services to asylum seekers. | The primary aim of this study was to examine the impact of post-migration factors (housing, employment, asylum status) on the severity of PTSD and depressive symptoms among torture-surviving asylum seekers. | **Exposure:**   - Housing rating scale includes (Homeless and/or resides in a shelter/ Rotates between homes of friends and family/ Has stable housing, but overcrowded apartment/ Stable and appropriate housing (has his/her room/apartment))   **Outcome:**  Mental health outcomes measured by:   - PTSD symptom severity (from HTQ-30 scale) - Depression symptom checklist (from HSCL-25 scale) | **Statistical analysis:**  Multiple regression analysis was used.  **Results/effects estimates:**  **Mental Health:**   - Housing status was not statistically associated with PTSD symptom severity (*b*= -0.104; S.E.= 0.088; *p*=0.236) - Housing status was not statistically associated with Depression symptom severity (*b*= -0.104; S.E.= 0 .105; *p*=0.318) | **Author’s conclusions:**  *“Change in asylum status was found to reduce symptom levels, but other factors were not significantly related to changes in symptom level. These findings highlight the need to better understand the relationship between PM [post-migration] factors and mental health.”*  **Reviewer’s comments:**  The study findings indicate that changes in housing status were not statistically significantly associated with changes in PTSD or depressive symptom levels in asylum seekers in the United States who had experienced torture.  **Assessment of methodological quality:**  This study has clearly met 8/11 (73%) criteria in the critical appraisal tool. |
| **Authors:**  Gillespie et al. (11)  **Year of publication:**  2020  **Study design:**  Cohort study  **Country:**  US and Canada | **Sampling:**  This study included 198 1st and 2nd generation Somali young adults.    **Participants details:**  The sample included 1st and 2nd generation Somali young adults, with an average of 16.35 years of living in North America.  **Setting:**  This study was conducted among Somali refugees and immigrants living in urban areas across North America. | The primary aim of this study was to investigate the effect of past year housing stability on symptoms of PTSD and exposure to neighbourhood violence among these individuals. | **Exposure:**   - Housing stability (if the participants have moved in the past year) - Housing discrimination in the past year   **Outcome:**  Mental health outcomes measured by:   - PTSD symptom severity (from HTQ-30 scale) | **Statistical analysis:**  Analysis of Variance (ANOVA) models and Maximum Likelihood Estimation with Robust standard errors (MLR) were used.  **Results/effects estimates:**  **Mental Health:**   - Forced moves were associated with worsening PTSD symptoms over a year compared to voluntary movers (Wald: 4.389; *p*=0.036). - Voluntary moves were associated with improvements in symptoms in contrast to non-movers (Wald: 4.111; *p*=0.043). | **Author’s conclusions:**  *“Discrimination, neighbourhood violence, economic insecurity, and interpersonal conflict precipitated forced moves. Forced moves were associated with worsening PTSD symptomology over one year, while voluntary moves were associated with improvements in symptoms. The current study provides evidence of the importance of safe, stable housing for the mental health of young adult immigrants.”*  **Reviewer’s comments:**  The study findings indicated that among 1st and 2nd generation Somali young adults in North America, forced moves were associated with worsening PTSD symptoms over a year, while voluntary moves were linked to improvements in symptoms, in contrast to the stable symptoms observed in non-movers.  **Assessment of methodological quality:**  This study has clearly met 7/11 (64%) criteria in the critical appraisal tool. |
| **Authors:**  Handiso et al. (12)  **Year of publication:**  2024  **Study design:**  Cohort study  **Country:**  Australia | **Sampling:**  This study included data from the Building a New Life in Australia (BNLA) project, which includes data on humanitarian migrants resettling in Australia with 2399 participants in wave 1.  **Participants details:**  Participants were humanitarian migrants aged 18 years or older who had recently been granted permanent residency in Australia.  **Setting:**  The study was conducted across 11 sites in Australia, with data collected through computer-assisted self-interviews and telephone interviews. Participants were recruited between October 2013 and March 2014 and followed for 5 years (5 waves). | The primary aim of this study was to identify trends and determinants of mental illness among humanitarian migrants resettled in Australia. | **Exposure:**   - Type of accommodation (temporary/ short-term leases/ long-term leases/ homeownership)   **Outcome:**   - PTSD-8: 8-items Post-Traumatic Stress Disorder questionnaire | **Statistical analysis:**  Generalised linear mixed model was selected using Akaike information criteria and the Log-likelihood ratio test.  **Results/effects estimates:**  **Mental Health:**   - Participants with temporary housing had a higher likelihood of PTSD (OR: 3.7, *p*=0.001), followed by those in short-term leases (OR: 1.3, *p*=0.04) compared with homeownership. - No significant difference in PTSD risk was found for those with long-term leases or homeownership (OR: 0.9, *p*=0.965). | **Author’s conclusions:**  *“The prevalence, persistence and consequential burden of mental illness within this demographic underscore the urgent need for targeted social and healthcare policies. These policies should aim to mitigate modifiable risk factors, thereby alleviating the significant impact of mental health challenges on this population.”*  **Reviewer’s comments:**  The study results indicate that temporary housing and short-term leases are associated with a higher likelihood of PTSD, while no significant difference in PTSD risk was found for long-term leases or homeownership.  **Assessment of methodological quality:**  This study has clearly met 9/11 (82%) criteria in the critical appraisal tool. |
| **Authors:**  Handiso et al. (13)  **Year of publication:**  2024  **Study design:**  Cohort study  **Country:**  Australia | **Sampling:**  This study included data from the Building a New Life in Australia (BNLA) project, which includes data on humanitarian migrants resettling in Australia with 2399 participants in wave 1.  **Participants details:**  Participants were humanitarian migrants aged 18 years or older who had recently been granted permanent residency in Australia.  **Setting:**  The study was conducted across 11 sites in Australia, with data collected through computer-assisted self-interviews and telephone interviews. Participants were recruited between October 2013 and March 2014 and followed for 5 years (5 waves). | The primary aim of this study was to investigate gender disparities in PTSD and psychological distress among humanitarian migrants in Australia. | **Exposure:**   - Type of accommodation (temporary/ short-term leases/ long-term leases/ homeownership)   **Outcome:**   - PTSD-8: 8-items Post-Traumatic Stress Disorder questionnaire - Kessler-6 (K6) scale | **Statistical analysis:**  Generalised linear mixed-effect logistic regression analyses were conducted stratified by gender.  **Results/effects estimates:**  **Mental Health:**   - Men living in long-term leases (OR: 0.5, 95% CI: 0.4–0.7) and those owning homes (OR: 0.2, 95% CI: 0.1–0.3) had lower risk of psychological distress compared to temporary and short-term leases. - Women in short-term lease housing had higher odds of PTSD (OR: 1.6, 95% CI: 1.5–1.7) compared to men in similar housing arrangements (OR: 1.1, 95% CI: 1.05–1.15). | **Author’s conclusions:**  *“Women facing financial hardship, inadequate housing, and unemployment exhibit higher rates of PTSD and psychological distress, underscoring the significant impact of socioeconomic factors. Addressing these challenges at both individual and systemic levels is essential for promoting well-being and managing mental health among female humanitarian migrants.”*  **Reviewer’s comments:**  The study findings suggest that men in long-term leases and homeownership experience lower psychological distress, while women in short-term lease housing have a higher likelihood of PTSD compared to men in similar housing arrangements.  **Assessment of methodological quality:**  This study has clearly met 9/11 (82%) criteria in the critical appraisal tool. |
| **Authors:**  Kang et al. (14)  **Year of publication:**  2022  **Study design:**  Cross-sectional study  **Country:**  South Korea | **Sampling:**  This study included 14,277 participants who were economically active immigrant workers from the 2020 Survey on Immigrants' Living Conditions and Labour Force in Korea.    **Participants details:**  Participants were working-age immigrants (15 years and older) from various nationalities, predominantly Asian. This study included both naturalised citizens and foreigners, including migrant workers and international students.  **Setting:**  The survey was nationwide, including various urban and rural settings across South Korea. | The primary aim of this study was to measure self-rated health among immigrant workers in South Korea and identify factors affecting it. | **Exposure:**   - Residential environment satisfaction (Satisfied/ Mid/ Unsatisfied)   **Outcome:**  **General Health:**   - Self-rated health (Good/ Poor) | **Statistical analysis:**  Multivariate logistic regression with a weighted sampling method was used.  **Results/effects estimates:**  **General Health:**  Residential environment satisfaction was associated with poor self-rated health.   - Mid satisfaction vs. Satisfied (OR: 1.49; 95%CI: 1.47,1.51, *p*<0.001) - Unsatisfied vs. Satisfied (OR: 1.80; 95%CI: 1.74, 1.86, *p*<0.001) | **Author’s conclusions:**  *“To enhance immigrants’ health status, the Korean government must develop strategies to increase their access to healthcare services and minimise unmet healthcare needs. In addition, working conditions must be improved, specifically regarding long working hours and discrimination; furthermore, immigrants’ living environments should be considered.”*  **Reviewer’s comments:**  The study findings indicated that residential environment satisfaction significantly influenced self-rated health among economically active immigrant workers in South Korea, with those expressing mid or unsatisfied levels of residential satisfaction more likely to report poorer health compared to those who were satisfied.  **Assessment of methodological quality:**  This study has clearly met 8/8 (100%) criteria in the critical appraisal tool. |
| **Authors:**  Kearney et al. (15)  **Year of publication:**  2014  **Study design:**  Cross-sectional study  **Country:**  US | **Sampling:**  A total of 352 farmworkers from 183 camps in 16 counties in eastern North Carolina participated.    **Participants details:**  The participants were adult Latino male farm laborers living in employer-provided housing, predominantly from Mexico, and most had H-2A visas.  **Setting:**  The study was conducted among migrant farmworkers living in employer-provided housing in eastern North Carolina. | The primary aim of this study was to evaluate the association between indoor environmental risk factors and respiratory health among migrant farmworker occupants. | **Exposure:**  Poor employer-provided housing condition (Indoor environmental risk factors):   - Presence of cockroaches and rodents - Pesticides - Visible signs of mould - Smoking inside the home   **Outcome:**   - The prevalence of major respiratory symptoms - Lung function measured by spirometry (FEV1, FVC, FEV1/FVC ratio) | **Statistical analysis:**  Bivariate analysis was applied to evaluate associations.  **Results/effects estimates:**  **Physical Health:**  Significant associations were found between:   - Presence of mould and coughing up phlegm (*p*=0.026) - Presence of mould and asthma (*p*=0.008) - Pesticides used in the home and tightness of chest (*p*<0.001) - Use of tobacco and coughing up phlegm (*p*=0.013) | **Author’s conclusions:**  *“Although causal inference can be difficult to establish from a cross-sectional study, findings from this study represents suggestive evidence that indoor environmental risk factors may be contributory factors for respiratory health problems among this vulnerable workgroup population.”*  **Reviewer’s comments:**  This study suggested that indoor environmental risk factors, such as the presence of mould, pests, pesticides, and smoking within the house, might contribute to respiratory health problems among farmworkers in Eastern North Carolina.  **Assessment of methodological quality:**  This study has clearly met 7/8 (88%) criteria in the critical appraisal tool. |
| **Authors:**  Litt et al. (16)  **Year of publication:**  2010  **Study design:**  Population-based descriptive epidemiologic study  **Country:**  US | **Sampling:**  In this study, 250 households participated after canvassing 2,365 addresses. Households were eligible if they had immigrated to the US within the past 10 years, had children aged under 18 years, and primarily spoke Spanish at home.    **Participants details:**  The participants were recent Mexican immigrant families with children and mostly lived in an urban industrial area.  **Setting:**  This study was conducted in Commerce City, Colorado, an urbanised industrial community in Adams County. | The primary aim of the study was to examine home environmental conditions among recently immigrated Mexican families and the health conditions of their children, focusing on asthma and atopic disorders. | **Exposure:**   - Year housing structure built - Basement used as living, play or sleeping space - Housing type - Category of road on which housing structure is located - Overall maintenance - Overall cleanliness - Ventilation potential - Cooling system presence - Observed dampness or mould - Any dampness or mould - Evidence of pests - Any household pets - Any smoking indoors   **Outcome:**   - Asthma and allergy symptoms within the past 12 months - Lifetime diagnoses of asthma, eczema, or allergic rhinitis | **Statistical analysis:**  Taylor expansion technique for calculating design-based estimates of variance was used. Chi-square tests and odds ratios with 95% confidence intervals were used for exploratory analyses.  **Results/effects estimates:**  **Physical Health:**   - Asthma symptoms were reported in 3.9% of children, and atopic symptoms in 9.7% - Homes with minimal or no ventilation potential had higher prevalence of wheezing symptoms (8.5%) - Evidence of pests was associated with increased wheezing (8.1% vs. 2.3%; *p*=0.02) and atopic symptoms (14.9% vs. 7.9%, *p*=0.05) | **Author’s conclusions:**  *“Many of the conditions identified (e.g., pest infestation, mould resulting from plumbing leaks, and lack of exhaust fans) are amenable to low-cost interventions. Solutions to address unhealthy housing conditions among recent immigrants must be multi-faceted and include strategies that target household-level improvements and access to health care.”*  **Reviewer’s comments:**  The study finding indicates that exposure to inadequate housing conditions, such as limited ventilation, dampness, and pests, is significantly associated with increased incidences of respiratory issues in children of Mexican immigrant families in Commerce City, Colorado.  **Assessment of methodological quality:**  This study has clearly met 7/8 (88%) criteria in the critical appraisal tool. |
| **Authors:** Mangrio and Zdravkovic (17)  **Year of publication:** 2018  **Study design:** Cross-sectional study  **Country:** Sweden | **Sampling:**  Approximately 1700 questionnaires were distributed, with 681 returned.  **Participants details:**  This study included recently arrived migrants, mostly consisting of refugees from warzones such as Syria, Iraq, and Afghanistan.  **Setting:**  This study was conducted in Scania region of Sweden, including those in government accommodations and self-arranged apartments and were recruited from the mandatory public integration support programme. | The primary aim of this study was to investigate the association between crowded living conditions among recently arrived migrants in Sweden and mental ill-health. | **Exposure:**   - Crowded living   **Outcome:**  Mental health outcomes measured by:   - Prevalence of poor mental health (from GHQ-12 scale) | **Statistical analysis:**  Multiple logistic regression  **Results/effects estimates:**  **Mental Health**   - Crowded living was associated with poor mental health (OR= 1.46; 95%CI =1.05, 2.03; *p*=0.026) - Crowded living was associated with poor mental health, adjusted for age and educational level (OR= 1.47; 95%CI= 1.05, 2.07; *p*=0.026) | **Author’s conclusions:**  *“The analyses, independent of gender, resulted in a significant unadjusted odds ratio of 1.46 (95% CI 1.05– 2.03); even after adjustments were made, the association remained significant OR 1.47 (1.05–2.07). When adding stability in housing into the adjustment-model, the OR did not remain significant OR 1.40 (0.99–1.99), P-value 0.061.”*  **Reviewer’s comments:**  The study highlights crowded living conditions increase the odds of suffering from mental ill-health among recently arrived migrants in Sweden.  **Assessment of methodological quality:**  This study has clearly met 8/8 (100%) criteria in the critical appraisal tool. |
| **Authors:**  Marchi et al. (18)  **Year of publication:**  2022  **Study design:**  Cross-sectional study  **Country:**  Europe from 46 different countries | **Sampling:**  A total of 3940 participants were included in the analysis.  **Participants details:**  Refugees and other migrants hosted in Europe from 46 different countries.  **Setting:**  The study was conducted using data from the Apart Together online global survey, which was active from April 2020 until November 2020 across various European countries. | The primary aim of this study was to investigate the impact of COVID-19 on mental health and experiences of discrimination among refugees and other migrants in Europe. | **Exposure:**  Socio-demographic hardship (SDH) which included housing situation (house/apartment, asylum centre, refugee camp and on the street)  **Outcome:**   - Self-perceived stigmatisation (SS) (from self-reported experiences) - Psychological distress (PD) (from self-reported experiences) | **Statistical analysis:**  Structural Equation Modelling (SEM) was used for the analysis, with confirmatory factor analysis for latent constructs. Single hold-out sample cross-validation was performed with a train/test split ratio of 0.8/0.2.  **Results/effects estimates:**  **Mental Health:**   - The SDH was strongly associated with psychological distress (B= 0.350; *p*<0.001) and self-perceived stigmatisation (B= 0.274; *p*<0.001) | **Author’s conclusions:**  “*Overall, our findings contribute to understand the condition of refugees and other migrants in the context of COVID-19 pandemic. Those who have more difficulties in accessing health care and preventive measures against the infection experience deterioration of their mental health and increased discrimination by the host population. Refugees and other migrants with a more insecure housing situation and residence status are particularly vulnerable to these negative effects.”*  **Reviewer’s comments:**  This study demonstrates that socio-demographic hardship, specifically insecure housing was significantly associated with worsened mental health and increased experiences of discrimination among refugees and migrants in Europe during the COVID-19 pandemic.  **Assessment of methodological quality:**  This study has clearly met 8/8 (100%) criteria in the critical appraisal tool. |
| **Authors:** Martino et al. (19)  **Year of Publication:** 2022  **Study Design:** Longitudinal study  **Country:** Australia | **Sampling:**  This study used two longitudinal surveys - the Building a New Life in Australia (BNLA) and the Household, Income and Labour Dynamics in Australia (HILDA). There were 21,462 HILDA and 2399 BNLA respondents.  **Participants details:**  Participants were humanitarian migrants in Australia, compared with the general Australian population. BNLA included humanitarian migrants aged 15 and over who settled in Australia between May and October 2013.  **Setting:**   - BNLA focuses on humanitarian migrants who have recently settled in Australia, who have been granted humanitarian visas and are in the process of integrating into Australian society. - HILDA is a nationally representative longitudinal study of Australian households which broadly covers a cross-section of the general population across different regions and communities within Australia. | The primary aim of this study was to examine the effect of precarious housing on the mental health of humanitarian migrants in Australia and compare it with the wider Australian population. | **Exposure:**  Three components of precarious housing   - Affordability - Suitability - Security   **Outcome:**  Mental health outcomes measured by:   - Prevalence of probable serious mental illness (from K6 score in BNLA and K10 in HILDA) | **Statistical Analysis:**  Fixed effects regression analysis was used.  **Results/Effect Estimates:**  **Mental Health:**   - Impact of housing affordability was similar across humanitarian migrants (OR= 1.60; 95%CI= 1.24, 2.06) and wider Australian population (OR= 1.79; 95%CI= 1.38, 2.31) - Unsuitable housing seemed to have a larger adverse effect among humanitarian migrants (OR= 2.38; 95%CI= 1.27, 4.46) compared to wider Australian population (OR= 1.57; 95%CI= 1.22,2.03) | **Author’s conclusions:**  *“Humanitarian migrants were 60% more likely to suffer from worse mental health when they experience unaffordable housing compared to their counterparts in affordable housing, with a 2.4-fold increased risk those in unsuitable housing. Such findings suggest housing precarity can extend the process of liminality for humanitarian migrants, and future policies should facilitate greater access to affordable housing.”*  **Reviewer’s comments:**  This study highlights housing affordability similarly affected the mental health of both humanitarian migrants and the wider Australian population, while unsuitable housing had a more pronounced adverse effect on humanitarian migrants compared to the general population.  **Assessment of methodological quality:**  This study has clearly met 10/11 (91%) criteria in the critical appraisal tool. |
| **Authors:**  Mendola and Busetta (20)  **Year of publication:**  2018  **Study design:**  Cross-sectional study  **Country:**  Italy | **Sampling:**  Survey conducted in 27 informal settlements in Italy, with 565 interviews completed among foreign nationals. Sampling was a stratified two-stage probabilistic sampling.    **Participants details:**  Participants had varying lengths of residence in Italy. Majority had been in Italy for more than three years.  **Setting:**  Informal settlements across Italy, including various types of accommodations, such as buildings, outdoor camps, and tent cities. | The primary aim of this study was to identify informal settlements in Italy hosting foreign nationals living outside the official reception system and assess their living conditions and health status. | **Exposure:**   - Settlement characteristics (poor shelter) - Lack of water/electricity - No. of services in the settlement   **Outcome:**   - Self-reported health status - Occurrence of health problems in the last four weeks | **Statistical analysis:**  Binomial logit models were used to analyse the impact of personal and settlement characteristics on health outcomes.  **Results/effects estimates:**  **General Health:**   - Participants living in a settlement with lack of water/electricity had higher odds of having poor overall health (OR: 4.217, *p*<0.05) - Participants living in a settlement with lack of water/electricity had higher odds of occurrence of health problems (OR: 3.453, *p*<0.05) | **Author’s conclusions:**  *“The poor living conditions of these foreign nationals, their unsatisfied medical needs, and the precariousness of their housing all demonstrate how the Italian reception system is creaking under the growing volume of migrants.”*  **Reviewer’s comments:**  This study demonstrates that poor living conditions in informal settlements in Italy, characterised by a lack of basic amenities, are significantly associated with poor health outcomes.  **Assessment of methodological quality:**  This study has clearly met 8/8 (100%) criteria in the critical appraisal tool. |
| **Authors:**  Mohsenpour et al. (21)  **Year of publication:**  2023  **Study design:**  Cross-sectional study with data linkage  **Country:**  Germany | **Sampling:**  A total of 54 collective refugee accommodation centres were sampled from a total of 1,938 centres, with 70,634 participants. This study employed a complex random sampling design, balancing for the number of asylum seekers and refugees within the centres and their total number in the region.    **Participants details:**  This study included individuals above 18 years of age who were asylum seekers and refugees from various countries.  **Setting:**  This study was conducted across all districts of Baden-Württemberg, which is Germany's 3rd largest federal state. | The primary aim of this study was to assess the impact of housing and environmental factors on the mental health of asylum seekers and refugees. | **Exposure:**   - Tangible factors: Conditions of the housing environment (e.g., state of decay, number of residents, and remoteness of accommodation centres) - Non-tangible factors: Urbanity, district deprivation levels, and housing stability (e.g., number of transfers between accommodation centres)   **Outcome:**  Mental health outcomes measured by:   - Prevalence of symptoms of depression (from PHQ2 questionnaire) - Prevalence of generalised anxiety (from GAD2 questionnaire) | **Statistical analysis:**  Two-level random-intercept logistic regression models were used, including assessments of clustering effects and adjustments for various covariates.  **Results/effects estimates:**  **Mental Health:**   - 45.53% reported symptoms of depression, and 44.83% reported symptoms of anxiety - Highest degree of housing deterioration was associated with higher odds of both generalised anxiety (OR 2.22, CI 0.52-9.59) and depression (OR 1.99, CI 0.55-7.18) - Larger accommodation size had odds of reporting generalised anxiety (OR 1.34, CI 0.59-3.06) and depression (OR 1.12, CI 0.56-2.26) - Remoteness had higher odds for both generalised anxiety (OR 2.16, CI 0.32-14.79) and depression (OR 3.79, CI 0.62-23.18) - District urbanity was associated with higher odds of generalised anxiety (OR 3.05, CI 0.98-9.49) and marginally with depression (OR 1.14, CI 0.46-2.79) - Living in deprived districts showed higher odds for generalised anxiety (OR 1.21, CI 0.51-2.88) but lower odds for depression (OR 0.88, CI 0.41-1.89) | **Author’s conclusions:**  *“We found tendencies for, but no significant, contextual effects of housing environment on ASR [Asylum Seekers and Refugees] mental health in accommodation centres. Confirmatory analyses with prior power calculations are needed to complement these exploratory estimates.”*  **Reviewer’s comments:**  This study highlights a significant link between housing inequalities and mental health issues among migrants and refugees in Germany. The finding indicates that many asylum seekers and refugees experience depression and anxiety, influenced by factors such as poor housing conditions, large and remote accommodation centres, urban settings, and district-level deprivation.  **Assessment of methodological quality:**  This study has clearly met 8/8 (100%) criteria in the critical appraisal tool. |
| **Authors:**  Mohsenpour et al. (22)  **Year of publication:**  2023  **Study design:**  Cross-sectional study  **Country:**  Germany | **Sampling:**  This study analysed data from 412 refugees and asylum seekers residing in up to 58 accommodation facilities in Germany.  **Participants details:**  The participants were refugees and asylum seekers.  **Setting:**  This study was conducted across accommodation facilities in the federal state of Baden-Württemberg, Germany, categorised by urbanity (urban/rural), remoteness (distances to services), and housing conditions assessed by the Small-area Housing Environment Deterioration (SHED) index. | The primary aim of this study was to develop a typology of refugee accommodation based on its physical context and assess its association with health outcomes. | **Exposure:**  Housing and accommodation characteristics:   - Number of inhabitants - Remoteness (walking distance to services) - Urbanity - Housing deterioration (measured using the SHED index: such as broken windows, wall conditions, graffiti, outside space, garbage accumulation, and an overall rating of the physical environment)   **Outcome:**   - General subjective health status - Prevalence of symptoms of depression (from PHQ2 questionnaire) - Prevalence of generalised anxiety (from GAD2 questionnaire) | **Statistical analysis:**  Cluster analysis based on a hierarchal, agglomerative clustering algorithm using Euclidean Distance and Ward’s method were conducted.  **Results/effects estimates:**  **General and Mental Health:**   - Cluster with accommodations with a moderate occupation, lowest levels of deterioration, and a central urban location had higher subjective general health status, depression, and generalised anxiety disorder. | **Author’s conclusions:**  *“Accommodations with a moderate occupation, lowest levels of deterioration, and a central urban location showed the best health outcomes in terms of subjective general health status, depression, and generalized anxiety disorder (GAD). Associations were strongest for GAD and weakest for depression. Our findings inform policymakers on layout and location of refugee collective accommodation centres.”*  **Reviewer’s comments:**  This study highlights accommodations with moderate occupancy, minimal deterioration, and central urban location were linked to better health, lower depression and generalised anxiety disorder.  **Assessment of methodological quality:**  This study has clearly met 8/8 (100%) criteria in the critical appraisal tool. |
| **Authors:**  Montazer (23)  **Year of publication:**  2022  **Study design:**  Cross-sectional study  **Country:**  Canada | **Sampling:**  This study included 1,909 participants, who were residents of the Greater Toronto Area, with a focus on foreign-born individuals, excluding refugees, and using sampling weights to adjust for demographic factors such as nativity, gender and income.  **Participants details:**  The participants were adults aged 25 -64 years, residing in the Greater Toronto Area for at least six months, able to communicate in English, and excluding refugees and adolescents.  **Setting:**  The data were collected from 87 census tracts across the Greater Toronto Area through face-to-face interviews conducted between 2009 and 2011. | The primary aim of this study was to examine if the association between immigration and psychological distress, is altered by homeownership status. | **Exposure:**   - Foreign-born status - Homeownership status   **Outcome:**   - Centre for Epidemiologic Studies Depression Scale - Spielberger Anxiety Scale | **Statistical analysis:**  Hierarchical linear modelling was used to account for clustering of respondents within neighbourhoods.  **Results/effects estimates:**  **Mental Health:**   - Lower psychological distress among foreign-born homeowners compared to renters (*b*=-2.91, *p*<0.05) | **Author’s conclusions:**  *"Adjusted multivariate results indicate lower psychological distress among foreign-born homeowners compared with native-born renters and owners and foreign-born renters. This association is due in part to greater ontological security among immigrant homeowners, compared with renters (native-born and foreign-born) and higher perceived status attainment compared with foreign-born renters. However, contrary to predictions, the lower psychological distress among foreign-born owners, compared with native-born owners, is not due to higher ontological security among this group of the foreign-born compared with their native-born counterparts."*  **Reviewer’s comments:**  The study findings reveal that foreign-born homeowners experience lower psychological distress than renters.  **Assessment of methodological quality:**  This study has clearly met 8/8 (100%) criteria in the critical appraisal tool. |
| **Authors:**  Mora et al. (24)  **Year of publication:**  2016  **Study design:**  Cross-sectional study  **Country:**  US | **Sampling:**  This study involved 371 farmworkers from 186 camps in 16 North Carolina counties. Participants were recruited through community-based participatory research with organisations and clinics serving farmworkers.    **Participants details:**  The participants were male Latino migrant farmworkers, predominantly with H-2A visas, employed in agriculture on a seasonal basis and residing in grower-provided housing or "camps".  **Setting:**  This study was conducted across various agricultural regions in eastern North Carolina. | The primary aim of this study was to examine associations between housing conditions and mental health outcomes (depression, anxiety, alcohol misuse) among Latino migrant farmworkers in North Carolina. | **Exposure:**  Housing conditions:   - Crowding (number of people in same sleeping room) - Perceived security of self and belongings - Possession of a key to dwelling - Bedroom storage - Toilet privacy issues - The number of housing regulation violations   **Outcome:**  Mental health outcomes   - Depression (from CES-D scale) - Anxiety (from PAI scale) - Alcohol misuse (AUDIT-C scale) | **Statistical analysis:**  Rao-Scott chi-square tests and bivariate associations were used.  **Results/effects estimates:**  **Mental Health:**   - Depression was associated with crowded living conditions (*p*=0.01), lack of perceived security (*p*=0.01), and lack of bedroom storage (*p*=0.03) - Anxiety was associated with crowded living conditions (*p*=0.02) and absence of a key to the dwelling (*p*=0.04) - No significant association was found between housing characteristics and alcohol misuse | **Author’s conclusions:**  *“This article suggests links between poor housing and farmworkers’ mental health. These results inform regulations surrounding farmworker housing and inform health care providers on how to prevent and treat poor mental health among migrant farmworkers.”*  **Reviewer’s comments:**  The study findings suggest that depression was linked to crowded living conditions, lack of perceived security, and absence of bedroom storage, while anxiety was associated with crowded living conditions and not having a key to the dwelling, with no significant link between housing conditions and alcohol misuse.  **Assessment of methodological quality:**  This study has clearly met 8/8 (100%) criteria in the critical appraisal tool. |
| **Authors:**  Oudin et al. (25)  **Year of publication:**  2016  **Study design:**  Cross-sectional study  **Country:**  Sweden | **Sampling:**  This study included 359 children, aged 0–13 years.    **Participants details:**  Participants included children from disadvantaged immigrant families residing in two neighbourhoods with differing levels of housing maintenance. The majority of the parents were born outside of Sweden, predominantly in Iraq, Lebanon, and the former Yugoslavia.  **Setting:**  The study recruitment was conducted using local health records and schools in two neighbourhoods of Rosengård, Malmö, Sweden, specifically in the neighbourhoods of Herrgården and Törnrosen. | The primary aim of this study was to describe housing conditions and their association with child health in a disadvantaged immigrant population. | **Exposure:**  Housing conditions:   - Dampness - Mould - Presence of cockroaches - Overcrowding   **Outcome:**  **Child health outcomes:**   - General health - Respiratory conditions (e.g. asthma) - Emergency care visits - Colds - Episode of otitis media - Episode of headaches - Episode of stomach pain - Stress - Concentration difficulties - Difficulty falling asleep - Fatigue | **Statistical analysis:**  Logistic regression analysis was used to estimate odds ratios.  **Results/effects estimates:**  **Physical Health:**   - Significant associations were found between dampness and current asthma (OR= 4.06, 95%CI: 1.67, 9.87) as well as asthma medication (OR= 2.81; 95% CI 1.23, 6.39) - Significant associations were found between mould and headache (OR= 4.2; 95% CI 1.19, 14.81) - Presence of cockroaches was associated with emergency care visits (OR= 4.8; 95% CI 1.63, 14.63), ≥2 colds in the previous 3 months (OR= 2.87; 95% CI 1.01, 8.18), headaches (OR= 5.16; 95% CI 1.93, 13.92), difficulty falling asleep (OR= 8.61; 95% CI 2.52, 29.43) | **Author’s conclusions:**  *“The associations between dampness and asthma, and the association between mould and headache, are in line with current knowledge. The presence of cockroaches seemed to be associated with various outcomes, including those related to mental well-being, which is less described in the literature. The results of the present study are hypothesis generating and provide strong incentives for future studies in this study population.”*  **Reviewer’s comments:**  This study found significant associations between housing dampness and asthma, mould and headaches, and the presence of cockroaches with increased emergency care visits, frequent colds, headaches, and difficulty falling asleep in children.  **Assessment of methodological quality:**  This study has clearly met 8/8 (100%) criteria in the critical appraisal tool. |
| **Authors:**  Raphael et al. (26)  **Year of publication:**  2020  **Study design:**  Cohort study  **Country:**  Sweden | **Sampling:**  Adult immigrants who arrived in Sweden during 1987–1991. This study included 48,056 refugees and 97,254 nonrefugee immigrants.    **Participants details:**  Adult immigrants, both refugees and nonrefugee immigrants, with a larger proportion of refugees being male, having lower educational attainment, and larger families. Most refugees were from Iran, the Middle East, and North Africa.  **Setting:**  Sweden, with immigrants dispersed across various neighbourhoods as part of a national policy. | The primary aim of this study was to estimate the effect of neighbourhood deprivation on mental health among refugees and compare these estimates to nonrefugee immigrants. | **Exposure:**  Neighbourhood deprivation, assessed upon arrival in Sweden and 10 years after arrival, categorised into tertiles.  **Outcome:**  Diagnosis of depression and/or anxiety. | **Statistical analysis:**  Cox models were used to estimate the association and adjusted for various covariates.  **Results/effects estimates:**  **Mental Health:**   - For nonrefugee immigrants, moderate-deprivation neighbourhoods on arrival were associated with greater depression/anxiety compared with low-deprivation neighbourhoods (HR= 1.04; 95% CI= 1.00, 1.09). - For nonrefugee immigrants, ten years later, high (HR= 1.12; 95% CI= 1.07, 1.17) and moderate neighbourhood deprivation (HR= 1.10; 95% CI= 1.06, 1.15) had associations with depression/anxiety compared to low deprivation group. | **Author’s conclusions:**  *“By demonstrating differences using two analytic approaches, we provide in- sight into self-selection bias in the prior neighbourhood-health literature, and we shed light on the possible differential effect of neighbourhood deprivation on de- pression/anxiety by immigration status.”*  **Reviewer’s comments:**  This study suggests nonrefugee immigrants in Sweden, living in deprivation neighbourhoods upon arrival and ten years later were associated with increased rates of depression and anxiety compared to those in low-deprivation areas.  **Assessment of methodological quality:**  This study has clearly met 8/11 (73%) criteria in the critical appraisal tool. |
| **Authors:**  Richter et al. (27)  **Year of publication:**  2018  **Study design:**  Cross-sectional study  **Country:**  Sweden | **Sampling:**  This study included 650 participants from 130 families.    **Participants details:**  Immigrants, and all children were under 13 years of age.  **Setting:**  This study was conducted in the immigrant neighbourhood of Rosengård, particularly focusing on the Herrgården area known for substandard housing, where participants were recruited via health care records for children with respiratory diseases and school lists for asymptomatic children. | The primary aim of this study was to investigate atopic disorders and sensitisations in a population living in substandard housing and their relation to exposures from the built environment. | **Exposure:**  Physical apartment characteristics   - Number of rooms - Number of regular occupants - Presence of pests - Dampness - Mould   **Outcome:**   - Prevalence of atopic disorders - Atopic sensitisation | **Statistical analysis:**  Chi-squared tests for categorical data, and independent t-tests/one-way ANOVA for numerical data were conducted.  **Results/effects estimates:**  **Physical Health:**   - Children living in apartments with moisture (*p*=0.017) and mould (*p*=0.022) exposures were more likely to be sensitised compared to those living in apartments without these exposures. | **Author’s conclusions:**  *“The atopic burden in this selected immigrant population was high, and results point to unmet medical needs. Health care systems caring for such populations need to be aware of their specific health needs; comprehensive asthma and allergy care should include consideration of harmful environmental exposures, adhering to the precautionary principle.”*  **Reviewer’s comments:**  This study concluded that in Sweden, children in immigrant families residing in apartments exposed to moisture and mould demonstrated a higher likelihood of atopic sensitisation compared to those living in environments free of such exposures.  **Assessment of methodological quality:**  This study has clearly met 8/8 (100%) criteria in the critical appraisal tool. |
| **Authors:**  Sandberg et al. (28)  **Year of publication:**  2014  **Study design:**  Cross-sectional study  **Country:**  US | **Sampling:**  This study included 371 male Latino farmworkers in North Carolina during the 2010 agricultural season.    **Participants details:**  Participants were Spanish-speaking, male, Latino, migrant farmworkers, predominantly engaged in agricultural labour.  **Setting:**  This study was conducted in 16 counties across the North Carolina Piedmont and Coastal Plain regions. | The primary aim of this study was to investigate the relationships between sleep quality and variables such as housing quality, health conditions, and individual characteristics. | **Exposure:**  Housing quality:   - Air conditioning availability and usage - Number of people per housing unit - Sleeping room conditions   **Outcome:**  Good sleep quality   - Sleep Timing - Sleep Quality Screening Questionnaire (STSQS) - Sleep quality and association with elevated depressive symptoms, musculoskeletal pain, and greater anxiety. | **Statistical analysis:**  Bivariate and multivariate analyses using Generalised Estimating Equations (GEE) were used.  **Results/effects estimates:**  **Mental Health:**   - Elevated depressive symptoms and anxiety (*p*<0.0001) were associated with poor sleep quality   **Physical Health:**   - Air conditioning availability and usage was associated with good quality sleep (OR: 2.43, 95%CI: 1.33, 4.43). - Musculoskeletal pain (*p*<0.0001) was associated with poor sleep quality | **Author’s conclusions:**  *“Poor sleep quality among Latino farmworkers was associated with poorer indicators of health. One important indicator of housing quality, air conditioning, was associated with better sleep quality. Further research is required to delineate how to improve the adequacy of farmworker housing to improve sleep quality and other health indicators.”*  **Reviewer’s comments:**  This study concluded that poor sleep quality among Latino farmworkers in North Carolina was linked to poorer physical and mental health outcomes. Availability of air conditioning in housing was associated with better sleep quality.  **Assessment of methodological quality:**  This study has clearly met 8/8 (100%) criteria in the critical appraisal tool. |
| **Authors:**  Song et al. (29)  **Year of publication:**  2015  **Study design:**  Cross-sectional study  **Country:**  US | **Sampling:**  This study included 278 participants referred from resettlement agencies to a community clinic in the US.    **Participants details:**  The sample was heterogeneous, including individuals from various countries such as Iraq, Iran, Eritrea, and 35 other countries. These participants were torture survivors who had been displaced and resettled.  **Setting:**  This study was conducted at a community-based non-profit clinic in California, which is the largest provider of services (social, legal, medical, psychological) to refugees and asylum seekers in Northern California. | The primary aim of this study was to investigate the relationships between sociodemographic factors, pre- and post-migration variables, and the prevalence of psychological distress and global functioning in a diverse sample of torture survivors. | **Exposure:**  Post migration housing status (stable/unstable)  **Outcome:**  Mental health outcomes measured by:   - Prevalence of PTSD (from PTSD scale) - Prevalence of symptoms of depression (from HSCL-25 questionnaire) - Prevalence of symptoms of anxiety (from HSCL-25 questionnaire) - General functioning (from GAF questionnaire) | **Statistical analysis:**  Univariate and multivariate logistic regression analyses were used.  **Results/effects estimates:**  **Mental Health:**   - Participants with unstable housing were over twice likely to experience severely impaired global functioning compared to those with stable housing (OR= 2.21; 95%CI= 1.08, 4.53; *p*<0.05) | **Author’s conclusions:**  *“Findings highlight the importance of examining post-migration variables such as length of time in country prior to receiving services in addition to pre- migration torture history upon relocated torture survivors. Clinicians and policymakers should be aware of the importance of early mental health screening and intervention on reducing the psychiatric burden associated with torture and forced relocation.”*  **Reviewer’s comments:**  The study findings demonstrate that among refugees and asylum seekers who have survived torture, unstable housing is significantly associated with impaired global mental health functioning.  **Assessment of methodological quality:**  This study has clearly met 8/8 (100%) criteria in the critical appraisal tool. |
| **Authors:**  Song et al. (30)  **Year of publication:**  2018  **Study design:**  Cross-sectional study  **Country:**  US | **Sampling:**  This study included 278 participants referred from resettlement agencies to a community clinic in the US.    **Participants details:**  The sample was heterogeneous, including individuals from various countries such as Iraq, Iran, Eritrea, and 35 other countries. These participants were torture survivors who had been displaced and resettled.  **Setting:**  The study was conducted at a community-based non-profit clinic in California, which is the largest provider of services (social, legal, medical, psychological) to refugees and asylum seekers in Northern California. | The primary aim of this study was to investigate the impact of sociodemographic, premigration, postmigration, and psychosocial factors on the mental health (anxiety, PTSD, depression) of refugees and asylum seekers who had been exposed to torture. | **Exposure:**  Post migration housing status (stable/unstable)  **Outcome:**  Mental health outcomes measured by:   - Prevalence of PTSD (from PTSD scale) - Prevalence of symptoms of depression (from HSCL-25 questionnaire) - Prevalence of symptoms of anxiety (from HSCL-25 questionnaire) | **Statistical analysis:**  Hierarchical linear regressions were conducted.  **Results/effects estimates:**  **Mental Health:**   - Unstable housing was associated with greater severity of anxiety (B= -0.13; SE= 0.11; *p*<0.05) | **Author’s conclusions:**  *“Cumulative exposure to multiple torture types predicted anxiety and PTSD, while mental health, basic resources (access to food, shelter, medical care), and external risks (risk of being victimized at home, community, work, school) were the strongest psychosocial predictors of anxiety, PTSD, and depression. Also, time spent in the United States before presenting for services significantly predicted anxiety, PTSD, and depression. Consequently, public-sector services should seek to engage this high-risk population immediately upon resettlement into the host country using a mental health stepped care approach.”*  **Reviewer’s comments:**  The study findings demonstrate that among refugees and asylum seekers who have survived torture, unstable housing is significantly associated with anxiety.  **Assessment of methodological quality:**  This study has clearly met 8/8 (100%) criteria in the critical appraisal tool. |
| **Authors:**  Srirangson et al. (31)  **Year of publication:**  2013  **Study design:**  Cross-sectional study  **Country:**  Canada | **Sampling:**  This study involved a non-probability sample of 145 adult Thai immigrants living in the Greater Toronto Area.    **Participants details:**  This study included adult Thai immigrants aged between 20 and 65 years, who had been living in Ontario for at least six months.  **Setting:**  Participants were identified through a Thai community directory and personal contacts during social and religious events in the Greater Toronto Area. | The primary aim of this study was to examine the level of mental health problems among Thai immigrants in Toronto and explore the socio-economic, demographic, immigration, acculturation, and coping resource factors associated with these mental health issues. | **Exposure:**   - Homeownership (own, rental and shared rental) - Quality of neighbourhood (safety, amenities, parks, and public transport)   **Outcome:**   - Mental health (from GHQ-28) | **Statistical analysis:**  Multiple logistic regression analyses were used.  **Results/effects estimates:**  **Mental Health:**   - People with rental housing were associated with increased risk of mental health issues compared to people who owned the house (B= 1.32; SE= 0.77; *p*<0.05) | **Author’s conclusions:**  *“Participants working multiple jobs and renting an accommodation also had an increased risk of psychiatric disorder. Community-based intervention programmes that focus on developing social support and personal mastery among Thai immigrants are recommended.”*  **Reviewer’s comments:**  The study findings demonstrate people with rental housing were more likely to be associated with mental health issues compared to those who owned their homes among Thai immigrants living in the Greater Toronto Area.  **Assessment of methodological quality:**  This study has clearly met 8/8 (100%) criteria in the critical appraisal tool. |
| **Authors:**  Sundquist et al. (32)  **Year of publication:**  1995  **Study design:**  Cross-sectional study  **Country:**  Sweden, Chile and Uruguay | **Sampling:**  This study included 338 Latin American refugees, 60 repatriated refugees, 161 South European labour migrants, 396 Finnish labour migrants, and 1159 age-, sex-, and education-matched Swedes.    **Participants details:**  The participants were diverse in terms of ethnicity and were aged between 16 and 74 years.  **Setting:**  This study was conducted in Lund, a medium-sized town in southern Sweden, Santiago (capital of Chile) and Montevideo (capital of Uruguay), and across Sweden. | The primary aim of this study was to examine differences in living conditions and self-rated health among South European labour migrants, Latin American refugees, repatriated Latin Americans, and Swedes. | **Exposure:**  Living conditions   - Crowding (number of residents) - Home ownership (privately owned, cooperative housing, renting)   **Outcome:**   - Self-rated health status | **Statistical analysis:**  Logistic regression and Mantel-Haenzel chi-square test were used.  **Results/effects estimates:**  **General Health:**   - There was statistically different proportion of Latin American refugees, South European labour migrants, Finnish labour migrants in terms of private home ownership and number of people in residence compared to that of matched Swedes. - Latin American refugees (OR= 2.65; 95%CI= 1.89, 3.56), South European (OR= 2.38; 95%CI= 1.71, 3.21) and Finnish labour migrants (OR= 2.70; 95%CI= 1.75, 4.00) had increased odds of self-rated poor health compared to matched Swedes, however, statistically significant difference was not seen in repatriated Latin American refugees. | **Author’s conclusions:**  *“This study shows a clear ethnic segregation in housing and other living conditions between Swedes and immigrants, where Latin American refugees and repatriated Latin Americans were most vulnerable. All immigrants had increased self-rated poor health compared with Swedes. Being an immigrant was a risk factor for poor health of equal importance to more traditional risk factors such as lifestyle factors.”*  **Reviewer’s comments:**  This study showed that Latin American refugees, South European, and Finnish labour migrants in Sweden had significantly different living conditions and higher odds of self-rated poor health compared to matched native Swedes, with no significant difference noted in repatriated individuals.  **Assessment of methodological quality:**  This study has clearly met 8/8 (100%) criteria in the critical appraisal tool. |
| **Authors:**  Tortelli et al. (33)  **Year of publication:**  2021  **Study design:**  Cross-sectional study  **Country:**  France | **Sampling:**  Including 321 cases of first-episode psychosis, this study analysed data collected over a period with 805,396 person-years at risk.    **Participants details:**  The study participants were immigrants, descendants of immigrants, and the majority population in France.  **Setting:**  This study was conducted in the 20th district of Paris and in the Val-de-Marne area, a metropolitan Paris suburb. | The primary aim of this study was to explore the impact of socioeconomic environment and living conditions on the risk of psychosis. | **Exposure:**  Housing factor:   - Unstable housing - Residing in deprived area   **Outcome:**  Incidence of first-episode psychosis | **Statistical analysis:**  Chi-square tests for sociodemographic characteristics, and Poisson regression for calculating incidence rates (IR) and incidence rate ratios (IRR) were used.  **Results/effects estimates:**  **Mental Health:**   - People with unstable housing status were mostly immigrants (82%) from sub-Saharan Africa (54%). The incidence of psychosis was higher among this group. - The incidence of first episode psychosis was higher in people residing in deprived areas than in nondeprived areas (IRR = 1.3, 95% CI: 1.0–1.6). | **Author’s conclusions:**  *“The current study shows that the increased risk of psychosis in groups with an immigration background in France is associated with their origin and highlights the importance of socioeconomic factors in modulating this risk.”*  **Reviewer’s comments:**  This study indicates that people with unstable housing status, predominantly immigrants from sub-Saharan Africa in France, had a higher incidence of first episode psychosis, as well as among people residing in deprived areas compared to non-deprived areas.  **Assessment of methodological quality:**  This study has clearly met 8/8 (100%) criteria in the critical appraisal tool. |
| **Authors:**  Vignier et al. (34)  **Year of publication:**  2022  **Study design:**  Cross-sectional survey  **Country:**  France | **Sampling:**  This study included 1,223 undocumented migrants recruited in Paris and the Bordeaux region.    **Participants details:**  Participants were predominantly from sub-Saharan Africa and North Africa. Most had arrived in France within the past 3 years.  **Setting:**  The survey was conducted among undocumented migrants in 63 facilities likely frequented by them, including healthcare centres, social service centres, and community organisations in Paris and Bordeaux. | The primary aim of this study was to report frequency of infectious diseases and their associated factors among undocumented migrants in France. | **Exposure:**   - Type of housing (Ordinary, Collective and Makeshift)   **Outcome:**   - Prevalence of infectious diseases (HIV, Chronic HBV and Chronic HCV) | **Statistical analysis:**  Weighted percentages and logistic regression analyses were used.  **Results/effects estimates:**  **Physical Health:**   - Type of housing was not associated with the prevalence of chronic viral infections (HIV, Chronic HBV and Chronic HCV) in this population group (*p*=419) | **Author’s conclusions:**  *“This original study on a large random sample confirms the frequency of infectious diseases among undocumented migrants in France and the importance of integrating their screening during a health Rendezvous and their management into early access to care and inclusive medico-psycho- social management.”*  **Reviewer’s comments:**  This study does not establish an association of housing factors with the prevalence of viral infection.  **Assessment of methodological quality:**  This study has clearly met 8/8 (100%) criteria in the critical appraisal tool. |
| **Authors:**  Walther et al. (35)  **Year of publication:**  2020  **Study design:**  Cross-sectional study  **Country:**  Germany | **Sampling:**  This study surveyed 4,325 adult refugees who arrived in Germany between 2013 and 2016.  **Participants details:**  Participants were adult refugees, predominantly asylum seekers, who arrived in Germany from 2013 to 2016.  **Setting:**  This study was conducted using computer-assisted face-to-face interview format in five different languages across various locations in Germany where the refugees were residing. | The primary aim of this study was to investigate how different living conditions, especially those subject to integration policies, are associated with psychological distress and life satisfaction among refugees in Germany. | **Exposure:**   - Type of accommodation (refugee housing facility or private accommodation)   **Outcome:**  Psychological distress   - Prevalence of depression and anxiety (PHQ-4) | **Statistical analysis:**  Linear regression models were used.  **Results/effects estimates:**  **Mental Health:**  Type of accommodation was associated with higher levels of psychological distress and interacted with number of flight reasons (various factors or circumstances that prompted the refugees to flee their home countries)   - Stratified one or none (B = -0.299, CI= -0.577, - 0.02) - Stratified two or three (B = -0.586, CI= -1.019, -0.152) - Stratified four or five (B =-0.660, CI= -1.010, -0.310) | **Author’s conclusions:**  *“Our findings show that an uncertain legal status, separation from family, and living in refugee housing facilities are related to higher levels of distress and decreased life satisfaction. Being employed, contact to members of the host society, and better host country language skills, by contrast, are related to reduced distress and higher levels of life satisfaction. These associations should inform decision making in a highly contested policy area.”*  **Reviewer’s comments:**  This study showed that living in refugee housing facilities as opposed to private housing, are associated with higher levels of psychological distress and interacted with number of factors leading them to flee their country among refugees in Germany.  **Assessment of methodological quality:**  This study has clearly met 8/8 (100%) criteria in the critical appraisal tool. |
| **Authors:**  Walther et al. (36)  **Year of publication:**  2020  **Study design:**  Cross-sectional study  **Country:**  Germany | **Sampling:**  This study analysed data from 2569 adult refugees who arrived in Germany between 2013 and 2016. Participants were selected from the second wave of the IAB-BAMF-SOEP refugee survey.    **Participants details:**  The participants included adult refugees primarily from Syria, Afghanistan, and Iraq.  **Setting:**  The survey was conducted across Germany, including individuals in various living situations such as private housing and refugee housing facilities. | The primary aim of this study was to estimate the prevalence of psychological distress among a large refugee population in Germany and to assess its association with factors amenable to policy intervention and integration indicators. | **Exposure:**   - Type of accommodation (refugee housing facility or private accommodation)   **Outcome:**  Psychological distress   - Psychological distress (PHQ-4) | **Statistical analysis:**  Multivariable robust Poisson regression models was used.  **Results/effects estimates:**  **Mental Health:**   - Living in a refugee housing facility as compared to private accommodation was associated with increased psychological distress (RR= 1.21; 95%CI= 1.02 to 1.43) | **Author’s conclusions:**  *“The finding that a substantial minority of refugees in Germany exhibits symptoms of distress calls for an expansion of mental health services for this population. Service providers and policymakers should consider the increased prevalence among female, older and Afghan refugees, as well as among single males, residents in housing facilities and those under threat of deportation. The associations between mental health and integration processes such as labour market, educational programme and integration course participation also warrant consideration.”*  **Reviewer’s comments:**  This study suggests that living in a refugee housing facility, as compared to private accommodation, is associated with increased psychological distress in adult refugees who arrived in Germany between 2013 and 2016.  **Assessment of methodological quality:**  This study has clearly met 8/8 (100%) criteria in the critical appraisal tool. |
| **Authors:**  Whitsett and Sherman (37)  **Year of publication:**  2017  **Study design:**  Cohort study  **Country:** US | **Sampling:**  This study included 105 patients referred to specialised outpatient clinic.    **Participants details:**  Asylum seekers from various countries, primarily survivors of government-sponsored torture, seeking legal asylum in the US.  **Setting:**  This study was conducted at a grant-funded outpatient clinic in the US, specialising in treating survivors of torture, referred by immigration lawyers or family members. | The primary aim of this study was to examine if resettlement factors such as housing conditions, language fluency, and legal authorisation to work predict mental health treatment outcomes in asylum-seeking torture survivors. | **Exposure:**   - Housing stability (stable, uncrowded housing conditions)   **Outcome:**  Mental health outcomes measured by:   - Changes in depression symptoms (from HSCL-25 questionnaire) - Changes in anxiety symptoms (from HSCL-25 questionnaire) - Changes in PTSD symptoms (from HTQ questionnaire) | **Statistical analysis:**  Paired t-tests and multiple linear regression analyses were used.  **Results/effects estimates:**  **Mental Health:**   - Depression: For individuals with stable, uncrowded housing at intake, there was negative prediction for final depression scores (B = −0.360, 95% CI= −0.660, −0.061, *p*<0.05) - Anxiety: Individuals with stable, uncrowded housing was the only other significant predictor of final anxiety scores, with a moderate effect (B = −0.338, 95% CI = −0.649, −0.028, *p*<0.05) - PTSD: Individuals with stable, uncrowded housing was found to significantly predict lower levels of PTSD symptoms at reassessment. The effect was moderate and negative (B = −0.388, 95% CI = −0.679, −0.097, *p*<0.01) | **Author’s conclusions:**  *“These findings support the hypotheses that individuals seeking asylum within the United States who have survived torture can benefit from psychiatric treatment and emphasize the importance of stable living conditions in improving treatment effectiveness. This suggests the need for further research on social predictors of treatment outcomes, as well as the need for clinicians and policymakers to target improved housing as a potentially important tool to reduce psychiatric problems related to torture and forced migration.”*  **Reviewer’s comments:**  The study findings suggest that stable and uncrowded housing conditions significantly predict better mental health outcomes, with individuals in stable housing showing greater improvements in depression, anxiety, and PTSD symptoms.  **Assessment of methodological quality:**  This study has clearly met 8/11 (73%) criteria in the critical appraisal tool. |
| **Authors:**  Wirehag et al. (38)  **Year of publication:**  2021  **Study design:**  Cross-sectional study  **Country:**  Sweden | **Sampling:**  This study included 104 undocumented migrants (UMs) using convenience sampling.    **Participants details:**  The participants were adult UMs in Sweden, comprising both men and women. They were undocumented due to various reasons such as rejected asylum applications or overstayed visas.  **Setting:**  This study was conducted in the three largest cities in Sweden - Gothenburg, Stockholm, and Malmö. | The primary aim of this study was to explore the association between the housing situation and mental health of undocumented migrants. | **Exposure:**  Housing situations of UMs:   - Temporary accommodation - Living with friends - Shelters   **Outcome:**  Mental health outcomes measured by:   - Changes in depression symptoms (from Beck’s Depression Inventory) - Changes in anxiety symptoms (from Beck’s Anxiety Inventory) - Changes in PTSD symptoms (from PTSD Checklist) | **Statistical analysis:**  Multivariate linear regression was used.  **Results/effects estimates:**  **Mental Health:**   - Living in temporary accommodation was linked to higher scores on the scales for depression (*p*=0.011), anxiety (*p*=0.031) and PTSD (*p*=0.050) | **Author’s conclusions:**  *“Depression, anxiety, and post-traumatic stress disorder are more common among UMs [Undocumented Migrants] who are living in temporary accommodation or sharing a flat with others than among those who do not. UMs are living in housing situations that would force local social service to act were it not that they have UM status.”*  **Reviewer’s comments:**  This study suggests that living in temporary accommodation was associated with higher scores on the scales for depression among adult undocumented migrants in Sweden.  **Assessment of methodological quality:**  This study has clearly met 6/8 (75%) criteria in the critical appraisal tool. |
| **Abbreviations:**  ANOVA: Analysis of Variance; AUDIT-C: Alcohol Use Disorders Identification Test Consumption scale; B: Beta (Coefficient in regression analysis); BNLA: Building a New Life in Australia; CASEN: Caracterización Socio-Economica Nacional; CI: Confidence Interval; CES-D: Centre for Epidemiologic Studies Depression Scale; FEV1: Forced Expiratory Volume in one second; FVC: Forced Vital Capacity; GAD2; Generalised Anxiety Disorder-2; GAD-7: General Anxiety Disorder Scale 7-item; GAF: Global Assessment of Functioning; GEE: Generalised Estimating Equations; GHQ-12: General Health Questionnaire 12 items; GHQ-28: General Health Questionnaire 28 items; HAI: Household Assets Index; HBV: Hepatitis B Virus; HCV: Hepatitis C Virus; HILDA: Household, Income and Labour Dynamics in Australia; HIV: Human Immunodeficiency Virus; HSCL-25: Hopkins Symptom Checklist- 25 item; HTQ-30: Harvard Trauma Questionnaire – 30 items; IR: Incidence Rate; IRR: Incidence Rate Ratios; K6: Kessler Psychological Distress Scale-6 items; K10: Kessler Psychological Distress Scale-10 items; MCS: Mental Health Component Score; MLR: Maximum Likelihood Estimation with Robust standard errors; MSPA: Migrant and Seasonal Agricultural Worker Protection Act; OR: Odd’s Ratio; PAI: Personality Assessment Inventory; PCS: Physical Health Component Score; PHQ2: Public Health Questionnaire-2; PHQ-4: Patient Health Questionnaire for Depression and Anxiety; PHQ-8: Patient Health Questionnaire-8; PHQ-9: Patient Health Questionnaire 9; PTSD: Post-Traumatic Stress Disorder; PTSD-8: Post-Traumatic Stress Disorder-8; QoL: Quality of life; RHS-13: Refugee Health Screener 13 items; RR: Risk Ratio; SEM: Structural Equation Modelling; SF-12: 12-Item Short Form Health Survey; SF-36: 36-Item Short Form Health Survey; SHED: Small-area Housing Environment Deterioration; SS: Self-perceived Stigmatisation; STSQS: Sleep Quality Screening Questionnaire; UM: Undocumented Migrants; US: United States of America; WHO: World Health Organisation; y: years. | | | | | |

**References:**

1. Ahmad F, Othman N, Hynie M, Bayoumi AM, Oda A, McKenzie K. Depression-level symptoms among Syrian refugees: findings from a Canadian longitudinal study. Journal of Mental Health. 2021;30(2):246-54.

2. Al Masri F, Müller M, Nebl J, Greupner T, Hahn A, Straka D. Quality of life among Syrian refugees in Germany: a cross-sectional pilot study. Archives of Public Health. 2021;79(1):213.

3. Ambrosetti E, Dietrich H, Kosyakova Y, Patzina A. The Impact of Pre- and Postarrival Mechanisms on Self-rated Health and Life Satisfaction Among Refugees in Germany. Front Sociol. 2021;6:693518.

4. Bayes-Marin I, Roura-Adserias M, Giné-Vázquez I, Villalobos F, Franch-Roca M, Lloret-Pineda A, et al. Factors Associated with Depression and Anxiety Symptoms among Migrant Population in Spain during the COVID-19 Pandemic. Int J Environ Res Public Health. 2022;19(23).

5. Blukacz A, Oyarte M, Cabieses B. Adequate housing as a social determinant of the health of international migrants and locals in Chile between 2013 and 2022. BMC Public Health. 2024;24(1):2021.

6. Cabieses B, Pickett KE, Tunstall H. What are the living conditions and health status of those who don't report their migration status? A population-based study in Chile. BMC Public Health. 2012;12:1013.

7. Campbell MR, Mann KD, Moffatt S, Dave M, Pearce MS. Social determinants of emotional well-being in new refugees in the UK. Public Health. 2018;164:72-81.

8. Cloos P, Ndao EM, Aho J, Benoît M, Fillol A, Munoz-Bertrand M, et al. The negative self-perceived health of migrants with precarious status in Montreal, Canada: A cross-sectional study. PLoS One. 2020;15(4):e0231327.

9. Dudek V, Razum O, Sauzet O. Association between housing and health of refugees and asylum seekers in Germany: explorative cluster and mixed model analysis. BMC Public Health. 2022;22(1):48.

10. Eisen E, Howe G, Cogar M. The Impact of Post-Migration Factors on Posttraumatic Stress and Depressive Symptoms among Asylum Seekers in the United States. Journal of Immigrant & Refugee Studies. 2021;19(4):573-86.

11. Gillespie S, Cardeli E, Sideridis G, Issa O, Ellis BH. Residential mobility, mental health, and community violence exposure among Somali refugees and immigrants in North America. Health Place. 2020;65:102419.

12. Handiso DW, Paul E, Boyle JA, Shawyer F, Meadows G, Enticott JC. Trends and determinants of mental illness in humanitarian migrants resettled in Australia: Analysis of longitudinal data. Int J Ment Health Nurs. 2024;33(5):1418-34.

13. Handiso DW, Boyle JA, Paul E, Shawyer F, Enticott JC. Gender disparity and post-traumatic stress disorder and elevated psychological distress in humanitarian migrants resettled in Australia: the moderating role of socioeconomic factors. Epidemiol Psychiatr Sci. 2024;33:e60.

14. Kang SJ, Hwang J, Kim D, Kim B. Factors associated with self-rated health among immigrant workers in South Korea: Analyzing the results of the 2020 survey on immigrants' living conditions and labor force. Frontiers in Public Health. 2022;10.

15. Kearney GD, Chatterjee AB, Talton J, Chen H, Quandt SA, Summers P, et al. The association of respiratory symptoms and indoor housing conditions among migrant farmworkers in eastern North Carolina. J Agromedicine. 2014;19(4):395-405.

16. Litt JS, Goss C, Diao L, Allshouse A, Diaz-Castillo S, Bardwell RA, et al. Housing environments and child health conditions among recent Mexican immigrant families: a population-based study. J Immigr Minor Health. 2010;12(5):617-25.

17. Mangrio E, Zdravkovic S. Crowded living and its association with mental ill-health among recently-arrived migrants in Sweden: a quantitative study. BMC Res Notes. 2018;11(1):609.

18. Marchi M, Magarini FM, Chiarenza A, Galeazzi GM, Paloma V, Garrido R, et al. Experience of discrimination during COVID-19 pandemic: the impact of public health measures and psychological distress among refugees and other migrants in Europe. BMC Public Health. 2022;22(1):942.

19. Martino E, Li Y, Kali-Opio J, Bentley R. Between liminality and a new life in Australia: What is the effect of precarious housing on the mental health of humanitarian migrants? Cities. 2022;131:103900.

20. Mendola D, Busetta A. Health and Living Conditions of Refugees and Asylum-seekers: A Survey of Informal Settlements in Italy. Refugee Survey Quarterly. 2018;37(4):477-505.

21. Mohsenpour A, Biddle L, Bozorgmehr K. Exploring contextual effects of post-migration housing environment on mental health of asylum seekers and refugees: A cross-sectional, population-based, multi-level analysis in a German federal state. PLOS Glob Public Health. 2023;3(12):e0001755.

22. Mohsenpour A, Dudek V, Bozorgmehr K, Biddle L, Razum O, Sauzet O. Type of Refugee Accommodation and Health of Residents: A Cross-Sectional, Population-Based Cluster Analysis in South-West Germany. Int J Public Health. 2023;68:1605786.

23. Montazer S. Immigration, Homeownership, and Mental Health. Socius. 2022;8:23780231221139361.

24. Mora DC, Quandt SA, Chen H, Arcury TA. Associations of Poor Housing with Mental Health Among North Carolina Latino Migrant Farmworkers. J Agromedicine. 2016;21(4):327-34.

25. Oudin A, Richter JC, Taj T, Al-Nahar L, Jakobsson K. Poor housing conditions in association with child health in a disadvantaged immigrant population: a cross-sectional study in Rosengård, Malmö, Sweden. BMJ Open. 2016;6(1):e007979.

26. Raphael E, White JS, Li X, Cederin K, Glymour MM, Sundquist K, et al. Neighborhood Deprivation and Mental Health Among Immigrants to Sweden. Epidemiology. 2020;31(3):e25-e7.

27. Richter JC, Jakobsson K, Taj T, Oudin A. High burden of atopy in immigrant families in substandard apartments in Sweden - on the contribution of bad housing to poor health in vulnerable populations. World Allergy Organ J. 2018;11(1):9.

28. Sandberg JC, Talton JW, Quandt SA, Chen H, Weir M, Doumani WR, et al. Association between housing quality and individual health characteristics on sleep quality among Latino farmworkers. J Immigr Minor Health. 2014;16(2):265-72.

29. Song SJ, Kaplan C, Tol WA, Subica A, de Jong J. Psychological distress in torture survivors: pre- and post-migration risk factors in a US sample. Soc Psychiatry Psychiatr Epidemiol. 2015;50(4):549-60.

30. Song SJ, Subica A, Kaplan C, Tol W, de Jong J. Predicting the Mental Health and Functioning of Torture Survivors. J Nerv Ment Dis. 2018;206(1):33-9.

31. Srirangson A, Thavorn K, Moon M, Noh S. Mental health problems in Thai immigrants in Toronto, Canada. International Journal of Culture and Mental Health. 2013;6(2):156-69.

32. Sundquist J. Living conditions and health. A population-based study of labour migrants and Latin American refugees in Sweden and those who were repatriated. Scand J Prim Health Care. 1995;13(2):128-34.

33. Tortelli A, Simon P, Lehouelleur S, Skurnik N, Richard JR, Baudin G, et al. Characteristics associated with the risk of psychosis among immigrants and their descendants in France. Brain Behav. 2021;11(5):e02096.

34. Vignier N, Moussaoui S, Marsaudon A, Wittwer J, Jusot F, Dourgnon P. Burden of infectious diseases among undocumented migrants in France: Results of the Premiers Pas survey. Frontiers in Public Health. 2022;10.

35. Walther L, Fuchs LM, Schupp J, von Scheve C. Living Conditions and the Mental Health and Well-being of Refugees: Evidence from a Large-Scale German Survey. Journal of Immigrant and Minority Health. 2020;22(5):903-13.

36. Walther L, Kröger H, Tibubos AN, Ta TMT, von Scheve C, Schupp J, et al. Psychological distress among refugees in Germany: a cross-sectional analysis of individual and contextual risk factors and potential consequences for integration using a nationally representative survey. BMJ Open. 2020;10(8):e033658.

37. Whitsett D, Sherman MF. Do resettlement variables predict psychiatric treatment outcomes in a sample of asylum-seeking survivors of torture? Int J Soc Psychiatry. 2017;63(8):674-85.

38. Wirehag M, Andersson L, Hjern A, Ascher H. Living situations among undocumented migrants in Sweden: The effects of exclusion from fundamental housing rights. International Journal of Social Welfare. 2021;30(2):239-48.
