# Supplementary material for: Housing inequalities and health outcomes among migrant and refugee populations in high-income countries: a mixed-methods systematic review
Source: BMC Public Health. 2025 Mar 22;25:1098. doi: 10.1186/s12889-025-22186-5 (PMC11929249; doi:10.1186/s12889-025-22186-5)
Supplement: Supplementary file 4 — Supplementary Material 4 [file 12889_2025_22186_MOESM4_ESM.docx]

**Supplementary Table S4.** Characteristics and summary of qualitative studies reviewed

| **Study details** | **Population and setting** | **Study design and methods** | **Study aims** | **Main themes and subthemes/ narrative description** | **Author’s conclusions and**  **reviewer’s comments** |  |
| --- | --- | --- | --- | --- | --- | --- |
| **Authors:**  Devkota et al. (1)  **Year of publication:** 2021  **Country:**  Nepal  (migration in Gulf countries and Malaysia) | **Sampling:**  This study included 40 male participants (n=25 in 4 focus groups and n=15 in interviews), divided into two categories: non-migrants and returnee migrants from Gulf countries and Malaysia. Participants were purposively selected for interviews and focus group discussions.  **Participants details:**  Participants were Nepali male workers, including both non-migrants (n=19) and returnee migrants (n=21). The returnee migrants predominantly worked in Gulf countries and Malaysia. The age of the participants ranged from 19 to 55 years.  **Setting/Context:**  This study was conducted in Madi Municipality of Chitwan, Nepal, an urban area experiencing rapid growth and a diverse population in terms of caste, ethnicity, and socioeconomic status. | Qualitative study utilising focus group discussions (FGDs) and in-depth semi-structured interviews and employing thematic analysis. | This study aimed to explore the mental health and wellbeing experiences and perceptions of Nepali male migrant in Gulf countries and Malaysia. | The study presents its findings under key themes, with only the relevant themes briefly described below:   - **Mental health status and wellbeing** - ***Stress and mental disorder*** - ***Mental health knowledge*** - ***Other illness and health problems*** - **Factors associated with mental distress and wellbeing:** - ***Accommodation and living condition:*** Participants reported poor living conditions causing tensions and feelings of insecurity, with many staying in overcrowded shared accommodations lacking basic facilities such as beds, cabinets, refrigerators, and kitchen amenities. While a few had reasonable accommodation with adequate space and facilities, some lived in temporary accommodations at work sites, often with limited amenities. Additionally, some workers faced stressful situations due to the unacceptable behaviour and aggressiveness of their roommates, leading to feelings of insecurity and disturbance. - ***Individual behaviour and lifestyle*** - ***Job nature and income status*** - ***Work environment and employment condition*** - ***Unmet expectation and economic burden*** | **Author’s conclusions:**  *“Both migrant and non-migrant workers experienced poor mental and physical health, largely aﬀected by their adverse living and working conditions, unmet familial and financial needs and unhealthy life styles. Greater compliance is needed by employers of work agreements and the promotion of labour rights for worker’s health and safety. In addition, policy interventions to raise awareness about occupational health risks and eﬀective safety training for all workers (migrant and non-migrant) are recommended.”*  **Reviewer’s comments:**  This study of Nepali migrants returning from Gulf countries and Malaysia, found that housing conditions negatively impacted their health, characterised by overcrowded shared accommodations, and a lack of basic amenities, leading to heightened stress and insecurity.  **Assessment of methodological quality:**  This study has clearly met 7/10 (70%) criteria in the critical appraisal tool. |  |
| **Authors:**  Dhanji (2)  **Year of publication:** 2010  **Country:**  Australia | **Sampling:**  This study included 12 former refugees from the Horn of Africa and Sudan (detail sourced from previously published study in 2009 by Dhanji (3))  **Participants details:**  Participants were male and female former refugees aged between 24-45 years, resettled in metropolitan Melbourne, Australia, since the 1990s.  **Setting/Context:**  The research was conducted in metropolitan Melbourne, focusing on former refugees living in areas such as Footscray, West Heidelberg, and Dandenong. | Qualitative research methods, including structured and semi-structured face-to-face interviews, ethnography, and participant observation at cultural community events and meetings. | This study aimed to explore the socio-economic integration and wellbeing of former refugees from the Horn of Africa and Sudan in Australia, focusing on accommodation, health, welfare, and community integration. | The study presents its findings under key themes, with only the relevant themes briefly described below:   - **Accommodation**: Public housing had not been upgraded or maintained. Those living in small ‘condemned’ public housing often faced overcrowding and/or infestations both of which are relevant health issues. - **Neighbourhoods:** Participants indicated how former refugees' decisions to live in densely populated refugee suburbs, driven partly by housing affordability, lead to social isolation. This isolation is directly linked to experiences of depression, as indicated by the participants' narratives. - **Health:** Lived experiences of refugees, including housing challenges and social exclusion, contribute to a range of emotional symptoms, such as sadness and loneliness, often leading to depression. - **Family reunification and health impacts** - **Community organisations and general welfare** - **Understanding cultural adaptation through adolescents’ eyes** | **Author’s conclusions:**  *“Results indicate that a lack of linkage among the social aspects has hindered the success of the resettlement programmes.”*  **Reviewer’s comments:**  The study findings demonstrated that housing inequalities among former refugees from the Horn of Africa and Sudan in Australia are closely linked to adverse physical and mental health outcomes, including challenges in accessing healthcare, increased feelings of isolation, and depression.  **Assessment of methodological quality:**  This study has clearly met 8/10 (80%) criteria in the critical appraisal tool. |  |
| **Authors:**  Dhesi et al. (4)  **Year of publication:** 2018  **Country:**  France | **Sampling:**  Purposive selection of 11 sub-camp sites within the Calais refugee camp for maximum variation covering geographical locations, origin countries, age groups, and group size.  **Participants details:**  Participants were mostly male residents of the Calais refugee camp, representing various nationalities and age groups. More specific characteristics of participants were not included in the paper. This study specifically mentions a population of 3,000 people at the time of data collection in July 2015, with 96.8% being male.  **Setting/Context:**  This study was conducted in the Calais refugee camp, located in northern France. This informal camp, known as Europe’s largest informal refugee camp at the time, was situated near an industrial site and consisted of makeshift shelters and limited facilities. | Interdisciplinary study with qualitative group interviews and field observations to assess the environmental health conditions in the Calais refugee camp. | This study aimed to conduct a holistic environmental health survey in the Calais refugee camp and examine public health conditions, with a focus on food and water safety, shelter quality, and hygiene and sanitation. | The study presents its findings under key themes briefly described below:   - **Food and water:** Inadequate food provision led to hunger and reliance on unsafe food storage practices, while the contamination of drinking water and the absence of a hot water supply posed significant health risks to the residents. - **Shelter:** Shelter was characterised by poor insulation and ventilation, which posed physical health risks to residents, and exposed them to fire hazards and harsh environmental conditions. - **Hygiene and sanitation:** Inadequate sanitation facilities in the Calais refugee camp, including a lack of handwashing facilities and widespread open defecation, contributed to the spread of diseases, and caused significant personal distress among residents. | **Author’s conclusions:**  *“Drawing upon theories of biopolitical exclusion, the paper concludes by (i) emphasising the empirical and conceptual themes that tie refugee politics and biologies together and (ii) makes a call for increased attention to makeshift camps as key sites of health exclusion in Europe and beyond.”*  **Reviewer’s comments:**  This study identified critical public health issues in the Calais refugee camp, including poor shelter quality causing physical health hazards, and insufficient hygiene and sanitation facilities resulting in disease spread and personal distress among residents.  **Assessment of methodological quality:**  This study has clearly met 7/10 (70%) criteria in the critical appraisal tool. |  |
| **Authors:**  Due et al. (5)  **Year of publication:** 2022  **Country:**  Australia | **Sampling:**  This study included 11 participants for the photovoice exercise. Purposive sampling was employed to ensure a diverse range of experiences among participants.  **Participants details:**  The participants were male and female refugees and asylum seekers from various regions, including Africa, the Middle East, and South East Asia. They were either living in rental accommodations or owned their homes.  **Setting/Context:**  Conducted in South Australia, the study focused on the experiences of refugees and asylum seekers in relation to housing and its impact on their health and well-being. | This was a qualitative study using photovoice methodology. Participants took photographs of their housing and neighbourhood, which were then discussed in interviews. The study also included surveys and interviews with a broader group of refugees, asylum seekers, and service providers. | This study aimed to explore the relationship between housing and health for refugees and asylum seekers in South Australia, particularly focusing on how housing conditions affect their physical and psychological well-being. | The study presents its findings under key themes briefly described below:   - **Housing:** - ***Gardens and outside spaces:*** This subtheme reflects on how gardens and outdoor spaces in participants' housing contribute to or detract from their sense of well-being. It includes the dual aspect of gardens as sources of relaxation and stress, depending on maintenance issues and the ability to control these spaces. - **Housing condition:** Participants expressed concerns about the maintenance of their housing, including issues such as broken appliances and structural problems, and how these affect their living conditions and overall health. - **Layout, space, and privacy:** Participants faced challenges such as heightened stress from cramped living conditions and traumatic triggers from poorly lit or noisy environments. These issues were compounded by their limited control over their living situations due to temporary visa statuses, exacerbating their sense of powerlessness and adversely affecting their psychological well-being. - **Furnishings and homemaking practices:** Participants found that personalising their living spaces with chosen furnishings greatly enhanced their sense of home and well-being. - **Neighbourhood location:** - ***Safety*:** Participants emphasised the importance of living in safe neighbourhoods, often prioritising safety over other housing attributes. Safety concerns often made the participants choose to relocate for a safer environment. However, affordability constraints limited some participants. Moreover, participants who had experienced unsafe neighbourhoods reported negative impacts on their family's health. - **Other elements of neighbourhood – green spaces and proximity to services:** Those living in neighbourhoods with accessible greenery, such as parks and open spaces, expressed greater satisfaction and a sense of healthy living. Similarly, having essential services and amenities such as pharmacies and shopping centres within walking distance was linked to quality of life. | **Author’s conclusions:**  *“The article concludes that consideration of ways to promote ontological security in housing should be a critical component of resettlement policies.”*  **Reviewer’s comments:**  The findings show poor housing conditions experienced among migrants and refugees in South Australia, particularly in terms of housing structure and maintenance, house layout and furnishing, neighbourhood safety, and access to green spaces and amenities, are closely linked to their physical and mental health outcomes.  **Assessment of methodological quality:**  This study has clearly met 9/10 (90%) criteria in the critical appraisal tool. |  |
| **Authors:**  Fozdar (6)  **Year of publication:** 2009  **Country:**  Australia | **Sampling:**  This study included over 200 participants (divided into two sub-studies) from refugee backgrounds, including those from Bosnia, Croatia, Ethiopia, Eritrea, Sudan, and Somalia.  **Participants details:**  Participants were refugees from the Horn of Africa and former Yugoslavia, including Bosnians, Croatians, Ethiopians, Eritreans, Sudanese, and Somalis. This study does not specify the exact age range but includes a wide age group from under 20 to over 50 years.  **Setting/Context:**  The research was conducted in Perth, Western Australia, focusing on refugee settlement experiences, particularly regarding mental health concerns such as depression and PTSD. | Qualitative methods, comprising questionnaires, interviews, and focus groups. This paper reports a subsection of results from two studies aimed to understand refugees' perceptions of settlement, mental health, and well-being. Inductive thematic content analytical approach was used. | This study aimed to explore the perceptions of ex-Yugoslav and Horn of Africa refugees in Perth, Western Australia, regarding their settlement experiences and the impact on their mental health, particularly in terms of depression and PTSD. | The study presents its findings under key themes, with only the relevant themes briefly described below:   - **Understandings of mental distress** - **‘Causes’ of ‘depression’:** Housing was one of the factors that was considered as a “cause” of depression. - ***Employment issues*** - ***Culture shock:*** Discrimination in the street and in the housing rental market was perceived as depressing. | **Author’s conclusions:**  “*It reports data from questionnaires, interviews and focus groups with over 200 people from refugee backgrounds- including Bosnians, Croatians, Ethiopians, Eritreans, Sudanese and Somalis-which indicate their perception that post-migration experiences are more important in undermining well-being than pre-migration physical and psychological trauma.”*  **Reviewer’s comments:**  The study findings suggest housing inequalities contribute to mental health outcomes by causing stress and depression, particularly due to experiences of discrimination in the housing market.  **Assessment of methodological quality:**  This study has clearly met 7/10 (70%) criteria in the critical appraisal tool. |  |
| **Authors:**  Hanley et al. (7)  **Year of publication:** 2019  **Country:**  Canada | **Sampling:**  This study included 26 adult newcomer women residing in Montreal, Canada. Participants were recruited through advertisements and collaboration with community organisations and had self-identified as having experienced housing insecurity.  **Participants details:**  Participants were foreign-born women who immigrated to Canada within the last ten years, and were diverse in immigration status, ethnicity, race, country of origin, family composition, sexual orientation, age, and physical and mental ability.  **Setting/Context:**  This study focused on the experiences of foreign-born women in Montreal, encompassing various forms of housing insecurity, from absolute homelessness to invisible homelessness (e.g., couch surfing). | Qualitative analysis using open-ended interviews and constant comparative method for theme exploration. | This study aimed to explore how health intersects with the experience of housing insecurity and homelessness, specifically for migrant women, and to understand the specifics of these interplays for advocacy and policy improvement. | The study presents its findings under key themes, with only the relevant themes briefly described below:   - **Health as a factor leading women to fall into housing insecurity and homelessness…:** Migrant women facing housing insecurity often struggled with various health issues, such as migraines, high blood pressure, and reproductive problems, which directly contributed to their housing instability, with no reports of substance abuse influencing their situation. - **...and health as a factor keeping them there:** Migrant women facing housing insecurity, reported that their unstable housing situations significantly exacerbated their stress and mental health issues, leading to serious physical health problems including chronic pain, vision loss, and hormonal imbalances, which in turn hindered their ability to work or improve their living conditions. - **Health challenges specific to (im)migrant women** - **Ineligibility for public health insurance** - **Fear that health will be a barrier to permanent immigration** - **Language and cultural barriers to the health system** - **Family separation, social isolation and lack of support in times of health crisis** - **Healthcare strategies** - **Advice and support from friends** - **Personal faith and determination** - **Accessing local women’s homeless shelters** | **Author’s conclusions:**  “*The findings are presented around three themes: how health problems instigate and maintain migrant women’s housing insecurity and homelessness; ways in which women’s immigration trajectories and legal status may influence their health experiences; and particular coping strategies that migrant women employ in efforts to maintain or manage their health. The authors conclude with implications of these findings for both policy and practice in relation to migrant women who experience or are at risk of housing insecurity and homelessness.”*  **Reviewer’s comments:**  This study conducted in Montreal, Canada, revealed that migrant women's pre-existing health issues such as migraines and reproductive problems lead to housing instability, while stress from this insecurity further exacerbates their health, causing chronic conditions that hinder their ability to work and better their living situations.  **Assessment of methodological quality:**  This study has clearly met 10/10 (100%) criteria in the critical appraisal tool. |  |
| **Authors:**  Haque and Rosas (8)  **Year of publication:** 2010  **Country:**  Canada | **Sampling:**  This study included 41 immigrant residents using purposive sampling method for recruitment.  **Participants details:**  Immigrant residents, aged 18 to 65 years, having lived in Canada for less than 10 years and in St. James Town for at least 6 months.  **Setting/Context:**  This study was conducted in St. James Town, a densely populated, low-income urban neighbourhood in Toronto, Canada, with a significant immigrant population, comprising around 64% of residents. The neighbourhood is characterised by high-rise rental apartment buildings and is home to a diverse array of ethnoracial communities, with over 50 languages spoken. | This study employed a community-based participatory research (CBPR) design, integrating photovoice to capture immigrant resident’s perceptions through photographs and stories, followed by concept mapping to organise and build consensus around neighbourhood factors influencing health and well-being. | This study aimed to explore the health and well-being of immigrant residents through participatory research methods such as photovoice and concept mapping. | The study findings are described below:   - This study identified assets such as green spaces, attractions, societal connections, education, and the need for social support, alongside liabilities including a lack of maintenance, health improvement challenges, environmental issues, and safety concerns, all impacting the health and well-being of immigrant residents in the urban neighbourhood. - A conceptual map was created delineating interconnected factors affecting health in the community. Stress value from multidimensional scaling (MDS) was 0.27, indicating a good fit. | **Author’s conclusions:**  *“The combination of these 2 participatory methods resulted in a conceptual framework of factors influencing im- migrants’ health and well-being, whereas the photographs with captions facilitated interpretation and action at multiple levels.”*  **Reviewer’s comments:**  In this study, neighbourhood factors were organised into a framework showing how different elements are interconnected and influence health, particularly safety, physical and hygienic conditions, lack of green and recreational spaces, poor maintenance, or inadequate social assets/support were linked with negative health outcomes. In the concept map, lack of maintenance and improvement formed the largest cluster.  **Assessment of methodological quality:**  This study has clearly met 7/10 (70%) criteria in the critical appraisal tool. |  |
| **Authors:**  Hashimoto-Govindasamy and Rose (9)  **Year of publication:** 2011  **Country:**  Australia | **Sampling:**  The study included 12 female Sudanese participants from refugee backgrounds. Participants were self-referred or referred by other services to the program.  **Participants details:**  Participants were female refugees from the Sudanese background, aged 25-41 years, all with children. The majority spoke Dinka, with one speaking Nuer and one Madi.  **Setting/Context:**  This study was conducted at the Mamre Homestead in St Marys, a suburb in the Penrith Local Government Area of Sydney, known for its significant socioeconomic disadvantage. The program was an 8-week exercise and education initiative for Sudanese refugee women in western Sydney, including activities such as walking, dancing, and language learning, aimed at aiding their resettlement and well-being. | This was an ethnographic process evaluation of a community support program, involving participant observation (using the Participant as Observer method) and a group interview to gain insight into the Sudanese women’s experiences. | This study aimed to explore Sudanese refugee women’s perceptions of the community support program and their ongoing resettlement needs for future service development. | The study presents its findings under key themes, with only the relevant themes briefly described below:   - **Perceived impact of the program** - **Post migration issues and needs:** Housing difficulties, particularly finding house and discrimination, were among the stressors faced by the participants, which had an impact on their mental health during resettlement. | **Author’s conclusions:**  *“It is vital that any attempts to address these issues are sustainable, aiming to empower the women and promote their existing strengths and resilience techniques. Research specific to cultural and ethnic groups of refugee women in an Australian context enables tailoring of appropriate support services, but can be tiresome for participants.”*  **Reviewer’s comments:**  The study findings indicate that housing difficulties are among the stressors faced by the Sudanese refugee women, which can impact their mental health during resettlement.  **Assessment of methodological quality:**  This study has clearly met 9/10 (90%) criteria in the critical appraisal tool. |  |
| **Authors:**  Holmes (10)  **Year of publication:** 2006  **Country:**  US | **Sampling:**  More than 130 farm workers and 30 clinicians.  **Participants details:**  Indigenous Triqui Mexicans working as migrant farm laborers in the western United States, along with clinicians serving this population.  **Setting/Context:**  This ethnographic study followed the Triqui Mexicans through their migration in the western US and Mexico, working in farms and interacting with health clinics. This study explored the social context of migrant health, focusing on labour conditions, housing, ethnicity, and healthcare. Interviews were conducted on farms and in clinics over 15 months of migration. | Qualitative study employing participant observation and interviews, integrating grounded theory with theories of structural violence, symbolic violence, and the clinical gaze for data analysis. | This study aimed to identify how the social context of migrant farm workers, particularly indigenous Mexicans in the US, affects their health and healthcare which includes an examination of the structural and symbolic dynamics influencing their working, living conditions, and the healthcare they receive. | The study presents its findings under key themes, with only the relevant themes briefly described below:   - **Ethnicity and citizenship hierarchies in farm labour:** Indigenous Mexican migrants, particularly the undocumented migrants, face the poorest living conditions. These conditions include overcrowding, inadequate shelter, lack of basic facilities, and exposure to environmental hazards. - **Health disparities and health care:** Increased exposure to environmental hazards, such as pesticides, lead to higher rates of occupational injuries and chronic health problems such as respiratory issues and skin conditions. Lack of sanitation and proper nutrition due to inadequate living facilities, contribute to gastrointestinal diseases and nutritional deficiencies. General exposure to harsh living conditions lead to a higher incidence of communicable diseases. - **Abelino: work injury and chronic pain** - **Crescencio: Somatization and substance use** - **Bernardo: trauma and political violence** - **Racism, naturalisation, and internalisation:** Stress and anxiety resulting from insecure, inadequate, and unsafe living conditions were noted. Social isolation and lack of privacy, contributed to feelings of depression and hopelessness. The internalisation of their marginalised status, as the Triqui workers tend to accept these conditions as a norm, can exacerbate feelings of helplessness and low self-worth. | **Author’s conclusions:**  *“Structural racism and anti-immigrant practices determine the poor working conditions, living conditions, and health of migrant workers. Subtle racism serves to reduce awareness of this social context for all involved, including clinicians. The paper concludes with strategies toward improving migrant health in four areas: health disparities research, clinical interactions with migrant laborers, medical education, and policy making.”*  **Reviewer’s comments:**  This study revealed how housing inequalities among Triqui migrant farm workers in the United States are directly linked to detrimental physical and mental health outcomes, stemming from poor living conditions, exposure to environmental hazards, and exacerbated by the intertwined challenges of work and social marginalisation.  **Assessment of methodological quality:**  This study has clearly met 10/10 (100%) criteria in the critical appraisal tool. |  |
| **Authors:**  Howden-Chapman et al. (11)  **Year of publication:** 2000  **Country:**  New Zealand | **Sampling:**  This study included six focus groups facilitated by five Tokelau people. Participants were selected through the Wellington Tokelau Association.  **Participants details:**  This study included members of the Tokelau community in Wellington, New Zealand, comprising a range of age groups and both genders, including older adults, middle-aged adults, young people, and community workers.  **Setting/Context:**  The research was conducted within the Wellington Tokelau community, a small Pacific Islander community migrating to New Zealand. | Qualitative study employing focus group discussions and integrating grounded theory for data analysis. | This study aimed to understand the relationship between crowded homes and health within the Tokelau community, focusing on cultural patterns and the impact of economic factors on housing decisions. | The study presents its findings under key themes, with only the relevant themes briefly described below:   - **View of health** - **Life in the Tokelau Islands compared with life in New Zealand** - **Social patterns:**   Prior to coming to mainland New Zealand, house inspections by women and nurses in the community were regular to ensure housing standards, whereas upon arrival, these inspections were absent.   - **Housing in Tokelau** - **Housing and health problems:** Participants noted challenges of overcrowding in small houses, leading to health issues such as asthma due to inadequate ventilation and social problems including strained relationships and lack of privacy. Traditional Tokelau hospitality norms, which encourage living as an extended family, clash with the realities of New Zealand's housing, resulting in both physical and social stressors within the community. - **Household budgets** - **Smoking** - **Renting versus buying** - **Possible solutions** | **Author’s conclusions:**  *“While cultural patterns were an essential part of Tokelau hospitality, the decision to “double up” households was often the result of “rational” economic decision making in relation to household expenditures such as rent and food. The implication for public health practitioners is that while overcrowding may be a health hazard for residents, the most effective solutions by the community are higher household income and more flexible housing designs that accommodate multifamily households.”*  **Reviewer’s comments:**  This study revealed that among the Tokelau community residing in New Zealand, the traditional practice of living as an extended family often leads to overcrowded and poorly insulated homes, which exacerbates health issues such as asthma and may also lead to social tensions. The discrepancy in housing inspections further highlights the higher level of care and community expectations in the previous environment compared to the care provided after arriving in mainland New Zealand.  **Assessment of methodological quality:**  This study has clearly met 8/10 (80%) criteria in the critical appraisal tool. |  |
| **Authors:**  Keim-Malpass et al. (12)  **Year of publication:** 2015  **Country:**  US | **Sampling:**  This study involved 30 immigrant farmworkers recruited across North Carolina.  **Participants details:**  This study included adult farmworkers or adult partners living with a farmworker and able to speak Spanish or English fluently. Participants included both unaccompanied men with and without H2-A visas and members of migrant families in small and large camps, as well as seasonal farmworker families.  **Setting/Context:**  The research was part of a broader community-based participatory research aimed at documenting housing quality and health among migrant farmworkers in North Carolina. | Qualitative study employing semi-structured interviews and photographs were used to document housing quality, and content analysis was conducted for data analysis. | This study aimed to explore the perceptions of housing conditions among migrant farmworkers in rural North Carolina and develop an understanding of potential impacts of their housing on health and safety. | The study presents its findings under key themes briefly described below:   - **Exposure to pesticides:** Farmworkers expressed concerns about pesticides due to the close proximity of their housing to agricultural fields. This exposure led to symptoms such as headaches and feelings of nausea. - **Safety issues:** Participants highlighted safety concerns related to housing, including faulty utilities and electricity, which posed risks of accidents and injury. - **Pests:** The presence of pests, such as cockroaches, mice, and rats, was a significant concern, often due to structural issues including holes and rotting floors. These pest infestations raised concerns about disease transmission and contributed to unsanitary living conditions. - **Water supply:** Participants reported issues with water quality, including bad taste and visible dirt, leading to gastrointestinal discomfort and limiting water consumption. Some participants also reported that they had to use dirty water for household purposes, highlighting concerns about hygiene and health. - **Air quality, temperature and moisture:** Participants described problems with air quality, inadequate heating and cooling systems, and issues related to moisture and mould. | **Author’s conclusions:**  *“This study describes migrant farmworkers’ perceptions of housing quality and numerous potential impacts on health and safety. Research, social policy and practice-based implications derived from this research could serve to improve the health status of these individuals and their families. This study suggests there is much room for sustained advocacy and action, given that many of the farmworkers’ descriptions and photographs depicted housing conditions below accepted standards of living. Access to adequate and safe employer-provided housing for migrant farmworkers should be considered a basic human right.”*  **Reviewer’s comments:**  This study focused on immigrant farmworkers in North Carolina identified key housing factors contributing to health concerns stemming from exposure to pesticides, safety hazards due to faulty utilities, pest infestations, poor water quality, and issues with air quality, temperature, and moisture in their housing.  **Assessment of methodological quality:**  This study has clearly met 10/10 (100%) criteria in the critical appraisal tool. |  |
| **Authors:**  Khan et al. (13)  **Year of publication:** 2022  **Country:**  Canada | **Sampling:**  This study included 6 participants who were purposively sampled from homeless youth clients at a youth emergency residence using eligibility criteria based on a broad definition of refugees and refugee claimants.  **Participants details:**  The sample consisted homeless refugee youth aged 16–24 years (mean age 21.3 years), with two-thirds unaccompanied by family and residing in Canada for an average of 3.7 years.  **Setting/Context:**  This study was conducted in private offices at Youth Without Shelter (YWS) or Centre for Addiction and Mental Health in Toronto, Canada, based on participant preference, with mental health support available on-site. | Qualitative study using semi-structured interviews analysed with thematic analysis. | This study aimed to explore the mental health needs and resilience factors of refugee youth residing at youth emergency residence. | The study presents its findings under key themes, with only the relevant themes briefly described below:   - **System-level factors impacting mental health:** - ***Immigration process*** - ***Housing insecurity:*** Housing insecurity was a major challenge for refugee youth, with some experiencing fear and stress in shelters, while others struggled with adjusting to shared accommodations and concerns about their immigration status. However, YWS offered housing interventions, including support with renting, life skills, and an after-care program, which were perceived as beneficial for improving mental wellbeing and facilitating the transition to independent living. - ***Financial*** - ***Education*** - ***Employment*** - ***Sense of safety*** - **Acculturative stress, a product of transition to Canada:** - ***Language*** - ***Culture shock*** - **Fostering a sense of control:** - ***Goal-directedness*** - ***Independence and responsibility*** - **Social connectedness and belonging:** - ***Family*** - ***Peer*** - ***Sense of community*** | **Author’s conclusions:**  *“A model for planning future interventions was proposed and was informed by the lived experience of participants. This model focuses on initially on immediate and basic needs and reflected evidence gathered through this work to attend to long-term needs once individuals have integrated into society. Future efforts will be directed toward translating the lived experience of this population and developing practice guidelines to optimize care.”*  **Reviewer’s comments:**  The study findings indicate that housing insecurity was a significant challenge for refugee youth, but housing interventions, including rental support, life skills, and after-care programs helped improve mental wellbeing.  **Assessment of methodological quality:**  This study has clearly met 8/10 (80%) criteria in the critical appraisal tool. |  |
| **Authors:**  Lauritzen and Sivertsen (14)  **Year of publication:** 2012  **Country:**  Norway | **Sampling:**  This study involved 11 focus group interviews with 34 staff at asylum processing centres.  **Participants details:**  This study uses insights from professionals working with children and their families’ seeking asylum, living at governmental asylum processing centres in Northern Norway.  **Setting/Context:**  The research was conducted in various governmental asylum processing centres in Northern Norway, focusing on the living conditions and mental health of children and families seeking asylum. | Qualitative study with focus group interviews with staff members at asylum processing centres. | This study aimed to identify central environmental conditions affecting the mental health of children living with their families at governmental asylum processing centres in Northern Norway. | The study presents its findings under key themes, with only the relevant themes briefly described below:   - **Life at the asylum centre:** - ***Time*:** Extended periods of stay in these centres exacerbate stress and anxiety, impacting both physical and mental well-being. - ***Parents’ helplessness*** - ***Parents’ health:*** The mental health of parents, influenced by their living conditions in the asylum centres, directly affects the mental health of their children. - ***The competence of the centre workers*** - ***Housing standards:*** Inadequate living conditions, contributes significantly to mental health issues among asylum-seeking children and their families. - ***Schooling and activities:*** Limited access to educational and recreational activities due to housing inequalities can affect the mental health of children. Lack of engagement in meaningful activities contributes to feelings of hopelessness and lack of purpose, impacting both mental and physical health. - **The centre and the community:** - ***Isolation and Segregation:*** Segregated and isolated asylum centres contribute to social exclusion, and limits interactions with the local community, exacerbating feelings of isolation and alienation, leading to mental health issues such as depression and anxiety. - ***Poverty*** - **Global perspectives:** - ***International policies and events*** - ***National policies and events*** - ***Basic rights*** | **Author’s conclusions:**  *“The findings suggest that these children are indeed vulnerable, and at high risk of developing mental health problems. Their rights are, however, open to local interpretations, and they fall between two stools; their right to proper health care, and national and inter- national immigration policies.”*  **Reviewer’s comments:**  This study revealed that prolonged stays in inadequate asylum centres, marked by poor living conditions, social isolation, and poverty, significantly deteriorate the physical and mental health of asylum-seeking families in Northern Norway.  **Assessment of methodological quality:**  This study has clearly met 7/10 (70%) criteria in the critical appraisal tool. |  |
| **Authors:**  Miller et al. (15)  **Year of publication:** 2002  **Country:**  US | **Sampling:**  This study included 28 refugee adults from Bosnia living in Chicago, and convenience sampling was employed.  **Participants details:**  Participants were from the Bosnian background, with diversity in sociodemographic characteristics.  **Setting/Context:**  Participants were either in the Bosnian Mental Health Program or its sister program, the Refugee Mental Health Program, in Chicago. Clients were invited to participate based on consecutive admission to the respective programs over the previous eight months. | Qualitative, exploratory study with convenience sampling, with in-depth interviews and thematic analysis. | This study aimed to explore the stressors associated with the experience of exile among a sample of Bosnian refugees living in Chicago. | The study presents its findings under key themes, with only the relevant themes briefly described below:   - **Social isolation and the loss of community** - **The loss of life projects** - **A lack of environmental mastery** - **The loss of social roles and the corresponding loss of meaningful activity** - **A lack of sufficient income for adequate housing and other basic necessities:** Lack of sufficient income for adequate housing was the single most common source of exile-related distress among participants. Moreover, participants noted factors included overcrowding causing privacy issues and stress. - **Health problems not previously experienced in Bosnia** | **Author’s conclusions:**  *“Primary sources of exile-related distress included social isolation and the loss of community, separation from family members, the loss of important life projects, a lack of environmental mastery, poverty and related stressors such as inadequate housing, and the loss of valued social roles. The implications of these findings for mental health interventions with refugees are considered, and the value of narrative methods in research with refugee communities is discussed.”*  **Reviewer’s comments:**  The study findings revealed that the lack of sufficient income for adequate housing was the most common source of distress related to social isolation among Bosnian refugees in Chicago.  **Assessment of methodological quality:**  This study has clearly met 7/10 (70%) criteria in the critical appraisal tool. |  |
| **Authors:**  Mwanri et al. (16)  **Year of publication:** 2022  **Country:**  Australia | **Sampling:**  Out of this broader study, the current paper presents the perspectives and experiences of 20 African migrants in South Australia regarding the post-migration stressors affecting mental health. Snowball sampling was used to recruit these participants.  **Participants details:**  Participants were African migrants living in South Australia, aged 18 to 60 years, from eight different African countries. They included immigrants and refugees, with a duration of stay in Australia ranging from 3 to 20 years.  **Setting/Context:**  This study was conducted in South Australia to explore post-migration stressors affecting mental health among African migrants. | Qualitative study through one-on-one online interviews. The framework analysis method guided the data analysis process. | This study aimed to explore the mental health stressors, access to mental health services, and ways to improve mental health services for African migrant populations in South Australia. | The study presents its findings under key themes, with only the relevant themes briefly described below:   - **Family-related factors:** - ***Family disconnection*** - ***Spousal relationships and children related issues*** - **Cultural parenting practices** - **Economic factors:** Participants stated owning a home was seen as a significant achievement for successful resettlement among African migrants in Australia. However, the responsibility of a mortgage brought considerable psychological stress. A notable example was a 54-year-old man, a resident in Australia for 16 years, who experienced intense fear and sleeplessness caused by the stress of repaying his mortgage. This stress was primarily due to his worries about finding ways to manage the loan repayment. - **Community strengths** | **Author’s conclusions:**  *“The findings indicate the need for policy and intervention programs that address the above challenges. The provision of interventions, including social support such as subsidized or free childcare services, could help leverage their time and scheduled paid employment, creating time for effective parenting and improving their mental health and wellbeing. Future studies exploring what needs to be achieved by government and non-governmental institutions to support enhanced access to social and employment opportunities for the African migrant population are also recommended.”*  **Reviewer’s comments:**  In this study, owning a home was perceived as a key achievement for resettled African migrants in Australia, yet the accompanying mortgage responsibilities led to severe psychological stress.  **Assessment of methodological quality:**  This study has clearly met 9/10 (90%) criteria in the critical appraisal tool. |  |
| **Authors:**  Palmer and Ward (17)  **Year of publication:** 2007  **Country:**  UK | **Sampling:**  This study included 21 refugees and asylum seekers who were currently residing in London. Recruitment was via a refugee service – all participants were using the service. Maximum variation sampling with maximal differences in nationality, religion, culture, current location in London, age, class and immigration status.  **Participants details:**  Diverse group of refugees and asylum seekers in London, varying in nationality, religion, culture, age, class, and immigration status.  **Setting/Context:**  This study was conducted in London, UK. | Qualitative study conducted using in-depth, semi-structured interviews. Thematic analysis was employed to identify key themes from the interviews. | This study aimed to explore the mental health needs and understand the experiences and perceptions of asylum seekers and refugees in London regarding mental health care. | The study presents its findings under key themes, with only the relevant themes briefly described below:   - **Social issues and mental ill-health** - ***Housing*:** The respondents experienced stress, depression, and other mental health issues due to poor housing conditions such as overcrowding, lack of privacy, inadequate facilities, and frequent moves. This study also suggests that temporary accommodations, such as B&Bs and hostels, negatively impacted mental health. Furthermore, housing deprivation in ethnic minority and refugee communities was associated with poorer overall health, including lower resistance to mental and physical illness and impacts on behavioural and psychological well-being. - ***Immigration*** - ***Employment and other issues*** - ***Referrals, access and waiting time*** - ***Conceptions of mental health*** - ***Confidentiality and stigma*** - ***Alleviating distress*** - ***Emotional and practical support*** - ***Talking therapies*** | **Author’s conclusions:**  *“The implications highlight a need to shift from a simple biomedical model of the causes and effects of ill-health to a social model, which will require reorganisation not only in healthcare but in welfare, housing, employment and immigration policy.”*  **Reviewer’s comments:**  The study findings suggest that housing inequalities among migrants and refugees in London can exacerbate mental health issues, as inadequate, poor quality or unstable housing emerges as a significant stressor in their experiences.  **Assessment of methodological quality:**  This study has clearly met 10/10 (100%) criteria in the critical appraisal tool. |  |
| **Authors:**  Papadopoulos et al. (18)  **Year of publication:** 2004  **Country:**  UK | **Sampling:**  Semi-structured interviews and a semi-structured questionnaire with 106 Ethiopian participants were employed– 98 lay participants and 8 expert participants (Ethiopians providing professional services for Ethiopian refugees) identified through quota and snowball sampling.  **Participants details:**  Participants were Ethiopian refugees and asylum seekers residing in the UK with various sex, age, and sociodemographic backgrounds, with a focus on those who had experienced migration, adaptation, and settlement in the UK.  **Setting/Context:**  This study was conducted in various locations across the UK, primarily in urban settings where Ethiopian communities resided. | Qualitative study conducted using in-depth, semi-structured interviews and a semi-structured questionnaire which was embedded in multi-method participatory modelled study. | This study aimed to explore the experiences of Ethiopian refugees in the UK in terms of migration, adaptation, and settlement. | The study presents its findings under key themes, with only the relevant themes briefly described below:  **Migration, adaptation and settlement:**   - **Fleeing Ethiopia** - **Adapting to the UK culture and systems** - **Settling in the UK:** - ***Experiences with the UK immigration department*** - ***Experiences with employment*** - ***Educational attainment*** - ***Experiences with housing:*** Common problems included lack of space, lack of privacy, poor building conditions, noisy or hostile neighbours, insecure tenancy, and undesirable location. Safety concerns were also raised, particularly by female participants who feared unannounced visits from landlords. - ***Material security:*** Financial constraints associated with housing also impacted the ability to maintain a healthy lifestyle, including access to nutritious food and healthcare services. - ***Experiences with statutory social welfare services*** - ***Social support***   **Health and sickness beliefs and practices:**   - **Health and sickness beliefs:** Housing conditions such as overcrowding, lack of privacy, and poor living conditions can lead to stress and mental health issues. - ***Specific beliefs related to mental health and mental illness*** - **Experiences of ill health** - **Help seeking and coping with ill health** - ***Coping with mental health problems*** - **Comparisons between healthcare practices in Ethiopia and the UK** | **Author’s conclusions:**  *“Migration, adaptation and settlement experiences impact on the health of refugees and are dependent on a number of barriers and enablers, both at a personal and societal level. These should be taken into account in the provision of health and social care services, in particular services should be provided in a culturally competent manner.”*  **Reviewer’s comments:**  The findings from this study suggest that among Ethiopian refugees in the UK, housing inequalities contribute to adverse physical and mental health outcomes, primarily through factors such as inadequate living conditions, and barriers to accessing healthcare services.  **Assessment of methodological quality:**  This study has clearly met 10/10 (100%) criteria in the critical appraisal tool. |  |
| **Authors:**  Rast et al. (19)  **Year of publication:** 2024  **Country:**  Germany | **Sampling:**  This study included 47 participants (of which 42 were asylum seeker and refugee) recruited from various contexts, including outpatient clinics in reception centres (i.e. initial temporary accommodation facility), language learning activities, and psychosocial centres facilitated by healthcare professionals and researchers to ensure diverse representation.  **Participants details:**  This study included participants aged 19-65 year with a majority of asylum seekers from diverse nationalities, including European, African, and Asian countries. Participant legal statuses ranged from asylum seekers to individuals with refugee status or tolerated stay.  **Setting/Context:**  The research was conducted in different reception centre settings and language learning activities organised by non-governmental organisations in Germany between 2017-2020. | Secondary qualitative content analysis was conducted to systematically extract and analyse data, focusing on the material, meaningful, community, spatial-geographic, and socioeconomic dimensions of housing. Interviews were coded using an iterative framework combining theory-driven categories and inductive sub-coding to identify themes. | This study aimed to explore the lived experiences of asylum seekers and refugees in relation to housing and health. | The study presents its findings under key themes which are briefly described below:   - **Material conditions in reception centres create and exacerbate health issues:** The overcrowded and poorly maintained conditions in reception centres, including shared spaces with strangers, disrupted sleep, lack of privacy, noise, and hygiene issues, were major stressors that negatively impacted residents’ physical and mental health. Participants reported heightened vulnerability to illness, difficulty studying or processing emotions, and feelings of insecurity due to inadequate room locks and staff access. - **Unstable housing impedes integration and disrupts social and professional support network:** Life in reception centres is marked by uncertainty and frequent relocations, disrupting stability, integration, medical care continuity, and support networks, while straining residents’ mental health and psychosocial well-being. - **"Homemaking" is constrained in reception centres, leading to worsened mental health:** Life in reception centres is marked by restricted autonomy, shared and crowded living spaces, limited privacy, and institutional control, contrasting with the improved well-being, privacy, and independence experienced by those who transition to smaller accommodation. - **Reception centres do not make sufficient accommodations for individuals with specific medical needs:** Reception centres often fail to meet the specific health-related accommodation needs of individuals, such as accessibility, dietary requirements, and privacy, leading to physical health risks and heightened stress. | **Author’s conclusions:**  *"This study offers a broad perspective on the relationship between ASR reception centres and health. It is evident that this housing context is not conducive to health. Instead, centralised accommodation facilities exacerbate existing health conditions through excessive overcrowding, insufficient infrastructure and the inability to meet health-related needs. Stressful living environments and frequent transfers further burden psychosocial health by impeding a sense of belonging and comfort and by disrupting social and care networks. Current accommodation policy and practice need to be reconsidered to safeguard the health of ASR."*  **Reviewer’s comments:**  The study findings indicate that the material conditions, unstable housing, restricted autonomy, and lack of accommodations for specific medical needs in reception centres exacerbate health issues, hinder integration, disrupt support networks, and negatively impact resident's physical and mental well-being.  **Assessment of methodological quality:**  This study has clearly met 9/10 (90%) criteria in the critical appraisal tool. |  |
| **Authors:**  Regmi et al. (20)  **Year of publication:** 2020  **Country:**  Nepal  (migration in Oman, Saudi Arabia, Qatar and Malaysia) | **Sampling:**  There were 4 FGDs with returnee migrants (male-3 groups, female-1 group), and 1 FGD with aspiring migrants. There were 7 in-depth interviews with aspiring migrants who attended pre-departure training, and 8 semi-structured key informant interviews with stakeholders working for migrants.    **Participants details:**  Participants were Nepali migrant workers, both male and female, who had worked abroad for at least 6 months as labour migrants. The age range of participants varied. They were recruited from Tribhuvan International Airport, local hotels near the airport, organisations working for migrants, and through migrants' networks.  **Setting/Context:**  The study was conducted in Kathmandu, Nepal. It focused on exploring factors affecting the mental health of Nepali migrant workers (in Oman, Saudi Arabia, Qatar and Malaysia) and their perceptions of pre-departure training programs. | Qualitative study conducted using in-depth, semi-structured interviews as well as focus group and thematic analysis. | This study aimed to identify triggers of mental ill-health among Nepali migrant workers and their perceptions on the need for mental health components in the pre-departure orientation program. | The study presents its findings under key themes, with only the relevant themes briefly described below:   - **Factors affecting mental health and wellbeing:** - ***Families as a source of poor mental health*** - ***Unfair treatment at work*** - ***Poor arrangement of accommodation abroad:*** Inadequate living conditions abroad, including security and hygiene concerns, negatively impacted mental health. - ***Poor social life abroad*** - ***Loneliness and insecurity*** - **Perceptions on pre-departure orientation** - **Using general and mental health services abroad** | **Author’s conclusions:**  *“We found little on mental health in the pre-departure orientation. We need to improve our knowledge of mental health risks to provide better, more focused and more up-to-date pre-departure training to new migrant workers leaving Nepal.”*  **Reviewer’s comments:**  The study findings highlight mental health challenges faced by Nepali migrant workers abroad (migration in Oman, Saudi Arabia, Qatar and Malaysia) due to factors such as housing inequalities, and demonstrate impact of poor living conditions on mental health outcomes.  **Assessment of methodological quality:**  This study has clearly met 8/10 (80%) criteria in the critical appraisal tool. |  |
| **Authors:**  Rzepka et al. (21)  **Year of publication:** 2022  **Country:**  Germany | **Sampling:**  In this study, 14 refugees with symptoms of PTSD were interviewed.  **Participants details:**  Asylum seekers or those with temporary residence permits, aged 18 or older were included. All participants had previously been identified as experiencing PTSD through the process of a previous study.  **Setting/Context:**  This study took place in temporary shelters within the Rhine-Neckar district. | A qualitative study using semi-structured interviews and analysed using qualitative inductive analysis. | This study aimed to explore the relationships between the effects of traumatic events and the challenges faced by refugees in their host country. | The study presents its findings under key themes, with only the relevant themes briefly described below:   - **Traumatic events** - **Dealing with traumatic experiences and following symptoms** - **Changing views due to traumatic experiences** - **Current stress factors:** Uncertainty of the asylum process and insecure living conditions are significant stress factors for refugees. - **Experiences with integration in Germany:** The challenges refugees face in integrating into a new society includes finding stable housing. The struggle to integrate, influenced by their temporary housing situation, impacts their mental health. | **Author’s conclusions:**  *“The interactions between the post-traumatic symptoms and the living conditions of the refugees were highlighted. The effects of the symptomatology of trauma sequelae and the framework conditions under which refugees live can lead to aggravated psychological distress. Therefore, special attention must be paid to refugee mental health care.”*  **Reviewer’s comments:**  This study highlights the uncertainty of the asylum process and insecure living conditions, along with the challenges of integrating into German society and securing stable housing, which contribute to stress and impact the mental health of refugees experiencing PTSD and residing in Germany.  **Assessment of methodological quality:**  This study has clearly met 8/10 (80%) criteria in the critical appraisal tool. |  |
| **Authors:**  Sah et al. (22)  **Year of publication:** 2019  **Country:**  UK | **Sampling:**  This study involved in-depth interviews with 20 older Nepalese women. The sampling technique was purposive, targeting a specific demographic group within a defined geographical area.  **Participants details:**  The participants were older Nepalese women, primarily from rural backgrounds in Nepal, who had migrated to the UK. Most were in their 60s and 70s, with varying marital statuses (widowed and married), and many had chronic health conditions.  **Setting/Context:**  The study was conducted in the London Borough of Greenwich, focusing on the experiences of older Nepalese migrant women in an urban setting in the UK. | Qualitative research design with in-depth interviews conducted in Nepali, translated, and transcribed into English. Grounded thematic analysis was used. | This study aimed to explore and understand the factors driving mental distress among older Nepalese women living in the UK. | The study presents its findings under key themes, with only the relevant themes briefly described below:   - **Absence of family** - **Language barriers** - **Housing problems:** Participants faced struggles with poor housing quality and affordability, which negatively impacted their living conditions and food choices. Poor housing conditions, overcrowding, and restrictions on facility use significantly impacted the eating behaviours of these older women, with many reporting being unable to cook at their preferred times and sometimes going to sleep without a meal. Participants also noted lack of power over housing was a source of distress. - **Physical illness and absence of appropriate support** - **Fears of death** - **Inadequate financial resources** | **Author’s conclusions:**  *“Findings highlight that re-settlement in the absence of family is at the heart of emotional challenges for older Nepalese women. The paper concludes with a series of recommendations for supporting processes of settlement to mitigate this risk among older Nepalese women in the UK.”*  **Reviewer’s comments:**  This study revealed that older Nepalese migrant women in an urban setting in the UK faced challenges with poor housing quality and affordability, negatively impacting their living conditions. Poor housing conditions and overcrowding also impacted their eating behaviours due to limited cooking/kitchen access, and they also experienced distress due to their lack of control over housing.  **Assessment of methodological quality:**  This study has clearly met 9/10 (90%) criteria in the critical appraisal tool. |  |
| **Authors:**  Smith et al. (23)  **Year of publication:** 2019  **Country:** Australia | **Sampling:**  This study involved 6 semi-structured face-to-face focus groups (24 participants) and 7 individual interviews.  **Participants details:**  The participants were adult and youth former refugees from Afghanistan, Bhutan, Burma, Sierra Leone, Sudan, and Iran, as well as essential service providers residing in Launceston, Tasmania.  **Setting/Context:**  This study was conducted in Launceston, Tasmania, focusing on the resettlement experiences of former refugees in a regional Australian context. | Qualitative research design using in-person interviews and focus groups and employing phenomenological approach. | This study aimed to examine the resettlement experiences of former refugees in regional Australia, focusing specifically on mental health and access to mental health and support services, including barriers to access. | The study presents its findings under key themes, with only the relevant themes briefly described below:   - **Mental health of former refugees:** Challenges in securing housing were identified as significant sources of stress. - **Barriers to accessing mental health services:** - ***English language and interpreters*** - ***Culturally informed practices*** - ***Trauma‐informed care*** | **Author’s conclusions:**  *“Culturally sensitive, trauma‐informed and discrimination‐free practices should be employed across services, where Western‐views surrounding this medical model are not imposed, cultural differences are respected, and timely access to interpreters provided.”*  **Reviewer’s comments:**  This study showed that former refugees in a regional Australian setting experienced significant stress due to challenges in securing housing.  **Assessment of methodological quality:**  This study has clearly met 8/10 (80%) criteria in the critical appraisal tool. |  |
| **Authors:**  Teariki (24)  **Year of publication:** 2017  **Country:**  New Zealand | **Sampling:**  This study included 14 Kiribati migrants living in New Zealand representing 91 family groups.  **Participants details:**  The participants included Kiribati adults with dependent children in the wider-Wellington region of New Zealand, specifically those who had migrated directly from Kiribati on a Kiribati passport, gained permanent residence, and lived in New Zealand for less than ten years.  **Setting/Context:**  This study was conducted within the Kiribati ethnic community in New Zealand, particularly in the Wellington area. | Qualitative research design using in-depth, face-to-face interviews and employing constructivist grounded theory. | This study aimed to understand the settlement experiences of Kiribati migrants in New Zealand, with a specific focus on the influence of housing on their health and well-being. | The study presents its findings under key themes briefly described below:   - **Early housing experiences:** Initially, Kiribati migrants often lived in overcrowded conditions with family or friends. This overcrowding is linked to health issues, especially in children, including respiratory illnesses and skin infections. The stress of living in such conditions, along with poor ventilation and unfamiliar living arrangements, could also impact mental health, contributing to feelings of stress and anxiety. - **Entry into the private rental market:** As migrants entered the private rental market, many faced challenges such as inadequate heating, dampness, and pest infestations in their rental homes. These conditions are associated with negative health outcomes, including increased respiratory problems such as asthma, chest infections, and other illnesses. The stress of navigating the rental market, especially for those with limited English proficiency or understanding of their rights as tenants, also posed mental health challenges. - **Quality of housing**: Poor quality housing, characterised by cold, damp, and inadequate maintenance, led to health issues such as worsened asthma and other respiratory conditions. This study highlighted the mental toll on migrants who struggled to provide better living conditions for their families, leading to feelings of helplessness and affecting their mental wellbeing. - **Home ownership:** Homeownership was seen as a significant achievement and a factor contributing to a sense of stability and belonging. The transition from inadequate rental housing to owning a home is associated with improvements in both physical and mental health. Living in a self-owned, possibly better-maintained home can reduce the health risks associated with poor rental conditions. Additionally, the security and pride of homeownership can positively impact mental health, reducing stress and anxiety related to housing instability. | **Author’s conclusions:**  *“Detailed information about how this migrant group entered the private rental housing market, by taking over the rental leases of other Kiribati migrants vacating their rental properties, indicated some of the unintended consequences related to a lack of incentives for landlords to make improvements. With the most vulnerable families most at risk from inadequate housing, this research concludes that there is a need for minimum housing standards to protect tenants.”*  **Reviewer’s comments:**  This study included Kiribati migrants in New Zealand, and revealed that their housing experiences, from initial overcrowding to challenges in the rental market and the eventual goal of homeownership, significantly impacted their physical and mental health, with conditions such as respiratory illnesses and stress being prevalent due to housing quality and stability.  **Assessment of methodological quality:**  This study has clearly met 9/10 (90%) criteria in the critical appraisal tool. |  |
| **Authors:**  Warfa et al. (25)  **Year of publication:** 2006  **Country:**  UK | **Sampling:**  Participants included 13 Somali professionals and 21 Somali laypeople living in East and South London, who were recruited through local Somali professional networks and community venues such as cafes and mosques.  **Participant details:**  Participants were from varied socio-economic backgrounds and included both genders.  **Setting/Context:**  This study was conducted in East and South London, focusing on the Somali refugee community’s experience with residential mobility. | Qualitative study using in-depth focus group discussions, conducted in both Somali and English, with subsequent translation where necessary. | This study aimed to explore the perceptions and realities of geographical mobility among Somali refugees in London and its relation to mental health and health service utilisation. | The study presents its findings under key themes, with only the relevant themes briefly described below:   - **Residential movements: Narratives of why and how** - **Perceived effects of mobility on mental health and health service utilisation:** Residential instability was seen as a significant stressor, contributing to poor mental health such as anxiety and distress. This was compounded by other factors such as homelessness, traumatic experiences, and community fragmentation. Frequent moves disrupted continuous access to healthcare. There were difficulties in registering with new GPs and a lack of cultural understanding in healthcare provision. | **Author’s conclusions:**  *“Frequent geographical movements were seen as stressful and undesirable, disrupted family life and child development and were detrimental to well being. Residential mobility was also perceived to interfere with health care receipt and therefore should be more comprehensively assessed in larger quantitative studies.”*  **Reviewer’s comments:**  Housing inequalities experienced by Somali people living in East and South London can lead to mental health issues such as stress, anxiety and depression, and hinder access to healthcare. Additionally, the focus on transience highlights this as a critical component of the precarity faced by migrants, contributing significantly to the challenges faced by migrants.  **Assessment of methodological quality:**  This study has clearly met 9/10 (90%) criteria in the critical appraisal tool. |  |
| **Authors:**  Whitehouse et al. (26)  **Year of publication:**  2021  **Country:**  Belgium | **Sampling:**  In this study, 41 in-depth interviews were conducted with 29 asylum seeker residents and 12 staff members in two reception centres in Belgium. Purposive sampling was undertaken.  **Participants details:**  Participants were asylum seekers residing in Belgium, including individuals of varying nationalities, genders, and family statuses, as well as staff members with different job profiles.  **Setting/Context:**  This study was conducted in two reception centres managed by the Federal Agency for the Reception of Asylum Seekers (Fedasil) in Charleroi and Morlanwelz, Belgium. | Qualitative study using in-depth interviews, conducted in English, French, Arabic, or Dari, and thematic analysis was utilised. | This study aimed to explore post-migration stressors during residency in reception centres and formulate recommendations for adequate service provision in Belgium. | The study presents its findings under key themes, with only the relevant themes briefly described below:   - **Poor living conditions:** Poor living conditions, including lack of privacy, overcrowding, and unhygienic conditions, had negative impacts on mental health. - **Lack of engagement, integration and autonomy** - **Inadequate capacity and resources to provide psychosocial support** | **Author’s conclusions:**  *“Analysis indicates that structural and practical challenges to adequately support asylum seekers are rooted in policy failures necessary for appropriate resourcing and prioritization of preventative measures. Such deliberate decisions contribute towards state deterrence strategies, eroding both individual well-being and manufacturing a crisis in the systems of support for asylum seekers.”*  **Reviewer’s comments:**  The study findings indicate that in Belgium, asylum seekers experience a lack of privacy, overcrowding, and unhygienic conditions, leading to negative impacts on their mental health due to living conditions.  **Assessment of methodological quality:**  This study has clearly met 8/10 (80%) criteria in the critical appraisal tool. |  |
| **Authors:**  Ziersch et al. (27)  **Year of publication:** 2017  **Country:**  Australia | **Sampling:**  Semi-structured interviews with 50 participants in South Australia, including 28 refugees and 22 asylum seekers. These individuals were purposively selected based on gender, continent of origin, and visa status, ensuring diverse representation.  **Participants details:**  The participants were refugees and asylum seekers living in South Australia, having arrived in Australia with either permanent protection visas or as asylum seekers on temporary visas, coming from varied cultural backgrounds, including the Middle East, Africa, and Southeast Asia.  **Setting/Context:**  This qualitative study was conducted in South Australia, specifically focusing on the experiences of refugees and asylum seekers within this region. | Qualitative study conducted using in-depth, semi-structured interviews. | This study aimed to explore the relationship between housing and health for refugees and asylum seekers in South Australia and to understand how various aspects of housing impact the health and wellbeing of these populations, particularly focusing on affordability, physical and social aspects of housing, and security of tenure. | The study presents its findings under key themes briefly described below:   - **Overall health and wellbeing:** Housing is vital for mental health, especially for individuals with complex health issues due to trauma and displacement. - **Affordability:** High housing costs contribute to financial strain, affecting mental wellbeing. Participants struggle to find affordable housing that meets their needs, impacting their mental health. - **Physical elements:** - ***Cold and damp*:** Poor housing conditions, including cold and dampness, lead to physical health issues such as respiratory problems. - ***Space and layout*:** Overcrowding and inadequate space negatively affect mental health. - **Social environment:** - ***Safety and disorder*:** Safety concerns in neighbourhoods impact mental health due to increased fear and anxiety. - ***Social connections*:** Positive relationships with neighbours are crucial for mental wellbeing, while discrimination and poor relations lead to negative mental health. - **Insecurity of tenure:** The instability in housing tenure, particularly in the private rental sector, causes anxiety and stress, affecting mental health. | **Author’s conclusions:**  *“Our research reinforces the importance of housing for both the physical and mental health for asylum seekers and refugees living in resettlement countries. Improving housing quality, affordability and tenure security all have the potential to lead to more positive health outcomes.”*  **Reviewer’s comments:**  The study findings suggest that for refugees and asylum seekers living in South Australia, housing factors such as affordability stress, inadequate conditions, neighbourhood safety concerns, and tenure insecurity profoundly impact their wellbeing and integration process. Moreover, this study makes a useful distinction between structural elements (e.g., housing quality), and operational elements (e.g., tenure security), which collectively influence the overall housing experience and its effects on well-being.  **Assessment of methodological quality:**  This study has clearly met 9/10 (90%) criteria in the critical appraisal tool. |  |
| **Authors:**  Ziersch et al. (28)  **Year of publication:** 2024  **Country:**  Australia | **Sampling:**  In this study, 25 participants were recruited from a larger study on housing, social inclusion, and health for asylum seekers and refugees in Australia, which surveyed over 400 individuals and conducted follow-up interviews.  **Participants details:**  Participants included men and women from the Middle East, Africa, and Southeast Asia, with diverse immigration statuses such as bridging visas, temporary refugee visas, permanent visas, and citizenship. The time participants had spent in Australia ranged from 2.5 to 10 years across the interview rounds.  **Setting/Context:**  Interviews were conducted in South Australia during three waves between 2016 and 2020. Participants were living under varying housing and visa conditions, reflecting the broader challenges faced by asylum seekers and refugees in Australia, such as limited housing support and reliance on the private rental market. | Longitudinal qualitative design with three rounds of interviews over approximately four years and analysed using a critical realist approach and framework thematic analysis. | The study aimed to investigate the relationship between housing, social inclusion and health among asylum seekers and refugees in Australia, focusing on how housing conditions influenced participant's health and well-being over time. | The study presents its findings under key themes which are briefly described below:   - **Physical environment:** Challenges with unsuitable physical environments, such as overcrowding, mould, and noisy locations, significantly impacted participant's physical and mental health, exacerbating stress, ontological insecurity, and financial precarity. Conversely, improvements in housing conditions, privacy, and personal control fostered better health outcomes and a sense of stability, despite the enduring challenges of forced migration and resettlement**.** - **Instability:** Housing insecurity, especially among Temporary Visa holders, was linked to negative mental and physical health outcomes, including stress, sleep issues, and breathing difficulties. In contrast, participants with stable housing and secure immigration status experienced improved well-being and a greater sense of belonging. - **Safety:** Safety in neighbourhoods was identified as a major factor influencing health and ontological security, with many participants relocating to safer areas to improve their well-being. Some participants continued to face threats to their safety, which exacerbated mental and physical stress. - **Social connections, support and services:** Housing affordability and suitability significantly influenced health outcomes through proximity to social connections, support, and services, with some participants struggling to develop these bonds, while others thrived in supportive communities. Positive relationships with neighbours and access to services were key factors in rebuilding ontological security, enhancing emotional and practical support, and improving overall well-being. | **Author’s conclusions:**  *"Rebuilding a sense of home and ontological security is a key resettlement priority and crucial for well- being. More comprehensive strategies to facilitate this for refugees and asylum seekers are required."*  **Reviewer’s comments:**  The findings of the study suggest that housing conditions, including physical environment, stability, safety, and proximity to social support, significantly impact mental and physical health, with improved housing fostering better well-being and ontological security, while insecurity and poor conditions exacerbate stress and health challenges.  **Assessment of methodological quality:**  This study has clearly met 10/10 (100%) criteria in the critical appraisal tool. |  |
| **Abbreviations:**  B&Bs: Bed and Breakfasts; CBPR: Community-based Participatory Research; FEDASIL: Federal Agency for the Reception of Asylum Seekers; FGDs: Focus Group Discussions; GP: General Practitioner; MDS: Multidimensional Scaling; PTSD: Post-Traumatic Stress Disorder; UAE: United Arab Emirates; UK: United Kingdom; US: United States of America; YWS: Youth Without Shelter.  **Definitions:**  **Asylum Processing Centres:** Facilities where the claims of individuals seeking asylum are processed and evaluated. **Asylum Seeker:** An individual who is seeking international protection from dangers in their home country but whose claim for refugee status hasn’t yet been determined. **Constructivist Grounded Theory:** A version of grounded theory that emphasises the role of the researcher's interpretation and the co-construction of meaning with participants. **Ethnography:** A qualitative research method where researchers observe and interact with a study's participants in their real-life environment. **Focus Group Discussions (FGDs):** A qualitative research technique involving a group of individuals who are asked about their perceptions, opinions, beliefs, and attitudes towards a product, service, concept, advertisement, idea, or packaging. **Grounded Theory:** A research method in qualitative research that involves constructing theories through methodical gathering and analysis of data. **Gulf countries**: Members of Gulf Cooperation Council that are Bahrain, Kuwait, Oman, Qatar, Saudi Arabia and the UAE. **Mental Health Program:** A program that offers services and support for individuals dealing with mental health issues, including therapy, counselling, and other forms of treatment. **Participant Observation:** A key qualitative research method used in anthropology and sociology, whereby the researcher immerses themselves in a community or organisation to observe and interact with participants**.** **Photovoice Methodology:** A research technique in which participants capture their community or experiences using photography. The photographs are then used as a basis for discussion and qualitative research. **Qualitative Research:** A method of inquiry employed in several different academic disciplines, traditionally in the social sciences, but also in market research and further contexts, which aims to gather an in-depth understanding of human behaviour and the reasons that govern such behaviour. **Reception Centre:** A facility where individuals entering the asylum system are initially accommodated, typically for up to 18 months or longer, depending on the prospects of their asylum claim being accepted. **Refugee:** A person who has been forced to leave their country in order to escape war, persecution, or natural disaster and has been granted refugee status. **Thematic Analysis:** A method used in qualitative research which involves identifying themes and patterns within a dataset. | | | | | | |

**References:**

1. Devkota HR, Bhandari B, Adhikary P. Perceived mental health, wellbeing and associated factors among Nepali male migrant and non-migrant workers: A qualitative study. J Migr Health. 2021;3:100013.

2. Dhanji S. Social or Unsocial?: The Linkage between Accommodation, Health and Well-being among Former Horn of Africa and Sudanese Refugees Living in Australia. Australasian Review of African Studies, The. 2010;31(1):106-36.

3. Dhanji S. Welcome or Unwelcome?: Integration Issues and the Resettlement of Former Refugees from the Horn of Africa and Sudan in Metropolitan Melbourne. The Australasian Review of African Studies. 2009;30(2):152-78.

4. Dhesi S, Isakjee A, Davies T. Public health in the Calais refugee camp: environment, health and exclusion. Critical Public Health. 2018;28(2):140-52.

5. Due C, Ziersch A, Walsh M, Duivesteyn E. Housing and health for people with refugee-and asylum-seeking backgrounds: a photovoice study in Australia. Housing Studies. 2022;37(9):1598-624.

6. Fozdar F. ‘The Golden Country’: Ex-Yugoslav and African Refugee Experiences of Settlement and ‘Depression’. Journal of Ethnic and Migration Studies. 2009;35(8):1335-52.

7. Hanley J, Ives N, Lenet J, Hordyk S-R, Walsh C, Soltane SB, et al. Migrant women’s health and housing insecurity: an intersectional analysis. International Journal of Migration, Health and Social Care. 2019;15(1):90-106.

8. Haque N, Rosas S. Concept mapping of photovoices: sequencing and integrating methods to understand immigrants' perceptions of neighborhood influences on health. Fam Community Health. 2010;33(3):193-206.

9. Hashimoto-Govindasamy LS, Rose V. An ethnographic process evaluation of a community support program with Sudanese refugee women in western Sydney. Health Promot J Austr. 2011;22(2):107-12.

10. Holmes SM. An ethnographic study of the social context of migrant health in the United States. PLoS Med. 2006;3(10):e448.

11. Howden-Chapman P, Pene G, Crane J, Green R, Iupati L, Prior I, et al. Open houses and closed rooms: Tokelau housing in New Zealand. Health Educ Behav. 2000;27(3):351-62.

12. Keim-Malpass J, Spears Johnson CR, Quandt SA, Arcury TA. Perceptions of housing conditions among migrant farmworkers and their families: implications for health, safety and social policy. Rural Remote Health. 2015;15:3076.

13. Khan BM, Waserman J, Patel M. Perspectives of Refugee Youth Experiencing Homelessness: A Qualitative Study of Factors Impacting Mental Health and Resilience. Frontiers in Psychiatry. 2022;13.

14. Lauritzen C, Sivertsen H. Children and Families Seeking Asylum in Northern Norway: Living Conditions and Mental Health. International Migration. 2012;50(6):195-210.

15. Miller KE, Worthington GJ, Muzurovic J, Tipping S, Goldman A. Bosnian refugees and the stressors of exile: a narrative study. Am J Orthopsychiatry. 2002;72(3):341-54.

16. Mwanri L, Fauk NK, Ziersch A, Gesesew HA, Asa GA, Ward PR. Post-Migration Stressors and Mental Health for African Migrants in South Australia: A Qualitative Study. International Journal of Environmental Research and Public Health [Internet]. 2022; 19(13).

17. Palmer D, Ward K. 'Lost': listening to the voices and mental health needs of forced migrants in London. Med Confl Surviv. 2007;23(3):198-212.

18. Papadopoulos I, Lees S, Lay M, Gebrehiwot A. Ethiopian refugees in the UK: migration, adaptation and settlement experiences and their relevance to health. Ethn Health. 2004;9(1):55-73.

19. Rast E, Hintermeier M, Bozorgmehr K, Biddle L. Housing and health: A multidimensional, qualitative analysis of the experiences of asylum seekers and refugees living in German reception centres. SSM - Qualitative Research in Health. 2024;5:100407.

20. Regmi PR, Aryal N, van Teijlingen E, Simkhada P, Adhikary P. Nepali Migrant Workers and the Need for Pre-departure Training on Mental Health: A Qualitative Study. J Immigr Minor Health. 2020;22(5):973-81.

21. Rzepka I, Zehetmair C, Roether E, Kindermann D, Cranz A, Junne F, et al. Impact of and Coping with Post-Traumatic Symptoms of Refugees in Temporary Accommodations in Germany: A Qualitative Analysis. Int J Environ Res Public Health. 2022;19(17).

22. Sah LK, Burgess RA, Sah RK. 'Medicine doesn't cure my worries': Understanding the drivers of mental distress in older Nepalese women living in the UK. Glob Public Health. 2019;14(1):65-79.

23. Smith LA, Reynish T, Hoang H, Mond J, Hannah C, McLeod K, et al. The mental health of former refugees in regional Australia: A qualitative study. Aust J Rural Health. 2019;27(5):459-62.

24. Teariki MA. Housing and Health of Kiribati Migrants Living in New Zealand. Int J Environ Res Public Health. 2017;14(10).

25. Warfa N, Bhui K, Craig T, Curtis S, Mohamud S, Stansfeld S, et al. Post-migration geographical mobility, mental health and health service utilisation among Somali refugees in the UK: A qualitative study. Health & Place. 2006;12(4):503-15.

26. Whitehouse K, Lambe E, Rodriguez S, Pellecchia U, Ponthieu A, Van den Bergh R, et al. A qualitative exploration of post-migration stressors and psychosocial well-being in two asylum reception centres in Belgium. International Journal of Migration, Health and Social Care. 2021;17(3):241-58.

27. Ziersch A, Walsh M, Due C, Duivesteyn E. Exploring the Relationship between Housing and Health for Refugees and Asylum Seekers in South Australia: A Qualitative Study. Int J Environ Res Public Health. 2017;14(9).

28. Ziersch A, Walsh M, Due C. Housing and health for people from refugee and asylum-seeking backgrounds: findings from an Australian qualitative longitudinal study. BMC Public Health. 2024;24(1):1138.
