# Supplementary material for: Housing inequalities and health outcomes among migrant and refugee populations in high-income countries: a mixed-methods systematic review
Source: BMC Public Health. 2025 Mar 22;25:1098. doi: 10.1186/s12889-025-22186-5 (PMC11929249; doi:10.1186/s12889-025-22186-5)
Supplement: Supplementary file 5 — Supplementary Material 5 [file 12889_2025_22186_MOESM5_ESM.docx]

**Supplementary Table S5.** Assessment of methodological quality of studies reviewed

| **Quantitative studies** | | | | | | | | | | | | | |
| --- | --- | --- | --- | --- | --- | --- | --- | --- | --- | --- | --- | --- | --- |
|  | **Cohort studies** | **1. Were the two groups similar and recruited from the same population?** | **2. Were the exposures measured similarly to assign people to both exposed and unexposed groups?** | **3. Was the exposure measured in a valid and reliable way?** | **4. Were confounding factors identified?** | **5. Were strategies to deal with confounding factors stated?** | **6. Were the groups/participants free of the outcome at the start of the study (or at the moment of exposure)?** | **7. Were the outcomes measured in a valid and reliable way?** | **8. Was the follow up time reported and sufficient to be long enough for outcomes to occur?** | **9. Was follow up complete, and if not, were the reasons to loss to follow up described and explored?** | **10. Were strategies to address incomplete follow up utilised?** | **11. Was appropriate statistical analysis used?** | **Overall quality** |
| 1 | Ahmad et al. (2021) | Yes | Yes | Yes | Yes | No | No | Yes | Yes | Unclear | Unclear | Yes | 7/11 (64%) |
| 2 | Ambrosetti et al. (2021) | Yes | Yes | Yes | Yes | Yes | No | Yes | Yes | No | No | Yes | 8/11 (73%) |
| 3 | Campbell et al. (2018) | Yes | Yes | Yes | Yes | Yes | No | Yes | Yes | No | Unclear | Yes | 8/11 (73%) |
| 4 | Eisen et al. (2021) | Yes | Yes | Yes | Yes | Yes | No | Yes | Yes | No | No | Yes | 8/11 (73%) |
| 5 | Gillespie et al. (2020) | Yes | Yes | Yes | Unclear | No | No | Yes | Yes | Yes | Unclear | Yes | 7/11 (64%) |
| 6 | Handiso et al. (2024) | Yes | Yes | Yes | Yes | Yes | No | Yes | Yes | No | Yes | Yes | 9/11 (82%) |
| 7 | Handiso et al. (2024) | Yes | Yes | Yes | Yes | Yes | No | Yes | Yes | No | Yes | Yes | 9/11 (82%) |
| 8 | Martino et al. (2022) | Yes | Yes | Yes | Yes | Yes | No | Yes | Yes | Yes | Yes | Yes | 10/11 (91%) |
| 9 | Raphael et al. (2020) | Yes | Yes | Yes | Yes | Yes | Unclear | Yes | Yes | Unclear | Unclear | Yes | 8/11 (73%) |
| 10 | Whitsett and Sherman (2017) | Yes | Yes | Yes | Yes | Yes | No | Yes | Yes | Unclear | Unclear | Yes | 8/11 (73%) |

|  | **Quantitative studies** | | | | | | | | | |
| --- | --- | --- | --- | --- | --- | --- | --- | --- | --- | --- |
|  | **Cross-sectional studies** | **1. Were the criteria for inclusion in the sample clearly defined?** | **2. Were the study subjects and the setting described in detail?** | **3. Was the exposure measured in a valid and reliable way?** | **4. Were objective, standard criteria used for measurement of the condition?** | **5. Were confounding factors identified?** | **6. Were strategies to deal with confounding factors stated?** | **7. Were the outcomes measured in a valid and reliable way?** | **8. Was appropriate statistical analysis used?** | **Overall quality** |
| 11 | Al Masri et al. (2021) | Yes | Yes | Yes | Yes | Yes | Unclear | Yes | Yes | 7/8 (88%) |
| 12 | Bayes-Marin et al. (2022) | Yes | Yes | Yes | Yes | Yes | Yes | Yes | Yes | 8/8 (100%) |
| 13 | Blukacz et al. (2024) | Yes | Yes | Yes | Yes | Yes | Yes | Yes | Yes | 8/8 (100%) |
| 14 | Cabieses et al. (2012) | Yes | Yes | Yes | Yes | Yes | Yes | Yes | Yes | 8/8 (100%) |
| 15 | Cloos et al. (2020) | Yes | Yes | Yes | Yes | Yes | Yes | Yes | Yes | 8/8 (100%) |
| 16 | Dudek et al. (2022) | Yes | Yes | Yes | Yes | Yes | Yes | Yes | Yes | 8/8 (100%) |
| 17 | Kang et al. (2022) | Yes | Yes | Yes | Yes | Yes | Yes | Yes | Yes | 8/8 (100%) |
| 18 | Kearney et al. (2014) | Yes | Yes | Yes | Yes | Yes | No | Yes | Yes | 7/8 (88%) |
| 19 | Litt et al. (2010) | Yes | Yes | Yes | Yes | Yes | Unclear | Yes | Yes | 7/8 (88%) |
| 20 | Mangrio and Zdravkovic (2018) | Yes | Yes | Yes | Yes | Yes | Yes | Yes | Yes | 8/8 (100%) |
| 21 | Marchi et al. (2022) | Yes | Yes | Yes | Yes | Yes | Yes | Yes | Yes | 8/8 (100%) |
| 22 | Mendola and Busetta (2018) | Yes | Yes | Yes | Yes | Yes | Yes | Yes | Yes | 8/8 (100%) |
| 23 | Mohsenpour et al. (2023) | Yes | Yes | Yes | Yes | Yes | Yes | Yes | Yes | 8/8 (100%) |
| 24 | Mohsenpour et al. (2023) | Yes | Yes | Yes | Yes | Yes | Yes | Yes | Yes | 8/8 (100%) |
| 25 | Montazer (2022) | Yes | Yes | Yes | Yes | Yes | Yes | Yes | Yes | 8/8 (100%) |
| 26 | Mora et al. (2016) | Yes | Yes | Yes | Yes | Yes | Yes | Yes | Yes | 8/8 (100%) |
| 27 | Oudin et al. (2016) | Yes | Yes | Yes | Yes | Yes | Yes | Yes | Yes | 8/8 (100%) |
| 28 | Richter et al. (2018) | Yes | Yes | Yes | Yes | Yes | Yes | Yes | Yes | 8/8 (100%) |
| 29 | Sandberg et al. (2014) | Yes | Yes | Yes | Yes | Yes | Yes | Yes | Yes | 8/8 (100%) |
| 30 | Song et al. (2015) | Yes | Yes | Yes | Yes | Yes | Yes | Yes | Yes | 8/8 (100%) |
| 31 | Song et al. (2018) | Yes | Yes | Yes | Yes | Yes | Yes | Yes | Yes | 8/8 (100%) |
| 32 | Srirangson et al. (2013) | Yes | Yes | Yes | Yes | Yes | Yes | Yes | Yes | 8/8 (100%) |
| 33 | Sundquist et al. (1995) | Yes | Yes | Yes | Yes | Yes | Yes | Yes | Yes | 8/8 (100%) |
| 34 | Tortelli et al. (2021) | Yes | Yes | Yes | Yes | Yes | Yes | Yes | Yes | 8/8 (100%) |
| 35 | Vignier et al. (2022) | Yes | Yes | Yes | Yes | Yes | Yes | Yes | Yes | 8/8 (100%) |
| 36 | Walther et al. (2020) | Yes | Yes | Yes | Yes | Yes | Yes | Yes | Yes | 8/8 (100%) |
| 37 | Walther et al. (2020) | Yes | Yes | Yes | Yes | Yes | Yes | Yes | Yes | 8/8 (100%) |
| 38 | Wirehag et al. (2021) | Yes | Yes | Yes | Yes | Unclear | Unclear | Yes | Yes | 6/8 (75%) |

| **Qualitative studies** | | | | | | | | | | | | |
| --- | --- | --- | --- | --- | --- | --- | --- | --- | --- | --- | --- | --- |
|  |  | **1. Is there congruity between the stated philosophical perspective and the research methodology?** | **2. Is there congruity between the research methodology and the research question or objectives?** | **3. Is there congruity between the research methodology and the methods used to collect data?** | **4. Is there congruity between the research methodology and the representation and analysis of data?** | **5. Is there congruity between the research methodology and the interpretation of results?** | **6. Is there a statement locating the researcher culturally or theoretically?** | **7. Is the influence of the researcher on the research, and vice- versa, addressed?** | **8. Are participants, and their voices, adequately represented?** | **9. Is the research ethical according to current criteria or, for recent studies, and is there evidence of ethical approval by an appropriate body?** | **10. Do the conclusions drawn in the research report flow from the analysis, or interpretation, of the data?** | **Overall quality** |
| 1 | Devkota et al. (2021) | Unclear | Yes | Yes | Yes | Yes | No | No | Yes | Yes | Yes | 7/10 (70%) |
| 2 | Dhanji (2010) | Yes | Yes | Yes | Yes | Yes | No | No | Yes | Yes | Yes | 8/10 (80%) |
| 3 | Dhesi et al. (2018) | Yes | Yes | Yes | Yes | Yes | No | No | No | Yes | Yes | 7/10 (70%) |
| 4 | Due et al. (2022) | Yes | Yes | Yes | Yes | Yes | Yes | No | Yes | Yes | Yes | 9/10 (90%) |
| 5 | Fozdar (2009) | Yes | Yes | Yes | Yes | Yes | No | No | Yes | Unclear | Yes | 7/10 (70%) |
| 6 | Hanley et al. (2019) | Yes | Yes | Yes | Yes | Yes | Yes | Yes | Yes | Yes | Yes | 10/10 (100%) |
| 7 | Haque and Rosas (2010) | Yes | Yes | Yes | Yes | Yes | No | No | Yes | Unclear | Yes | 7/10 (70%) |
| 8 | Hashimoto-Govindasamy and Rose (2011) | Yes | Yes | Yes | Yes | Yes | Unclear | Yes | Yes | Yes | Yes | 9/10 (90%) |
| 9 | Holmes (2006) | Yes | Yes | Yes | Yes | Yes | Yes | Yes | Yes | Yes | Yes | 10/10 (100%) |
| 10 | Howden-Chapman et al. (2000) | Yes | Yes | Yes | Yes | Yes | No | No | Yes | Yes | Yes | 8/10 (80%) |
| 11 | Keim-Malpass et al. (2015) | Yes | Yes | Yes | Yes | Yes | Yes | Yes | Yes | Yes | Yes | 10/10 (100%) |
| 12 | Khan et al. (2022) | Yes | Yes | Yes | Yes | Yes | No | No | Yes | Yes | Yes | 8/10 (80%) |
| 13 | Lauritzen and Sivertsen (2012) | Yes | Yes | Yes | Yes | Yes | Unclear | Unclear | Yes | Unclear | Yes | 7/10 (70%) |
| 14 | Miller et al. (2002) | Yes | Yes | Yes | Yes | Yes | Unclear | Unclear | Yes | Unclear | Yes | 7/10 (70%) |
| 15 | Mwanri et al. (2022) | Yes | Yes | Yes | Yes | Yes | Yes | No | Yes | Yes | Yes | 9/10 (90%) |
| 16 | Palmer and Ward (2007) | Yes | Yes | Yes | Yes | Yes | Yes | Yes | Yes | Yes | Yes | 10/10 (100%) |
| 17 | Papadopoulos et al. (2004) | Yes | Yes | Yes | Yes | Yes | Yes | Yes | Yes | Yes | Yes | 10/10 (100%) |
| 18 | Rast et al. (2024) | Yes | Yes | Yes | Yes | Yes | Yes | Unclear | Yes | Yes | Yes | 9/10 (90%) |
| 19 | Regmi et al. (2020) | Yes | Yes | Yes | Yes | Yes | No | No | Yes | Yes | Yes | 8/10 (80%) |
| 20 | Rzepka et al. (2022) | Yes | Yes | Yes | Yes | Yes | No | No | Yes | Yes | Yes | 8/10 (80%) |
| 21 | Sah et al. (2019) | Yes | Yes | Yes | Yes | Yes | Yes | Unclear | Yes | Yes | Yes | 9/10 (90%) |
| 22 | Smith et al. (2019) | Yes | Yes | Yes | Yes | Yes | No | No | Yes | Yes | Yes | 8/10 (80%) |
| 23 | Teariki (2017) | Yes | Yes | Yes | Yes | Yes | Yes | Yes | Yes | Unclear | Yes | 9/10 (90%) |
| 24 | Warfa et al. (2006) | Yes | Yes | Yes | Yes | Yes | Yes | No | Yes | Yes | Yes | 9/10 (90%) |
| 25 | Whitehouse et al. (2021) | Yes | Yes | Yes | Yes | Yes | No | No | Yes | Yes | Yes | 8/10 (80%) |
| 26 | Ziersch et al. (2017) | Yes | Yes | Yes | Yes | Yes | No | Yes | Yes | Yes | Yes | 9/10 (90%) |
| 27 | Ziersch et al. (2024) | Yes | Yes | Yes | Yes | Yes | Yes | Yes | Yes | Yes | Yes | 10/10 (100%) |
